# Rapid Transmission and Divergence of Vancomycin-Resistant *Enterococcus faecium* Sequence Type 80, China

**DOI:** 10.3201/eid3105.241649

**Published:** 2025-05

**Authors:** Liqiang Li, Xingwei Wang, Yanyu Xiao, Bing Fan, Jiehong Wei, Jie Zhou, Zetian Lai, Yanpeng Zhang, Hongmei Mo, Li Zhang, Dixian Luo, Dayong Gu, Shucai Yang, Yidi Wang, Jiuxin Qu, Shenzhen Pathogen Infection Research Alliance (SPIRA)

**Affiliations:** Shenzhen Third People’s Hospital Department of Clinical Laboratory; National Clinical Research Center for Infectious Diseases, Second Affiliated Hospital of Southern University of Science and Technology, Shenzhen, China (L. Li, X. Wang, Y. Xiao, Y. Wang, J. Qu); Shenzhen Institute of Translational Medicine, First Affiliated Hospital of Shenzhen University, Shenzhen Second People’s Hospital, Shenzhen (B. Fan, Y. Zhang, D. Gu); Medical Laboratory of the Third Affiliated Hospital of Shenzhen University, Shenzhen (J. Wei, H. Mo); Pingshan Hospital, Southern Medical University, Pingshan District People’s Hospital of Shenzhen, Shenzhen (J. Zhou, L. Zhang, S. Yang); Shenzhen Nanshan People's Hospital, Shenzhen (Z. Lai, D. Luo); Shenzhen University Medical School, Shenzhen (Z. Lai, D. Luo)

**Keywords:** antimicrobial resistance, vancomycin resistance, bacteria, *Enterococci faecium*, hospital-acquired infection, nosocomial infection, whole-genome sequencing, epidemiology, China

## Abstract

We investigated genomic evolution of vancomycin-resistant *Enterococcus faecium* (VREF) during an outbreak in Shenzhen, China. Whole-genome sequencing revealed 2 sequence type 80 VREF subpopulations diverging through insertion sequence–mediated recombination. One subpopulation acquired more antimicrobial resistance and carbohydrate metabolism genes. Persistent VREF transmission underscores the need for genomic surveillance to curb spread.

Vancomycin-resistant *Enterococcus faecium* (VREF) causes hospital-acquired infections worldwide and poses a threat to public health (*1*). Whole-genome sequencing (WGS) has demonstrated that new healthcare-associated *E. faecium* clones rapidly emerge, evolve, and adapt through intragenus recombination, displacing existing clones (*2*,*3*).

During the past decade, clonal complex 17 sequence type (ST) 80 rapidly became the predominant VREF lineage in Denmark (*4*), Australia (*5*), Ireland (*6*), Spain (*7*), and Sweden (*8*) and accounted for 40%–67.1% of VREF isolates disseminated in hospital settings. Few ST80 cases were reported in Asia countries, including China, until an independent lineage of ST80, sequence cluster (SC)11, emerged in January 2021 as the predominant cause of an ongoing VREF outbreak in Guangdong Province (*9*) and posed a risk of spreading to other areas. However, the pangenomic features and adaptation potential of the emerging SC11 remain unknown.

VREF isolation rates also substantially increased in the metropolitan city of Shenzhen, Guangdong Province, China, during 2021–2024. VREF isolation rates before 2021 remained <6% (predominantly <5%) but rose to an average of 11.53% in 2024 (Appendix 1 Figure 1, panel A). To trace the source and characterize genomic features that potentially contributed to outbreaks, we conducted a multicenter genomic epidemiology study and integrated pangenomic variation analysis.

**Figure 1:**
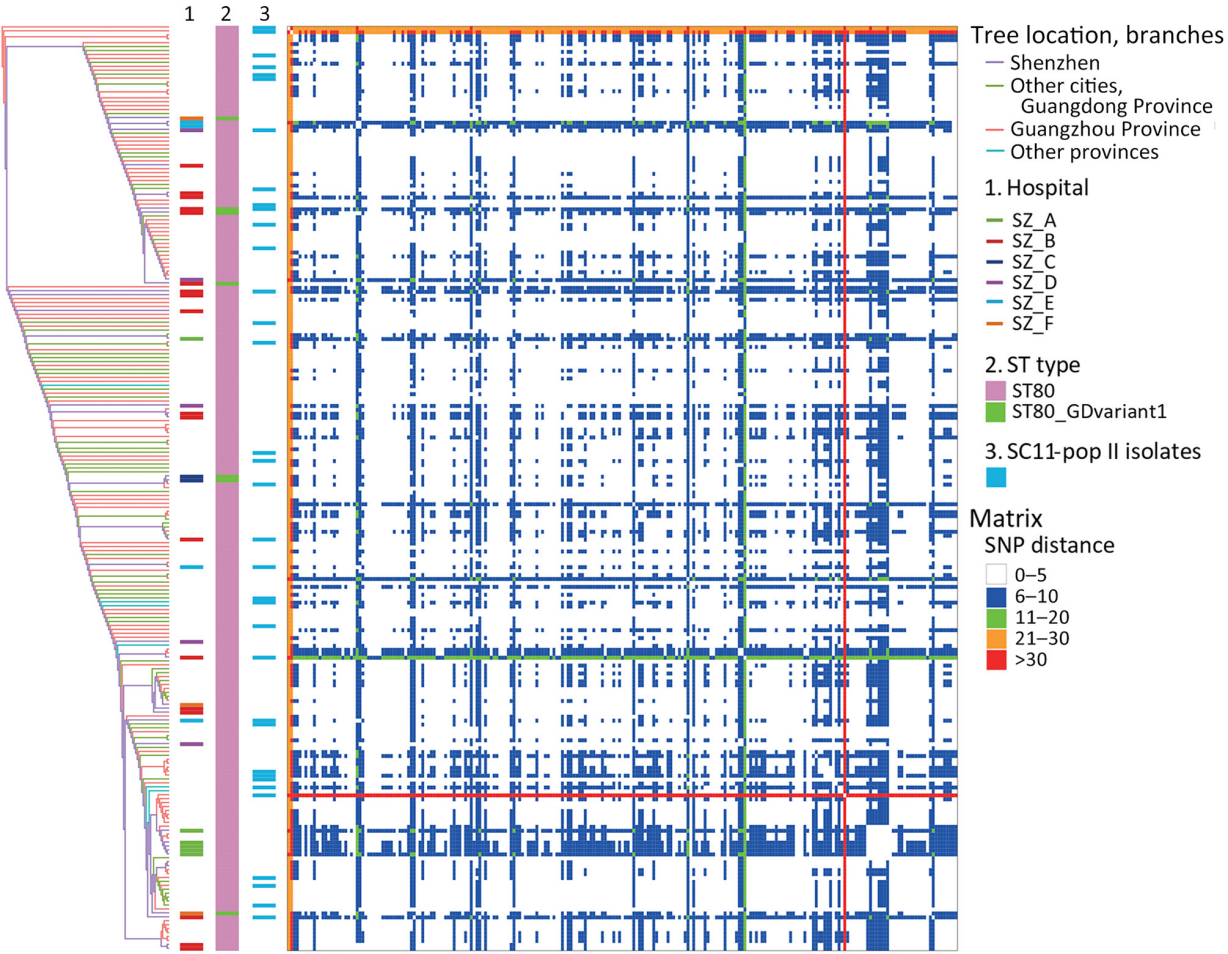
Evolution and variation of SC11 inferred from core genome SNPs during rapid transmission and divergence of vancomycin-resistant *Enterococcus faecium* ST80, China. Graphs shows reconstructed tree from core genome SNPs (left) among all SC11 isolates (n = 235) using strain SZYSC_GYSVRE003 (GenBank accession no. GCA_037475005.1) as reference. Hospital sources of isolates from Shenzhen ST80 and its variant, and SC11-pop II isolates are indicated (1, 2, and 3) next to the tree. The heatmap (right) shows pairwise SNP distance matrix indicating diversity of SC11 lineage presented in the form of symmetry in the bottom left and top right. Cells in the heatmap are colored to show SNP distance in a graded gradient. Red lines indicate large SNP distances and correspond to SZYSC_23VRE019 in the tree. pop, population; SC, sequence cluster; SNP, single-nucleotide polymorphism; ST, sequence type.

## The Study

We performed WGS analysis (Appendix 1) on 42 VREF isolates (primarily collected after April 2022) from 39 patients across 7 hospitals, designated SZ_A through SZ_F, including 2 affiliated hospitals, SZ_C1 and SZ_C2, grouped as SZ_C. We used WGS to identify STs and used phylogenetic analysis to determine ST sources in a global context. We assessed genetic diversity, indicating mutation rates during circulation, using pairwise core genome single-nucleotide polymorphism (cgSNP) distance. We characterized population structure to show divergence and emergence of novel variant populations.

**Figure 2:**
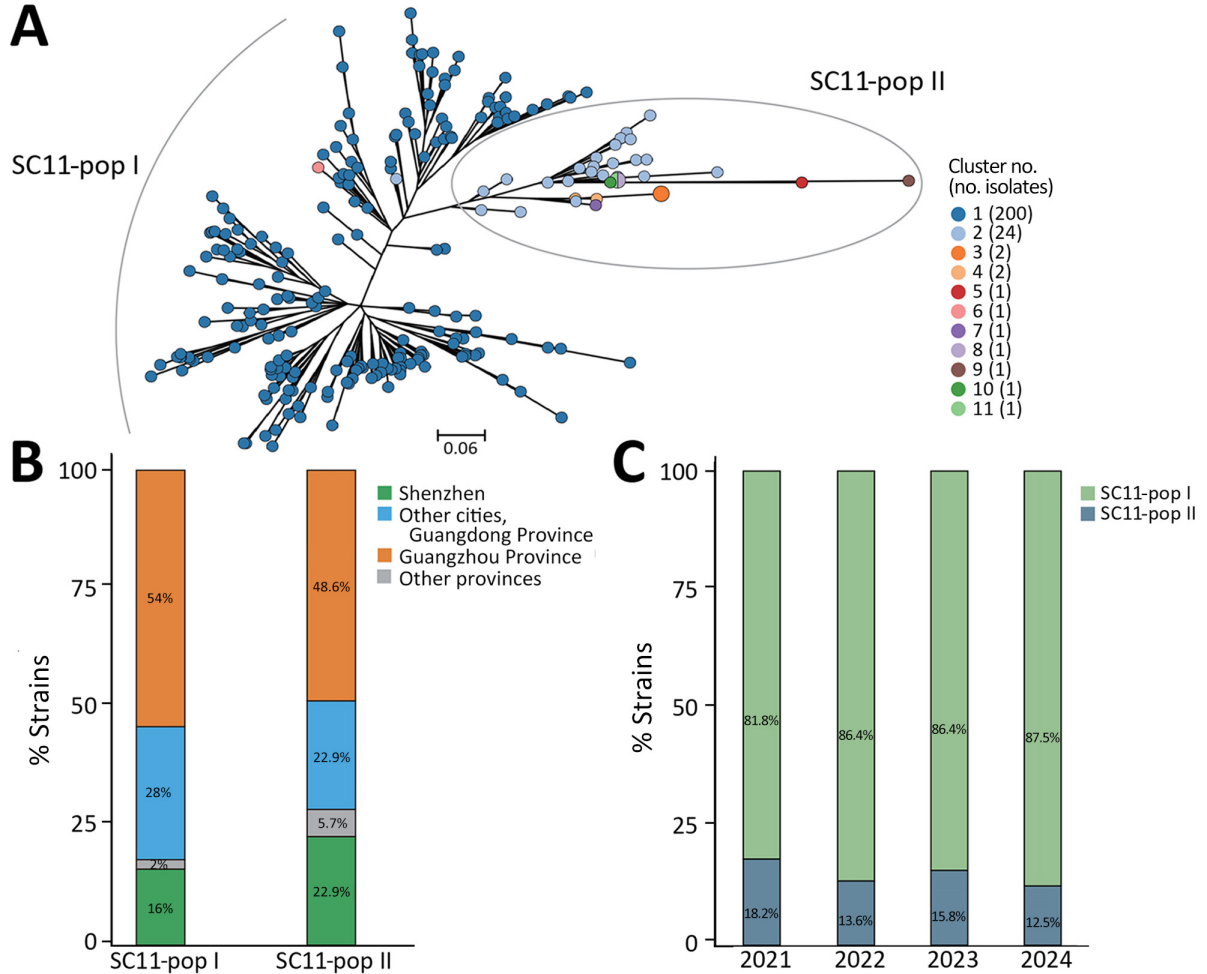
Lineages detected in a study of rapid transmission and divergence of vancomycin-resistant *Enterococcus faecium* ST80, China, showing 2 lineages circulating in parallel. A) SC11 subpopulations labeled on pangenomic tree inferred from gene presence and absence matrix. Eleven clusters were delineated in PopPUNK, labeled in different colors. Clusters 1 and 2 are the 2 major populations. Cluster 6 (1 strain) is nested in cluster 1 (200 strains) on the pangenomic tree and thus are denoted together as SC11-pop I, whereas strains from clusters 3–11 (except cluster 6, 10 strains together) are nested in cluster 2 (24 strains) and are denoted as SC11-pop I. Scale bar is unit of tree branch length, indicating the genetic distance stimulated from gene presence and absence matrix using roary (https://sanger-pathogens.github.io/Roary). B) Parallel circulation of SC11-pop I and SC11-pop II strains from 2021 to 2024. C) Geographic distribution of SC11-pop I (n = 200) and SC11-pop II (n = 35) strains. Prevalence is displayed in percentage. pop, population; SC, sequence cluster; ST, sequence type.

Among the 42 isolates, 34 (81%) were ST80 isolates, 7 (17%) were ST80 variant isolates (ST80_GDvariant1) with *ddl* loci mutated from 1 to 194, and 1 (2%) was an ST78 isolate that was collected in 2023 (Appendix 1 Figure 1, panel B; Appendix 2). Unexpectedly, ST80_GDvariant1 isolates independently emerged in 5 branches and did not originate from a single mutation event (Figure 1; Appendix 1 Figure 2, panel A).

Phylogenetic analysis inferred from cgSNP of the 41 isolates (ST80 and variants) and 703 other publicly available ST80 isolates revealed that the 41 isolates from the ongoing outbreak in Guangdong are affiliated with SC11 (Appendix 1 Table 2, Figure 2, panel A) (*9*). Within SC11, two 2022 strains from Guangzhou, SZYSC_ZDYVRE007 and SZYSC_ZDYVRE008G, with a 5-SNP divergence, clustered adjacent to the lineage root (Figure 1; Appendix 1 Figure 2, panel A) and formed a distinct VREF sublineage (SC11-root sublineage) with a 37-SNP average divergence from other SC11 strains (bootstrap = 72) (Figure 1). In contrast, the remaining SC11 strains exhibited tight clustering (pairwise distances of <5 SNPs [36.5%] and 5–10 SNPs [49.8%]) and formed a dominant clade (SC11-outbreak sublineage) (Figure 1). Those findings suggest that the SC11-outbreak lineage originated from a single progenitor or highly related lineage, enabling rapid transmission. 

To resolve strain differentiation, we analyzed SC11-outbreak_lineage population structure by using PopPUNK (https://github.com/bacpop/PopPUNK), which integrates core and accessory genomic variation (*10*). We delineated 2 subpopulations, SC11-pop I and SC11-pop II (Figure 2, panel A; Appendix 1 Figure 4). SC11-pop II isolates formed tight clusters in the pangenome-based tree (Figure 2, panel A) but dispersed in the cgSNP phylogeny (Figure 1), suggesting subpopulation divergence was primarily driven by gene gain or loss.

**Figure 3:**
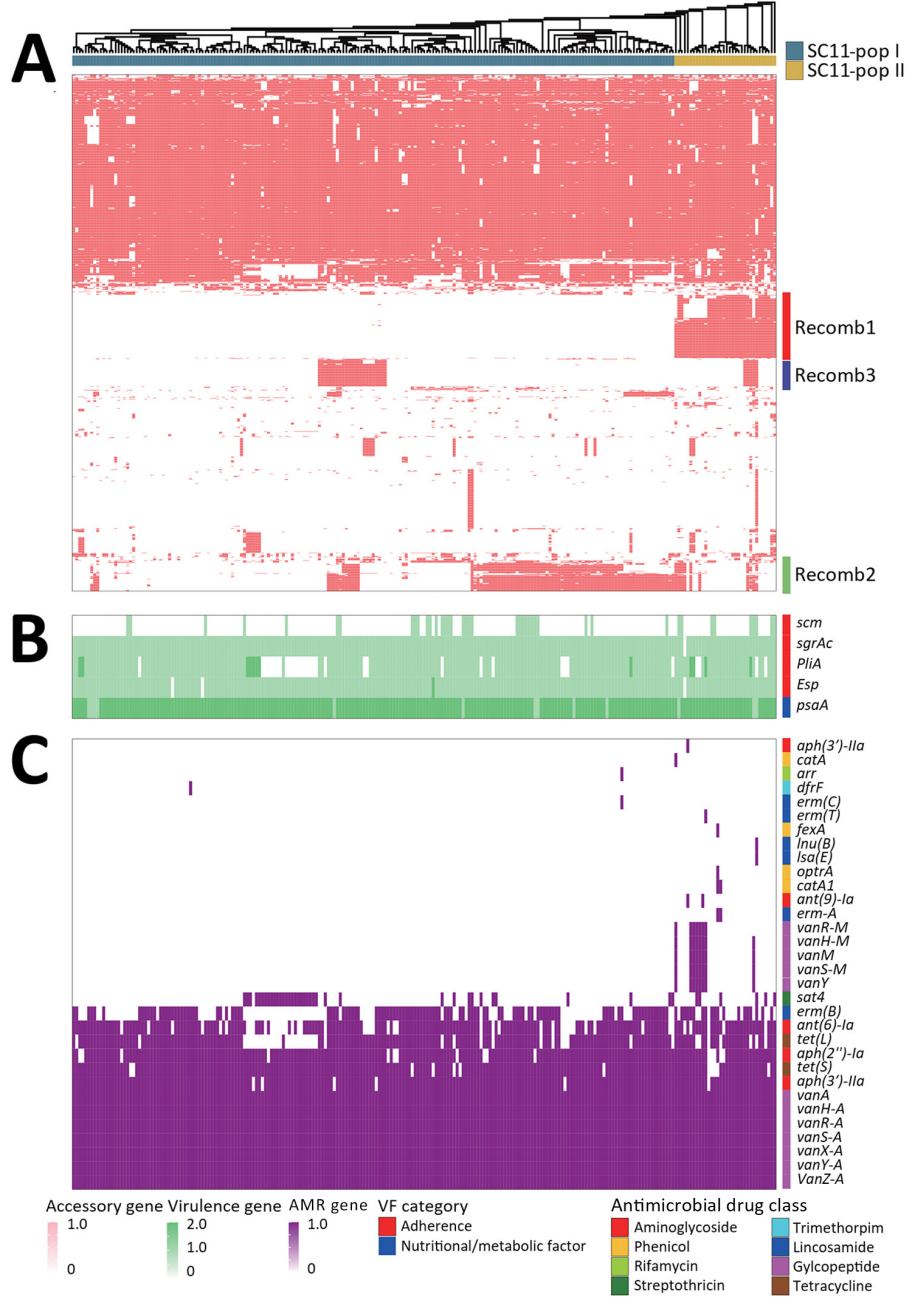
Alignment of SC11 to determine divergence during rapid transmission and divergence of vancomycin-resistant *Enterococcus faecium* ST80, China. To determine the genes associated with the divergence of SC11 we aligned various SC11 genes to pangenomic phylogeny. A) Accessory genes; B) virulence genes; C) AMR genes. Gene copy numbers are displayed in a color grade. We identified 17 total virulence genes; 12 are conserved in all SC11 isolates, and thus only 5 variable virulence genes are shown. Functional categories are indicated by color on the top of the virulence gene heatmap, and drug class corresponding to each AMR gene is shown on the top of AMR gene heatmap. Compared with SC11-pop I, SC11-pop II was more active in acquiring AMR genes against various antibiotic drugs, including sporadic acquisition of aminoglycoside resistance genes *ant9Ia* and *aph3IIIa*; rifamycin resistance gene *arr*; trimethoprim resistance gene *dfrF*; lincosamide resistance genes *ermA*, *ermC*, *ermT*, *lnuB*, and *lsaE*; and phenicol resistance genes *fexA*, *optrA*, *catA1*, and *catA*. AMR, antimicrobial resistance; pop, population; ARG, antimicrobial resistance gene; SC, sequence cluster; ST, sequence type.

SC11-pop I and SC11-pop II circulated in parallel for >3 years (2021–2024), and SC11-pop II showed broader transmission (Figure 2, panel B) and maintained ≈15% prevalence (Figure 2, panel C). SC11-pop II showed higher prevalence than SC11-pop I in Shenzhen and other provinces (Figure 2, panel B). Genetically, SC11-pop II exhibited enhanced horizontal gene transfer activity, carrying more insertion sequences (ISs) (Appendix 1 Figure 5), plasmid-like elements (Appendix 1 Figure 6), and diverse antimicrobial resistance genes (Figure 3). Although all SC11 isolates harbored the *VanA* operon, 8 SC11-pop II strains uniquely acquired *VanM* operon (Figure 3). SC11-pop II cases showed trends of increased hospitalization and underlying conditions, including hypertension and cardiovascular, respiratory, and kidney diseases, but statistical significance was limited by the sample size (Appendix 1 Table 5). Expanded surveillance is required to clarify clinical distinctions between SC11-pop I and SC11-pop II.

To identify divergence drivers, we compared core genomic mutations and accessory gene variations between SC11-pop I and SC11-pop II. Unexpectedly, no substantial cgSNP differences emerged (Appendix 3), indicating divergence was not driven by core genome mutations. The SC11 lineage pangenome (n = 235) comprised 3,674 genes, including 2,367 core genes and 1,307 accessory genes, an accessory-to-core gene ratio of 0.55. Concerning gene gain or loss, SC11-pop II specifically acquired 152 genes (forming Recomb1 modular) and showed higher frequency of 13 other functional unknown genes (except endoribonuclease *Pem*K) and lower frequency of 9 genes versus SC11-pop I (p<0.05) (Appendix 1 Table 6), indicating greater exogenous gene acquisition. As was observed in the hierarchical clustering of accessory genes on the pangenomic phylogeny, we identified 3 recombination gene modules (Recomb1–3) that were frequently acquired in SC11 (Figure 3, panel A). Recomb1 was exclusive to SC11-pop II (31/35 strains; 1 strain carries more than half of Recomb1 genes and is recognized as Recomb1 positive, similar to the standard for recognizing positivity in Recomb2 and Recomb3), whereas Recomb2 (68 strains) and Recomb3 (23 strains) occurred in both populations (Figure 3, panel A). The fixation of Recomb1–3 suggested their roles in emergence and adaptation of novel variants in SC11. Of note, Recomb1 contained 11 carbohydrate metabolism genes (Appendix 1 Table 4), which are known factors in *E. faecium* that contribute to clinical adaption and epidemics of *E. faecium* (*12*). The stable 3-year persistence of Recomb1 in SC11-pop II across regions suggests a functional importance in host adaptation and potential virulence.

Recombination surpasses mutation as the primary driver of *E. faecium* genetic diversity (*11*), and IS-mediated events occur within days during infection (*13*). Ten families of IS elements were found in all SC11 isolates (Appendix 1 Figure 5**)**. Enhanced IS transposition was associated with rapid core gene mutation (Appendix 1). Recomb1–3 acquisitions were linked to IS-mediated recombination, primarily involving genes related to DNA transposition, replication, or recombination (Appendix 1 Table 4). Recomb1 contained more ISs than Recomb2 or Recomb3, and IS91 was exclusively acquired by SC11-pop II. High-frequency modular recombination in Recomb1 involved IS91, ISL3, and IS256 (specific to SC11-pop II), whereas IS200/IS605 and IS1182 occurred at lower frequencies (Table; Appendix 1 Figure 5). Recomb2 in SC11-pop I occasionally incorporated IS3 alongside ISL3, IS66, IS982, IS256, or IS30 (Table). Recomb3 exclusively associated with IS3 in all 7 SC11-pop II isolates but was absent in SC11-pop I (Table). No plasmid marker genes co-occurred with Recomb1–3, except 9 Recomb1 genes colocalized with MOBT (plasmid relaxase) on contig AXARS010000069.1 (strain SZYSC_22VRE31), suggesting that plasmids did not directly transmit Recomb1–3.

**Table:** Distribution of insertion sequences associated with 3 recombination regions during rapid transmission and divergence of vancomycin-resistant *Enterococcus faecium* sequence type 80, China*

Region	SC11	IS+ sample	IS91	ISL3	IS1182	IS200/IS605	IS256	IS3	IS66	IS982	IS30	
Recomb1	SC11-pop I	0	0	0	0	0	0	0	0	0	0	
	SC11-pop II	31	24†	23	1	3	11	0	0	0	0	
	Proportion of SC11-pop II‡	31/35	24/35‡	23/35	1/35	3/35	11/35	0	0	0	0	
Recomb2	SC11-pop I	80	0	4	0	0	1	81	3	2	1	
	SC11-pop II	8	0	0	0	0	0	8	0	0	0	
	Proportion of SC11-pop I	80/200	0	2/200	0	0	1/200	78/200	3/200	2/200	1/200	
	Proportion of SC11-pop II	8/35	0	0/35	0	0	0/35	8/35	0/35	0/35	0/35	
Recomb3	SC11-pop I	0	0	0	0	0	0	0	0	0	0	
	SC11-pop II	7	0	0	0	0	0	7	0	0	0	
	Proportion of SC11-pop II	7/35	0	0	0	0	0	7/35	0	0	0	

*SC11-pop I, n = 200 strains; SC11-pop II, n = 35 strains. For identifying the associated IS, in certain Recomb modulars, the upstream (5 kb) and downstream (5 kb) of each Recomb component gene were searched using ISEscan v1.7.2.3 (*14*). IS, insertion sequence; pop, population; SC11, sequence cluster 11; +, positive. †Total IS number can be identified. ‡The frequency of isolates carrying the corresponding IS.

## Conclusions

Identifying SC11’s most recent ancestor is crucial for elucidating its evolutionary mechanism and mitigating emergent threats. We hypothesize a shared ancestry between SC11-root and SC11-outbreak sublineages. Expanded surveillance of outbreak-associated hospitals and retrospective analysis of pre-2021 VREF isolates are needed to trace the origin.

In summary, we showed that increasing VREF prevalence in Shenzhen, China, constitutes part of the ongoing SC11 outbreak, likely originating from Guangzhou. Population structure analysis revealed 2 stable, circulating SC11 subpopulations, emergence of which was driven by IS-mediated recombination. Sustained surveillance of those subpopulations is essential to prevent the emergence of high-risk clones with increased transmissibility and virulence.

Appendix 1 Additional information for rapid transmission and divergence of ancomycin-resistant *Enterococcus faecium* sequence type 80, China

Appendix 2 Demographic and clinical information of patients infected by VREF for rapid transmission and divergence of vancomycin-resistant *Enterococcus faecium* sequence type 80, China.

Appendix 3 Core gene SNP comparison among SC11-pop I and SC11-pop II for rapid transmission and divergence of vancomycin-resistant *Enterococcus faecium* sequence type 80, China.
