# Supplementary material for: Rapid Transmission and Divergence of Vancomycin-Resistant Enterococcus faecium Sequence Type 80, China
Source: Emerg Infect Dis. 2025 May;31(5):1000–5. doi: 10.3201/eid3105.241649 (PMC12044253; doi:10.3201/eid3105.241649)
Supplement: Appendix 1 — Additional information for rapid transmission and divergence of ancomycin-resistant Enterococcus faecium sequence type 80, China [file 24-1649-Techapp-s1.pdf]

Article DOI: https://doi.org/10.3201/eid3105.241649

EID cannot ensure accessibility for supplementary materials supplied by authors. Readers who have difficulty accessing supplementary content should contact the authors for assistance.

Rapid Transmission and Divergence of Vancomycin-Resistant *Enterococcus faecium* Sequence Type 80, China

Appendix 1

Supplemental Materials and Methods

VREF Isolates and Antimicrobials Susceptibility Testing

VREF isolating history, strains, and their clinical information since 2018 were retrieved from 7 hospitals in Shenzhen, named SZ_A-F in this study for convenience and confidential. Bacterial identification was performed by Vitek 2-compact. Vancomycin resistance was determined by disk diffusion test or the E-test method. The susceptibility breakpoint was interpreted as recommended by Clinical and Laboratory Standards Institute (CLSI) 2023 M100 (*1*).

Whole-Genome Sequencing, Assembly, Annotation, and Global Phylogenetic Inference

Genomic DNA extraction and WGS were done by Illumina platforms with PE150 strategy and 1-Gb bases per sample. After quality control of the raw reads using fastp (*2*), clean reads were assembled into contigs by Shovill (https://github.com/tseemann/shovill), which uses SPAdes (*3*) as its core. The completeness and contamination of assembled genomes were assessed using checkM (*4*). ST type identification was done by using srst2 (*5*). Genome annotation was done using prokka (*6*). *E. faecium* strain V24 (CP036151.1) (*7*) was used as the reference genome to identify core genomic single nucleotide polymorphisms (cgSNPs) using

Snippy (*8*) with default parameters and removing the recombination regions. Phylogenetic trees were constructed based cgSNP matrix using maximum likelihood algorithm and visualized by ggtree package in R (*9*).

Transmission Inference

Transmission relationships were inferred from pairwise SNP distance matrices combined with isolate metadata, including ST types, source information, isolation dates, and ward distributions, using the Seqtrack algorithm (*10*) incorporated into the GraphSNP platform (*11*). For visualization in GraphSNP, the following settings were applied: under "Adjust Graph Settings," parameters such as analysis type ("Cluster"), method ("Threshold-based Minimum Spanning Tree"), and layout ("Cose Bilkent-Compound") were selected. Furthermore, nodes were colored based on "hospital" to represent different sources of isolates.

AMR Gene, Virulence Gene, Insertion Sequence, and Plasmid-Like Elements Prediction

Virulence genes and antibiotic resistance genes were identified from genome assemblies using ABRicate v1.0.1 (https://github.com/tseemann/abricate) against the VFDB database (*12*) and AMRFinderPlus v3.11.14 (*13*), respectively. For comprehensive detection of virulence genes, ABRicate was combined with blastp v2.15.0, using a minimum coverage threshold of 80% and a similarity cutoff of 80%. Plasmid-like elements were predicted using MOB-suite v1.4.9 (*14*) and PlasmidFinder v4.0.0 (*15*), with the union of their results to ensure accurate prediction. Insertion sequences (ISs) were identified by ISEscan v1.7.2.3 (*16*) with an e-value threshold set to $<e{-}10$ for stringent identification.

Pan Genome and Population Structure Analysis

The pangenome was identified using Panaroo v2.5.1 (*17*) to differentiate core genomes, accessory genomes, cloud genes, and shell genes. Phylogenetic trees were inferred through Roary 1.7.0 (*18*) based on presence-absence matrices and visualized interactively with MicroReAct (https://microreact.org/upload) (*19*). To analyze population structure, we used

PopPUNK v2.0.0 (*20*), a software tool that estimates genetic distances by comparing bacterial core and accessory genomes. To determine the optimal number of clusters, we applied a Bayesian Gaussian mixture model (BGMM) with cross-validation, testing K values from 1 to 10. Based on optimization for convergence and result accuracy, we selected K = 3 as the final clustering parameter.

Supplemental Results and Discussion

Clinical Characterization of VREF Infections in 7 Shenzhen Hospitals

Forty-two VREF isolates were recovered from 39 patients of 7 hospitals, all of which are isolated after April 2022, except VRE_TPH_005 isolated in June 2020. The average age of these patients was 54.6 years old, with 30- to 60-year-old patients accounting for 86%. The average hospitalization was 57 days (Table). Although the rate improves and cure rate of patients is up to 51%, the mortality rate is as high as 12%, suggesting VREF may aggravate the diseases endured by patients.

Two wards, critical care medicine and oncology, are of high prevalence, 22% and 20% patients, respectively; the other 2 wards, hepatitis and cirrhosis, and urology each contributed 6%. Although the other 16 wards sporadically reported VREF infections (Appendix 1 Figure 1, panel C). The main associated diseases were respiratory diseases (38%), hypertension (36%), hematologic diseases (33%), diabetes (26%), and liver diseases (24%) (Table). According to the original sample types, urine tract was the dominant infection site (52%) and then followed by blood (10%) and ascites (7%), suggesting more invasive infections occurred (Appendix 1 Figure 1, panel D).

Emergence of ST80_GDvariant1 Was Independent and Convergent Events

Unexpectedly, the ST80_GDvariant1 isolates were scattered on the SC11-outbreank_lineage not only on the cgSNP phylogeny (Appendix 1 Figures 1 and 2, panel A) but

also on the pangenomic phylogeny (Figure 3, panel A). Meanwhile, these isolates were from 3 different hospitals across 3 years, suggesting that the emergence of the ST80_GDvariant1 was an independent and convergent event (Appendix 1 Figure 2, panel C), and the *ddl* locus, mutated from 1 to 194, might be of high tendency. The *ddl* locus was reported to be linked to recombinational exchanges at *pbp2b* loci in penicillin-resistant *Streptococcus pneumoniae* isolates (*21*) stressed by penicillin.

Inter-Region/Hospital and Nosocomial Transmission

Transmissions analysis revealed that SC11 are separately transmitted in Shenzhen and Guangzhou and other cities in Guangdong (Appendix 1 Figure 3, panel A). Given the higher diversity in Guangzhou (co-circulation of SC11-root and SC11-outbreak sublineages), the outbreak in Shenzhen likely originated in Guangzhou through limited cross-city transmission. Both nosocomial transmission and community- or cross-hospital–acquired transmission were observed in Shenzhen (Appendix 1 Table 1; Appendix 1 Figure 3, panel B). Because ancestral strains are isolated in Guangzhou, the SC11 VREF outbreak in Shenzhen is suggested to be imported from Guangzhou through limited cross-city transmission. cgSNP distances between each transmission pair are mainly 3–5, whereas that of 2 pairs are 6 and 7. The other 3 pairs, including the 2 ancestral strains and 1 rapid mutation strain, show SNP distances more than 20 (Appendix 1 Figure 3, panel A). The average cgSNP distances of transmission pairs in Shenzhen, 4.00, is larger than that in Guangzhou and other cities in Guangdong Province 3.47, suggesting SC-11 might mutate faster in Shenzhen during transmission.

Among the 39 VREF-infected patients, 34 were a potentially nosocomial infection that are identified after 48 hours of admission, whereas 5 were identified within 48 hours after admission, suggesting they were infected from the community or another hospital. Nosocomial transmission was revealed in 2 hospitals, with 2 transmission groups in TPH lasting at least 30 days and 265 days, respectively, and 1 transmission cluster in SZCMH lasting at least 60 days within the ICU (Appendix 1 Table 1; Appendix 1 Figure 3, panel B).

Although the transmission relationship among the majority of the isolates is hard to disclose through cgSNP variations (groups 1 and 5 in Appendix 1 Figure 3, panel A; group 1 in Appendix 1 Figure 3, panel B), because of no cgSNP differences among these isolates. It is necessary to optimize or develop novel methods to improve the resolution to strengthen the power of transmission inference using the WGS method.

Virulence Factors Possible Tuning SC11 Lineage

Seventeen virulence factors in total were carried by the SC11 lineage with no distribution differences among SC11-pop I and II. Adherence-related factors, especially *Scm* and *PilA*, along with the other 4 (*Esp*, *sgrAc*, *PilB*, *acm*) were frequently found with a gain or loss among the isolates. Another factor, *psaA*, played roles in nutrition, frequently changing its copy numbers (Figure 3, panel B). These factors might tune SC11 to colonize and adapt to the host or environment.

Gene Contents and Function of Recomb1–3

A total of 152 genes involving in Recomb1 were found to be distributed consecutively on the chromosome using SZYSC_23VRE019 (GCA_037477245.1) as the locating reference. Twenty-five genes can be functionally annotated using Prokka (*6*). Among them, besides the 2 IS-incorporated transposases and 1 DNA polymerase gene, 4 sugar metabolism genes (*agaS*, *bga*, *ycsE*, and *group_255*), 4 carbohydrate phosphotransferase system (PTS) genes (*levE*, *lacD2*/*lacC*, *group_275*, corresponding to fructose, lactose, and mannose, respectively), 4 amino metabolism genes (*ansB*, *iphP*, *pepC*, and *dap*), and 3 transcriptional regulator genes of amino sugar metabolism (*nagR*, *frlR*, and *bcrR*) were identified (Appendix 1 Table 5). Four sugar metabolism (*agaS*, *bga*, *ycsE*, and *group_255*) genes involved in disassembling and synthesis of various sugars, even in regulating virulence of bacteria outer membrane polysaccharide. PTS genes encode transmembrane proteins that participate in sugar intake. PTS genes are always co-located with sugar metabolism genes (*22*). PTS allows enterococci to use a broad variety of

sugars as carbohydrate sources and better adapt to changing environments and can also have a role in the general stress response, as virulence factors helping the enterococci to colonize and survive within the host, and even in biofilm formation and endocarditis development (*23*). Sugar metabolism gene *bga* (*24*) and amino metabolism gene *ansB* (*25*) were also reported in adherence to human epithelial cells and thus mediating virulence. Among amino metabolism genes, tyrosine-protein phosphatase is associated with growth and virulence of many bacteria, including regulate the biosynthesis of secreted polysaccharides (*26*). Few of those Recomb1 genes are investigated in enterococci. Carbohydrate metabolism genes (*27*) are always direct factors contributed to the clinical survival and epidemics of *E. faecium*. VREF adapted during persistent intestinal colonization and bloodstream infection, in part, through acquiring carbohydrate metabolism genes and thus the optimizing the use of distinct carbohydrates in the intestinal versus bloodstream environments (*28*). Our study highlights the necessity to investigate the precise roles of those Recomb1 genes in adaptation and pathogenesis of enterococci. A large part of the above annotated genes encode phosphatases. For their clinical importance and universally prevalence, development of drugs targeting phosphatases were developed against *Mycobacterium tuberculosis* and *Yersinia* (*29*). It would be useful to investigate the possibility in control pathogenic enterococci.

There are 65 genes in total involves Recomb2 modules. Among them, 2 energy-drive or synthetic genes, P-loop guanosine triphosphatase *YjiA* (GTpase), and ATP-dependent *Clp* protease proteolytic subunit *clpP* were annotated. The former participate in critical cellular processes ranging from ribosomal protein synthesis to the cell cycle (*14*,*15*), whereas the latter, in contrary, participate in the proteolytic elimination of misfolded or aggregated proteins, as well as the formation of virulent phenotypes and in the response to different types of stress (*16*). Additionally, 1 site-specific tyrosine recombinase *XerC*, which acts by catalyzing the cutting and rejoining of the recombining DNA molecules, was identified in Recomb2. Recomb3 encompasses 59 genes. Among them, 2 genes, DNA topoisomerase III *topB2* (*17*) and

chromosome partitioning protein *Soj* (*18*,*19*), have roles in DNA replication. Another gene, RNase *ToxN*, is a core component of type III toxin-antitoxin systems and are reported to block phage development through widespread cleavage of phage transcripts, thereby preventing spread of viral particles through the bacterial population (*20*) (Appendix 1 Table 5).

IS-Mediated Recombination Events Occurred Frequently and Might Play Role in VREF Evolution

The copy numbers of IS3, IS6, ISL3, IS30, and IS256 specifically increased in Shenzhen isolates (Appendix 1 Figure 5**)**, which suggested population divergence occurred along with geographic distribution and supported the independent transmission of SC11 in Shenzhen. We found that enhanced IS element transposition was associated with rapid core gene mutation. For example, 1 rapid mutated strain, SZYSC_23VRE019 in Guangzhou, 2023, with an average of 20-SNP distance from other strains in the SC11 outbreak sublineage, is observed (Figure 1). This strain exhibited 182 ISs, representing a 2.68-fold increase over the average (68 ISs). Notably, six specific IS elements (ISL3, IS30, IS256, IS982, IS110, and IS1182) demonstrated significant copy number elevation (Appendix 1 Figure 5). It is suggested that IS transposition may drive its core genes and tune its activity and function to adapt to genomic recombination and hence environmental stress.

References

<bok>1. Clinical and Laboratory Standards Institute. Performance standards for antimicrobial susceptibility testing: 33rd informational supplement. Wayne (PA): The Institute; 2023.</bok>

<jrn>2. Chen S. Ultrafast one-pass FASTQ data preprocessing, quality control, and deduplication using fastp. iMeta. 2023;2:e107. PubMed https://doi.org/10.1002/imt2.107</jrn>

<jrn>3. Prjibelski A, Antipov D, Meleshko D, Lapidus A, Korobeynikov A. Using SPAdes de novo assembler. Curr Protoc Bioinformatics. 2020;70:e102. PubMed https://doi.org/10.1002/cpbi.102</jrn>

<jrn>4. Parks DH, Imelfort M, Skennerton CT, Hugenholtz P, Tyson GW. CheckM: assessing the quality of microbial genomes recovered from isolates, single cells, and metagenomes. Genome Res. 2015;25:1043–55. PubMed https://doi.org/10.1101/gr.186072.114</jrn>

<jrn>5. Inouye M, Dashnow H, Raven L-A, Schultz MB, Pope BJ, Tomita T, et al. SRST2: rapid genomic surveillance for public health and hospital microbiology labs. Genome Med. 2014;6:90. PubMed https://doi.org/10.1186/s13073-014-0090-6</jrn>

<jrn>6. Seemann T. Prokka: rapid prokaryotic genome annotation. Bioinformatics. 2014;30:2068–9. PubMed https://doi.org/10.1093/bioinformatics/btu153</jrn>

<jrn>7. Pinholt M, Bayliss SC, Gumpert H, Worning P, Jensen VVS, Pedersen M, et al. WGS of 1058 *Enterococcus faecium* from Copenhagen, Denmark, reveals rapid clonal expansion of vancomycin-resistant clone ST80 combined with widespread dissemination of a vanA-containing plasmid and acquisition of a heterogeneous accessory genome. J Antimicrob Chemother. 2019;74:1776–85. PubMed https://doi.org/10.1093/jac/dkz118</jrn>

<eref>8. Seemann T. Snippy: rapid haploid variant calling and core genome alignment [cited 2020 Feb 21]. https://github.com/tseemann/snippy, date of access (Feb 21, 2020) </eref>

<jrn>9. Yu G, Smith DK, Zhu H, Guan Y, Lam TT-Y. ggtree: an r package for visualization and annotation of phylogenetic trees with their covariates and other associated data. Methods Ecol Evol. 2017;8:28–36. https://doi.org/10.1111/2041-210X.12628</jrn>

<jrn>10. Jombart T, Eggo RM, Dodd PJ, Balloux F. Reconstructing disease outbreaks from genetic data: a graph approach. Heredity. 2011;106:383–90. PubMed https://doi.org/10.1038/hdy.2010.78</jrn>

<jrn>11. Permana B, Beatson SA, Forde BM. GraphSNP: an interactive distance viewer for investigating outbreaks and transmission networks using a graph approach. BMC Bioinform. 2023;24:209. PubMed https://doi.org/10.1186/s12859-023-05332-x</jrn>

<jrn>12. Liu B, Zheng D, Jin Q, Chen L, Yang J. VFDB 2019: a comparative pathogenomic platform with an interactive web interface. Nucleic Acids Res. 2019;47:D687–92. PubMed https://doi.org/10.1093/nar/gky1080</jrn>

<jrn>13. Feldgarden M, Brover V, Gonzalez-Escalona N, Frye JG, Haendiges J, Haft DH, et al. AMRFinderPlus and the Reference Gene Catalog facilitate examination of the genomic links among antimicrobial resistance, stress response, and virulence. Sci Rep. 2021;11:12728. PubMed https://doi.org/10.1038/s41598-021-91456-0</jrn>

<jrn>14. Robertson J, Nash JHE. MOB-suite: software tools for clustering, reconstruction and typing of plasmids from draft assemblies. Microb Genom. 2018;4:e000206. PubMed https://doi.org/10.1099/mgen.0.000206</jrn>

<jrn>15. Carattoli A, Hasman H. PlasmidFinder and in silico pMLST: identification and typing of plasmid replicons in whole-genome sequencing (WGS). Methods Mol Biol. 2020;2075:285–94. PubMed https://doi.org/10.1007/978-1-4939-9877-7_20</jrn>

<jrn>16. Xie Z, Tang H. ISEScan: automated identification of insertion sequence elements in prokaryotic genomes. Bioinformatics. 2017;33:3340–7. PubMed https://doi.org/10.1093/bioinformatics/btx433</jrn>

<jrn>17. Tonkin-Hill G, MacAlasdair N, Ruis C, Weimann A, Horesh G, Lees JA, et al. Producing polished prokaryotic pangenomes with the Panaroo pipeline. Genome Biol. 2020;21:180. PubMed https://doi.org/10.1186/s13059-020-02090-4</jrn>

<jrn>18. Page AJ, Cummins CA, Hunt M, Wong VK, Reuter S, Holden MTG, et al. Roary: rapid large-scale prokaryote pan genome analysis. Bioinformatics. 2015;31:3691–3. PubMed https://doi.org/10.1093/bioinformatics/btv421</jrn>

<jrn>19. Argimón S, Abudahab K, Goater RJE, Fedosejev A, Bhai J, Glasner C, et al. Microreact: visualizing and sharing data for genomic epidemiology and phylogeography. Microb Genom. 2016;2:e000093. PubMed https://doi.org/10.1099/mgen.0.000093</jrn>

<jrn>20. Lees JA, Harris SR, Tonkin-Hill G, Gladstone RA, Lo SW, Weiser JN, et al. Fast and flexible bacterial genomic epidemiology with PopPUNK. Genome Res. 2019;29:304–16. PubMed https://doi.org/10.1101/gr.241455.118</jrn>

<jrn>21. Enright MC, Spratt BG. Extensive variation in the ddl gene of penicillin-resistant *Streptococcus pneumoniae* results from a hitchhiking effect driven by the penicillin-binding protein 2b gene. Mol Biol Evol. 1999;16:1687–95. PubMed https://doi.org/10.1093/oxfordjournals.molbev.a026082</jrn>

<jrn>22. Deutscher J, Aké FM, Derkaoui M, Zébré AC, Cao TN, Bouraoui H, et al. The bacterial phosphoenolpyruvate:carbohydrate phosphotransferase system: regulation by protein phosphorylation and phosphorylation-dependent protein-protein interactions. Microbiol Mol Biol Rev. 2014;78:231–56. PubMed https://doi.org/10.1128/MMBR.00001-14</jrn>

<jrn>23. Gao W, Howden BP, Stinear TP. Evolution of virulence in *Enterococcus faecium*, a hospital-adapted opportunistic pathogen. Curr Opin Microbiol. 2018;41:76–82. PubMed https://doi.org/10.1016/j.mib.2017.11.030</jrn>

<jrn>24. Limoli DH, Sladek JA, Fuller LA, Singh AK, King SJ. BgaA acts as an adhesin to mediate attachment of some pneumococcal strains to human epithelial cells. Microbiology (Reading). 2011;157:2369–81. PubMed https://doi.org/10.1099/mic.0.045609-0</jrn>

<jrn>25. George DT, Mathesius U, Behm CA, Verma NK. The periplasmic enzyme, AnsB, of *Shigella flexneri* modulates bacterial adherence to host epithelial cells. PLoS One. 2014;9:e94954. PubMed https://doi.org/10.1371/journal.pone.0094954</jrn>

<jrn>26. Standish AJ, Morona R. The role of bacterial protein tyrosine phosphatases in the regulation of the biosynthesis of secreted polysaccharides. Antioxid Redox Signal. 2014;20:2274–89. PubMed https://doi.org/10.1089/ars.2013.5726</jrn>

<edb>27. Palmer KL, Schaik WV, Willems RJL, Gilmore MS. Enterococcal genomics. In: Gilmore MS, Clewell DB, Ike Y, Shankar N, editors. Enterococci: from commensals to leading causes of drug resistant infection. Boston: Massachusetts Eye and Ear Infirmary; 2014. p. 193–230.</edb>

<jrn>28. Chilambi GS, Nordstrom HR, Evans DR, Ferrolino JA, Hayden RT, Marón GM, et al. Evolution of vancomycin-resistant *Enterococcus faecium* during colonization and infection in immunocompromised pediatric patients. Proc Natl Acad Sci U S A. 2020;117:11703–14. PubMed https://doi.org/10.1073/pnas.1917130117</jrn>

<jrn>29. Sajid A, Arora G, Singhal A, Kalia VC, Singh Y. Protein phosphatases of pathogenic bacteria: role in physiology and virulence. Ann Rev Microbiol.. 2015;69:527–47. PubMed https://doi.org/10.1146/annurev-micro-020415-111342.</jrn>

Appendix 1 Table 1. Demographic and clinical characteristics of patients with VRE infections

Characteristics	n (%)
Total	42 (100%)
Age, y	Average, 54.59
≤30	3 (7%)
30–60	36 (86%)
≥60	3 (7%)
Sex	
M	22 (52%)
F	20 (48%)
Average days of hospitalization	57 (2–288 d)
Diseases	
Respiratory diseases	16 (38%)
Hypertension	15 (36%)
Hematologic diseases	14 (33%)
Diabetes	11 (26%)
Liver diseases	10 (24%)
Urinary system diseases	8 (19%)
Kidney diseases	7 (17%)
Malignant tumor	5 (12%)
Cardiovascular diseases	5 (12%)
Infectious diseases	3 (7%)
Gastrointestinal diseases	3 (7%)

Characteristics	n (%)
HIV	1 (2%)
Active tuberculosis	1 (2%)
Rheumatism	1 (2%)
Fractures	1 (2%)
Kidney transplant	1 (2%)
Prognosis	
Improvement	13 (30%)
Cure	9 (21%)
Death	5 (12%)
Not cured	3 (7%)
Unknown	12 (28%)

Appendix 1 Table 2. ST80 isolates of public database used for genomic analysis in this study*

Strain	Source	Year	Geographic group	Country	Host	BRC ID or GenBank accession no.
*E.faecium*_E0333	Blood	1997	Middle East	Israel	Human	1138884.3
*E.faecium*_EnGen0305	Human	1994	Europe	France	NA	1158641.3
*E.faecium*_strain_10733_6#20	Human invasive infection	NA	NA	NA	NA	1352.10003
*E.faecium*_strain_10733_3#58	Human invasive infection	NA	NA	NA	NA	1352.10071
*E.faecium*_strain_16404_2#32	Wastewater	NA	NA	NA	NA	1352.10279
*E.faecium*_strain_16404_2#35	Wastewater	NA	NA	NA	NA	1352.10283
*E.faecium*_strain_16880_8#37	Wastewater	NA	NA	NA	NA	1352.10297
*E.faecium*_strain_16880_8#36	Wastewater	NA	NA	NA	NA	1352.10299
*E.faecium*_strain_16404_1#64	Wastewater	NA	NA	NA	NA	1352.10327
*E.faecium*_strain_16404_1#77	Wastewater	NA	NA	NA	NA	1352.10328
*E.faecium*_strain_16404_1#76	Wastewater	NA	NA	NA	NA	1352.10329
*E.faecium*_strain_16404_1#78	Wastewater	NA	NA	NA	NA	1352.10331
*E.faecium*_strain_16356_2#79	Wastewater	NA	NA	NA	NA	1352.10333
*E.faecium*_strain_17138_5#93	Wastewater	NA	NA	NA	NA	1352.10337
*E.faecium*_strain_16880_8#29	Wastewater	NA	NA	NA	NA	1352.10338
*E.faecium*_strain_16404_1#82	Wastewater	NA	NA	NA	NA	1352.10339
*E.faecium*_strain_16356_2#83	Wastewater	NA	NA	NA	NA	1352.10343
*E.faecium*_strain_16356_2#85	Wastewater	NA	NA	NA	NA	1352.10348

Strain	Source	Year	Geographic group	Country	Host	BRC ID or GenBank accession no.
*E.faecium*_strain_16356_2#84	Wastewater	NA	NA	NA	NA	1352.10349
*E.faecium*_strain_16404_2#10	Wastewater	NA	NA	NA	NA	1352.10363
*E.faecium*_strain_16404_2#11	Wastewater	NA	NA	NA	NA	1352.10364
*E.faecium*_strain_16404_2#52	Wastewater	NA	NA	NA	NA	1352.10365
*E.faecium*_strain_16404_2#5	Wastewater	NA	NA	NA	NA	1352.10366
*E.faecium*_strain_16404_2#7	Wastewater	NA	NA	NA	NA	1352.10367
*E.faecium*_strain_16404_2#6	Wastewater	NA	NA	NA	NA	1352.10368
*E.faecium*_strain_16404_2#8	Wastewater	NA	NA	NA	NA	1352.10369
*E.faecium*_strain_16880_8#20	Wastewater	NA	NA	NA	NA	1352.10372
*E.faecium*_strain_16404_2#9	Wastewater	NA	NA	NA	NA	1352.10373
*E.faecium*_strain_16404_1#13	Wastewater	NA	NA	NA	NA	1352.10375
*E.faecium*_strain_16404_1#80	Wastewater	NA	NA	NA	NA	1352.10381
*E.faecium*_strain_16404_1#40	Wastewater	NA	NA	NA	NA	1352.10382
*E.faecium*_strain_16404_1#57	Wastewater	NA	NA	NA	NA	1352.10383
*E.faecium*_strain_16404_1#65	Wastewater	NA	NA	NA	NA	1352.10384
*E.faecium*_strain_16404_1#15	Wastewater	NA	NA	NA	NA	1352.10387
*E.faecium*_strain_16880_8#31	Wastewater	NA	NA	NA	NA	1352.10391
*E.faecium*_strain_16404_1#24	Wastewater	NA	NA	NA	NA	1352.10399
*E.faecium*_strain_16404_2#19	Wastewater	NA	NA	NA	NA	1352.10402
*E.faecium*_strain_16404_1#23	Wastewater	NA	NA	NA	NA	1352.10404
*E.faecium*_strain_16404_1#22	Wastewater	NA	NA	NA	NA	1352.10405
*E.faecium*_strain_16404_2#22	Wastewater	NA	NA	NA	NA	1352.10421
*E.faecium*_strain_16404_2#23	Wastewater	NA	NA	NA	NA	1352.10423
*E.faecium*_strain_16404_2#24	Wastewater	NA	NA	NA	NA	1352.10426
*E.faecium*_strain_16404_1#48	Wastewater	NA	NA	NA	NA	1352.10431
*E.faecium*_strain_16404_2#27	Wastewater	NA	NA	NA	NA	1352.10433
*E.faecium*_strain_16404_2#44	Wastewater	NA	NA	NA	NA	1352.10441
*E.faecium*_strain_16404_2#16	Wastewater	NA	NA	NA	NA	1352.10444
*E.faecium*_strain_16404_2#15	Wastewater	NA	NA	NA	NA	1352.10447
*E.faecium*_strain_16404_2#56	Wastewater	NA	NA	NA	NA	1352.10448
*E.faecium*_strain_16880_8#12	Wastewater	NA	NA	NA	NA	1352.10453
*E.faecium*_strain_16404_2#60	Wastewater	NA	NA	NA	NA	1352.10454
*E.faecium*_strain_16404_2#65	Wastewater	NA	NA	NA	NA	1352.10461
*E.faecium*_strain_16404_2#69	Wastewater	NA	NA	NA	NA	1352.10469
*E.faecium*_strain_16404_2#86	Wastewater	NA	NA	NA	NA	1352.10479
*E.faecium*_strain_16880_8#7	Wastewater	NA	NA	NA	NA	1352.10488

Strain	Source	Year	Geographic group	Country	Host	BRC ID or GenBank accession no.
*E.faecium*_strain_16880_8#4	Wastewater	NA	NA	NA	NA	1352.10491
*E.faecium*_strain_16880_8#5	Wastewater	NA	NA	NA	NA	1352.10492
*E.faecium*_strain_16880_8#15	Wastewater	NA	NA	NA	NA	1352.10509
*E.faecium*_strain_16880_8#13	Wastewater	NA	NA	NA	NA	1352.10514
*E.faecium*_strain_16880_8#17	Wastewater	NA	NA	NA	NA	1352.10518
*E.faecium*_strain_16880_8#22	Wastewater	NA	NA	NA	NA	1352.10522
*E.faecium*_strain_16880_8#24	Wastewater	NA	NA	NA	NA	1352.10523
*E.faecium*_strain_16880_8#26	Wastewater	NA	NA	NA	NA	1352.10525
*E.faecium*_strain_16880_8#25	Wastewater	NA	NA	NA	NA	1352.10526
*E.faecium*_strain_16880_8#27	Wastewater	NA	NA	NA	NA	1352.10528
*E.faecium*_strain_16356_1#39	Wastewater	NA	NA	NA	NA	1352.10536
*E.faecium*_strain_16356_1#61	Wastewater	NA	NA	NA	NA	1352.10546
*E.faecium*_strain_16356_1#60	Wastewater	NA	NA	NA	NA	1352.10549
*E.faecium*_strain_16356_1#59	Wastewater	NA	NA	NA	NA	1352.1055
*E.faecium*_strain_16356_2#11	Wastewater	NA	NA	NA	NA	1352.10565
*E.faecium*_strain_16356_2#13	Wastewater	NA	NA	NA	NA	1352.10567
*E.faecium*_strain_16356_1#88	Wastewater	NA	NA	NA	NA	1352.10569
*E.faecium*_strain_16356_2#10	Wastewater	NA	NA	NA	NA	1352.10576
*E.faecium*_strain_16356_1#46	Wastewater	NA	NA	NA	NA	1352.10577
*E.faecium*_strain_16356_1#47	Wastewater	NA	NA	NA	NA	1352.10578
*E.faecium*_strain_16356_1#72	Wastewater	NA	NA	NA	NA	1352.10579
*E.faecium*_strain_16356_1#71	Wastewater	NA	NA	NA	NA	1352.1057
*E.faecium*_strain_16356_1#73	Wastewater	NA	NA	NA	NA	1352.10582
*E.faecium*_strain_16356_1#53	Wastewater	NA	NA	NA	NA	1352.10588
*E.faecium*_strain_16356_2#6	Wastewater	NA	NA	NA	NA	1352.10589
*E.faecium*_strain_16356_2#9	Wastewater	NA	NA	NA	NA	1352.1058
*E.faecium*_strain_16356_2#28	Wastewater	NA	NA	NA	NA	1352.10593
*E.faecium*_strain_16356_2#15	Wastewater	NA	NA	NA	NA	1352.10596
*E.faecium*_strain_16356_2#1	Wastewater	NA	NA	NA	NA	1352.10597
*E.faecium*_strain_16356_2#14	Wastewater	NA	NA	NA	NA	1352.10598
*E.faecium*_strain_16356_2#25	Wastewater	NA	NA	NA	NA	1352.10599
*E.faecium*_strain_16356_2#21	Wastewater	NA	NA	NA	NA	1352.10601
*E.faecium*_strain_16356_2#2	Wastewater	NA	NA	NA	NA	1352.10602
*E.faecium*_strain_16356_2#19	Wastewater	NA	NA	NA	NA	1352.10603
*E.faecium*_strain_16356_2#18	Wastewater	NA	NA	NA	NA	1352.10605
*E.faecium*_strain_16356_2#3	Wastewater	NA	NA	NA	NA	1352.10613

Strain	Source	Year	Geographic group	Country	Host	BRC ID or GenBank accession no.
*E.faecium*_strain_16356_2#7	Wastewater	NA	NA	NA	NA	1352.10614
*E.faecium*_strain_16404_1#62	Wastewater	NA	NA	NA	NA	1352.10622
*E.faecium*_strain_16404_1#66	Wastewater	NA	NA	NA	NA	1352.10644
*E.faecium*_strain_16404_1#63	Wastewater	NA	NA	NA	NA	1352.10645
*E.faecium*_strain_16404_1#68	Wastewater	NA	NA	NA	NA	1352.10647
*E.faecium*_strain_17138_5#91	Wastewater	NA	NA	NA	NA	1352.10649
*E.faecium*_strain_16404_1#70	Wastewater	NA	NA	NA	NA	1352.10652
*E.faecium*_strain_16880_8#43	Wastewater	NA	NA	NA	NA	1352.10654
*E.faecium*_strain_16404_1#71	Wastewater	NA	NA	NA	NA	1352.10656
*E.faecium*_strain_16880_8#44	Wastewater	NA	NA	NA	NA	1352.10657
*E.faecium*_strain_16404_1#74	Wastewater	NA	NA	NA	NA	1352.10658
*E.faecium*_strain_16880_8#32	Wastewater	NA	NA	NA	NA	1352.10659
*E.faecium*_strain_17138_5#92	Wastewater	NA	NA	NA	NA	1352.10661
*E.faecium*_strain_ME3	Water sample from river near old railway bridge	2013	Asia	India	NA	1352.10904
*E.faecium*_strain_BA17124	Blood	2018	Asia	India	Human	1352.11045
*E.faecium*_strain_BA17063	Blood	2019	Asia	India	Human	1352.11046
*E.faecium*_strain_BA7523	Blood	2018	Asia	India	Human	1352.11047
*E.faecium*_strain_BP5067	Blood	2017	Asia	India	Human	1352.11048
*E.faecium*_strain_BP3378	Blood	2019	Asia	India	Human	1352.11049
*E.faecium*_strain_BA12993	Blood	2018	Asia	India	Human	1352.11051
*E.faecium*_strain_BP657	Blood	2017	Asia	India	Human	1352.11053
*E.faecium*_strain_A976	Blood	2019	Asia	India	Human	1352.11054
*E.faecium*_strain_A7214	Blood	2019	Asia	India	Human	1352.11055
*E.faecium*_strain_A710	Blood	2019	Asia	India	Human	1352.11056
*E.faecium*_strain_A6521	Blood	2019	Asia	India	Human	1352.11057
*E.faecium*_strain_A11051	Blood	2019	Asia	India	Human	1352.11058
*E.faecium*_strain_A13828	Blood	2019	Asia	India	Human	1352.11059
*E.faecium*_strain_A3895	Blood	2019	Asia	India	Human	1352.11061
*E.faecium*_strain_A4694	Blood	2019	Asia	India	Human	1352.11062
*E.faecium*_strain_A10290	Blood	2019	Asia	India	Human	1352.11063
*E.faecium*_strain_BSAC_ec1067	Human	2005	Europe	United Kingdom	Human	1352.11273
*E.faecium*_strain_BSAC_ec1388	Human	2006	Europe	United Kingdom	Human	1352.11345

Strain	Source	Year	Geographic group	Country	Host	BRC ID or GenBank accession no.
*E.faecium*_strain_BSAC_ec1626	Human	2007	Europe	United Kingdom	Human	1352.11388
*E.faecium*_strain_BSAC_ec1629	Human	2007	Europe	United Kingdom	Human	1352.11393
*E.faecium*_strain_BSAC_ec1715	Human	2007	Europe	United Kingdom	Human	1352.11412
*E.faecium*_strain_BSAC_ec2611	Human	2011	Europe	United Kingdom	Human	1352.11569
*E.faecium*_strain_BSAC_ec2641	Human	2011	Europe	United Kingdom	Human	1352.11575
*E.faecium*_strain_BSAC_ec2708	Human	2011	Europe	United Kingdom	Human	1352.11591
*E.faecium*_strain_BSAC_ec2761	Human	2011	Europe	United Kingdom	Human	1352.11603
*E.faecium*_strain_BSAC_ec2755	Human	2011	Europe	United Kingdom	Human	1352.11604
*E.faecium*_strain_BSAC_ec871	Human	2004	Europe	United Kingdom	Human	1352.11703
*E.faecium*_strain_EC0165	Human	2010	Europe	United Kingdom	Human	1352.11829
*E.faecium*_strain_EC0173	Human	2010	Europe	United Kingdom	Human	1352.11845
*E.faecium*_strain_EC0218	Human	2012	Europe	United Kingdom	Human	1352.11875
*E.faecium*_strain_EC0239	Human	2012	Europe	United Kingdom	Human	1352.11889
*E.faecium*_strain_EC0248	Human	2011	Europe	United Kingdom	Human	1352.11903
*E.faecium*_strain_EC0400	Human	2011	Europe	United Kingdom	Human	1352.11948
*E.faecium*_strain_EC0493	Human	2012	Europe	United Kingdom	Human	1352.11987
*E.faecium*_strain_EC0500	Human	2012	Europe	United Kingdom	Human	1352.12002
*E.faecium*_strain_VRE_56	Human	2017	Asia	Lebanon	Human	1352.12047
*E.faecium*_strain_VRE_53	Human	2017	Asia	Lebanon	Human	1352.12048

Strain	Source	Year	Geographic group	Country	Host	BRC ID or GenBank accession no.
*E.faecium*_strain_VRE_51	Human	2017	Asia	Lebanon	Human	1352.12052
*E.faecium*_strain_VRE_48	Human	2017	Asia	Lebanon	Human	1352.12054
*E.faecium*_strain_VRE_47	Human	2017	Asia	Lebanon	Human	1352.12055
*E.faecium*_strain_VRE_43	Human	2017	Asia	Lebanon	Human	1352.12057
*E.faecium*_strain_VRE_46	Human	2017	Asia	Lebanon	Human	1352.12058
*E.faecium*_strain_VRE_41	Human	2018	Asia	Lebanon	Human	1352.12061
*E.faecium*_strain_VRE_39	Human	2018	Asia	Lebanon	Human	1352.12062
*E.faecium*_strain_VRE_40	Human	2018	Asia	Lebanon	Human	1352.12063
*E.faecium*_strain_VRE_38	Human	2018	Asia	Lebanon	Human	1352.12064
*E.faecium*_strain_VRE_33	Human	2018	Asia	Lebanon	Human	1352.12066
*E.faecium*_strain_VRE_36	Human	2018	Asia	Lebanon	Human	1352.12069
*E.faecium*_strain_VRE_29	Human	2018	Asia	Lebanon	Human	1352.12071
*E.faecium*_strain_VRE_27	Human	2018	Asia	Lebanon	Human	1352.12072
*E.faecium*_strain_VRE_28	Human	2018	Asia	Lebanon	Human	1352.12073
*E.faecium*_strain_VRE_31	Human	2018	Asia	Lebanon	Human	1352.12074
*E.faecium*_strain_VRE_30	Human	2018	Asia	Lebanon	Human	1352.12075
*E.faecium*_strain_VRE_25	Human	2018	Asia	Lebanon	Human	1352.12078
*E.faecium*_strain_VRE_17	Human	2018	Asia	Lebanon	Human	1352.12082
*E.faecium*_strain_VRE_4	Human	2018	Asia	Lebanon	Human	1352.12096
*E.faecium*_strain_VRE_2	Human	2018	Asia	Lebanon	Human	1352.12097
*E.faecium*_strain_VRE_3	Human	2018	Asia	Lebanon	Human	1352.12098
*E.faecium*_strain_VRE_1	Human	2018	Asia	Lebanon	Human	1352.12099
*E.faecium*_strain_VRE_6	Human	2018	Asia	Lebanon	Human	1352.12101
*E.faecium*_strain_VB3338	Blood	2019	Asia	India	Human	1352.12139
*E.faecium*_strain_VREA4	Sewage	2019	NA	NA	NA	1352.12161
*E.faecium*_strain_VRE3363	Rectal swab	2014	Oceania	Australia	Human	1352.12166
*E.faecium*_strain_VRE34919	Rectal swab	2019	North America	USA	Human	1352.12266
*E.faecium*_strain_VRE34361	Rectal swab	2018	North America	USA	Human	1352.12279
*E.faecium*_strain_VRE33715	Rectal swab	2018	North America	USA	Human	1352.12303
*E.faecium*_strain_Dallas_113	Rectal swab	2015	North America	USA	Human	1352.12327
*E.faecium*_strain_Dallas_16_2	Rectal swab	2015	North America	USA	Human	1352.12438
*E.faecium*_strain_VB13828	Blood	2020	Asia	India	Human	1352.12694
*E.faecium*_strain_NIZ171	Urine	2019	Asia	Malaysia	Human	1352.12695
*E.faecium*_strain_1MPJ203	Peadiatric - unoccupied bed	2017	Africa	South Africa	NA	1352.12697
*E.faecium*_strain_VB12993	Blood	2018	Asia	India	Human	1352.12699

Strain	Source	Year	Geographic group	Country	Host	BRC ID or GenBank accession no.
*E.faecium*_strain_VB976	Blood	2019	Asia	India	Human	1352.12701
*E.faecium*_strain_VB6521	Blood	2019	Asia	India	Human	1352.12702
*E.faecium*_strain_VB6171	Blood	2019	Asia	India	Human	1352.12703
*E.faecium*_strain_VB3378	Blood	2019	Asia	India	Human	1352.12704
*E.faecium*_strain_AUSMDU00022624	Rectal swab	2018	Oceania	Australia	Human	1352.12705
*E.faecium*_strain_AUSMDU00008257	Human	2017	Oceania	Australia	Human	1352.12713
*E.faecium*_strain_AUSMDU00034415	Human	2017	Oceania	Australia	Human	1352.12714
*E.faecium*_strain_1MPJ201	Peadiatric - unoccupied bed	2017	Africa	South Africa	NA	1352.12788
*E.faecium*_strain_18-276	Rectal swab	2018	Europe	France	Human	1352.12855
*E.faecium*_strain_K2037	Hospital	2018	Europe	Slovakia	Human	1352.12865
*E.faecium*_strain_VZ1530	Hospital	2018	Europe	Slovakia	Human	1352.12867
*E.faecium*_strain_M17773	Hospital	2017	Europe	Slovakia	Human	1352.12873
*E.faecium*_strain_D2385	Rectal swab	2018	Europe	Germany	Human	1352.13115
*E.faecium*_strain_PF6	Raw-frozen dog food	2019	Europe	Portugal	NA	1352.13162
*E.faecium*_strain_SCPM-O-B-8952	Genitourinary system	2020	Europe	Russia	Human	1352.13418
*E.faecium*_strain_SCPM-O-B-8942	Digestive system	2020	Europe	Russia	Human	1352.13421
*E.faecium*_strain_SCPM-O-B-8941	Digestive system	2020	Europe	Russia	Human	1352.13422
*E.faecium*_strain_SCPM-O-B-8940	Digestive system	2020	Europe	Russia	Human	1352.13423
*E.faecium*_strain_E10	Surface patient room	2020	NA	NA	NA	1352.13434
*E.faecium*_strain_SCPM-O-B-8952b	Genitourinary system	2020	Europe	Russia	Human	1352.13661
*E.faecium*_strain_SCPM-O-B-8944	Digestive system	2020	Europe	Russia	Human	1352.13663
*E.faecium*_strain_SCPM-O-B-8942b	Digestive system	2020	Europe	Russia	Human	1352.13664

Strain	Source	Year	Geographic group	Country	Host	BRC ID or GenBank accession no.
*E.faecium*_strain_SCPM-O-B-8941b	Digestive system	2020	Europe	Russia	Human	1352.13665
*E.faecium*_strain_SCPM-O-B-8940b	Digestive system	2020	Europe	Russia	Human	1352.13666
*E.faecium*_strain_E10b	Surface patient room	2020	NA	NA	NA	1352.13682
*E.faecium*_strain_SCPM-O-B-8952c	Genitourinary system	2020	Europe	Russia	Human	1352.13812
*E.faecium*_strain_SCPM-O-B-8944b	Digestive system	2020	Europe	Russia	Human	1352.13814
*E.faecium*_strain_SCPM-O-B-8942c	Digestive system	2020	Europe	Russia	Human	1352.13815
*E.faecium*_strain_SCPM-O-B-8941c	Digestive system	2020	Europe	Russia	Human	1352.13816
*E.faecium*_strain_SCPM-O-B-8940c	Digestive system	2020	Europe	Russia	Human	1352.13817
*E.faecium*_SCPM-O-B-8952	Genitourinary system	2020	Europe	Russia	Human	1352.14133
*E.faecium*_SCPM-O-B-8944	Digestive system	2020	Europe	Russia	Human	1352.14135
*E.faecium*_SCPM-O-B-8942	Digestive system	2020	Europe	Russia	Human	1352.14136
*E.faecium*_SCPM-O-B-8941	Digestive system	2020	Europe	Russia	Human	1352.14137
*E.faecium*_SCPM-O-B-8940	Digestive system	2020	Europe	Russia	Human	1352.14138
*E.faecium*_17-180	Rectal swab	2017	Africa	Reunion	Human	1352.14141
*E.faecium*_18-190	Rectal swab	2018	Africa	Reunion	Human	1352.14145
*E.faecium*_19-213	Rectal swab	2019	Africa	Reunion	Human	1352.14151
*E.faecium*_19-289	Rectal swab	2019	Africa	Reunion	Human	1352.14155
*E.faecium*_E10	Surface patient room	2020	Europe	Germany	NA	1352.14209
*E.faecium*_UK040	Human	2018	Europe	Germany	Human	1352.14273
*E.faecium*_Sample_17	Blood	2019	Europe	Ireland	Human	1352.14274
*E.faecium*_Sample_16	Blood	2019	Europe	Ireland	Human	1352.14275
*E.faecium*_Sample_15	Blood	2019	Europe	Ireland	Human	1352.14277
*E.faecium*_Sample_7	Blood	2019	Europe	Ireland	Human	1352.14278
*E.faecium*_Sample_14	Blood	2019	Europe	Ireland	Human	1352.14279
*E.faecium*_Sample_12	Blood	2019	Europe	Ireland	Human	1352.14281
*E.faecium*_Sample_6	Blood	2019	Europe	Ireland	Human	1352.14283

Strain	Source	Year	Geographic group	Country	Host	BRC ID or GenBank accession no.
*E.faecium*_Sample_5	Blood	2019	Europe	Ireland	Human	1352.14286
*E.faecium*_Sample_2	Blood	2019	Europe	Ireland	Human	1352.14288
*E.faecium*_Sample_27	Blood	2019	Europe	Ireland	Human	1352.14289
*E.faecium*_Sample_24	Blood	2019	Europe	Ireland	Human	1352.1428
*E.faecium*_Sample_25	Blood	2019	Europe	Ireland	Human	1352.14291
*E.faecium*_Sample_10	Blood	2019	Europe	Ireland	Human	1352.14293
*E.faecium*_Sample_19	Blood	2019	Europe	Ireland	Human	1352.14297
*E.faecium*_Sample_18	Blood	2019	Europe	Ireland	Human	1352.14298
*E.faecium*_Sample_20	Blood	2019	Europe	Ireland	Human	1352.14301
*E.faecium*_QAUEFNN4	Dahi fermented milk product	NA	Asia	Pakistan	NA	1352.14325
*E.faecium*_P80Iso2	Blood culture	2019	Europe	Germany	Human	1352.14418
*E.faecium*_P87Iso1	Rectal swab	2019	Europe	Germany	Human	1352.14419
*E.faecium*_P107Iso1	Rectal swab	2019	Europe	Germany	Human	1352.14438
*E.faecium*_P108Iso1	Rectal swab	2019	Europe	Germany	Human	1352.14446
*E.faecium*_P90Iso1	Rectal swab	2019	Europe	Germany	Human	1352.1444
*E.faecium*_P80Iso1	Rectal swab	2019	Europe	Germany	Human	1352.14455
*E.faecium*_P79Iso1	Rectal swab	2019	Europe	Germany	Human	1352.14456
*E.faecium*_349T	Blood	2016	Africa	Tunisia	Human	1352.14459
*E.faecium*_strain_Efm0225	Blood culture	2013	Oceania	Australia	Human	1352.1465
*E.faecium*_VR0722	Human	2021	Asia	Malaysia	Human	1352.14764
*E.faecium*_strain_Efm0211	Blood culture	2012	Oceania	Australia	Human	1352.1482
*E.faecium*_strain_Efm0223	Blood culture	2013	Oceania	Australia	Human	1352.1483
*E.faecium*_AA290	Human	2013	Asia	Japan	Human	1352.14886
*E.faecium*_strain_Efm0215	Blood culture	2012	Oceania	Australia	Human	1352.1492
*E.faecium*_strain_Efm0222	Blood culture	2013	Oceania	Australia	Human	1352.1495
*E.faecium*_strain_Efm0192	Blood culture	2012	Oceania	Australia	Human	1352.1502
*E.faecium*_16ENT29	Blood	2016	Asia	China	Human	1352.15113
*E.faecium*_17-MB2606	Blood	2016	North America	USA	Human	1352.15168
*E.faecium*_45-MB2968	Blood	2016	North America	USA	Human	1352.15181
*E.faecium*_FS100292	Blood	2017	North America	USA	Human	1352.15203
*E.faecium*_FS100693	Blood	2017	North America	USA	Human	1352.15209
*E.faecium*_HH113	Blood	2017	North America	USA	Human	1352.15213
*E.faecium*_152-MB4674	Blood	2017	North America	USA	Human	1352.15216
*E.faecium*_118-MB4137	Blood	2017	North America	USA	Human	1352.15233
*E.faecium*_138-MB4331	Blood	2017	North America	USA	Human	1352.15235

Strain	Source	Year	Geographic group	Country	Host	BRC ID or GenBank accession no.
*E.faecium*_127-MB4229	Blood	2017	North America	USA	Human	1352.15237
*E.faecium*_139-MB4339	Blood	2017	North America	USA	Human	1352.15241
*E.faecium*_strain_Efm0232	Blood culture	2013	Oceania	Australia	Human	1352.1525
*E.faecium*_strain_Efm0157	Blood culture	2011	Oceania	Australia	Human	1352.1526
*E.faecium*_strain_Efm0230	Blood culture	2013	Oceania	Australia	Human	1352.1539
*E.faecium*_EF0656	Urine	2022	Asia	China	Human	1352.15589
*E.faecium*_345357	Blood	2021	Asia	India	Human	1352.15651
*E.faecium*_330767	Pus	2021	Asia	India	Human	1352.1565
*E.faecium*_EFM-R-14	Urine	2021	Europe	France	Human	1352.15671
*E.faecium*_EFM-R-15	Abdominal collection	2020	Europe	France	Human	1352.15672
*E.faecium*_EFM-R-11	Urine	2021	Europe	France	Human	1352.15673
*E.faecium*_EFM-R-23	Blood	2020	Europe	France	Human	1352.15679
*E.faecium*_EFM-R-03	Urine	NA	NA	NA	NA	1352.15687
*E.faecium*_EFM-R-04	Urine	2021	Europe	France	Human	1352.15691
*E.faecium*_EFM-R-06	Urine	2021	Europe	France	Human	1352.15695
*E.faecium*_EFM-R-02	Urine	2021	Europe	France	Human	1352.1569
*E.faecium*_EFM-R-27	Bone biopsy	2020	Europe	France	Human	1352.15712
*E.faecium*_EFM-R-28	Urine	NA	NA	NA	NA	1352.15715
*E.faecium*_EFM-R-01	Blood	2020	Europe	France	Human	1352.15717
*E.faecium*_UAMS_EF106	Urine	2019	North America	USA	Human	1352.16068
*E.faecium*_UAMS_EF105	Blood	2019	North America	USA	Human	1352.16069
*E.faecium*_UAMS_EF72	Blood	2018	North America	USA	Human	1352.16092
*E.faecium*_UAMS_EF95	Urine	2019	North America	USA	Human	1352.16097
*E.faecium*_UAMS_EF88	Urine	2019	North America	USA	Human	1352.16098
*E.faecium*_UAMS_EF93	Urine	2019	North America	USA	Human	1352.16099
*E.faecium*_UAMS_EF73	Blood	2018	North America	USA	Human	1352.16109
*E.faecium*_UAMS_EF63	Urine	2018	North America	USA	Human	1352.16111
*E.faecium*_UAMS_EF61	Urine	2018	North America	USA	Human	1352.16115
*E.faecium*_strain_VRE2	Patient's blood	2011	Asia	Malaysia	Human	1352.1619
*E.faecium*_strain_2G6_DIV0578	Feces	NA	Europe	Germany	Bird	1352.1742
*E.faecium*_strain_2E6_DIV0576	Feces	2015	Europe	Germany	Bird	1352.1755
*E.faecium*_strain_VREN2307	Human	2015	Europe	United Kingdom	Human	1352.1764

Strain	Source	Year	Geographic group	Country	Host	BRC ID or GenBank accession no.
*E.faecium*_strain_VREN2165	Human	2015	Europe	United Kingdom	Human	1352.1765
*E.faecium*_strain_VREN2302	Human	2015	Europe	United Kingdom	Human	1352.1767
*E.faecium*_strain_VREN2303	Human	2015	Europe	United Kingdom	Human	1352.1768
*E.faecium*_strain_VREN3203	Human	2015	Europe	United Kingdom	Human	1352.1771
*E.faecium*_strain_VREN1980	Human	2015	Europe	United Kingdom	Human	1352.1773
*E.faecium*_strain_VREN2170	Human	2015	Europe	United Kingdom	Human	1352.1774
*E.faecium*_strain_VREN2305	Human	2015	Europe	United Kingdom	Human	1352.1776
*E.faecium*_strain_VREN1835	Human	2015	Europe	United Kingdom	Human	1352.1777
*E.faecium*_strain_VREN2300	Human	2015	Europe	United Kingdom	Human	1352.1789
*E.faecium*_strain_VREN2299	Human	2015	Europe	United Kingdom	Human	1352.1793
*E.faecium*_strain_VREN2301	Human	2015	Europe	United Kingdom	Human	1352.1795
*E.faecium*_strain_VREN2164	Human	2015	Europe	United Kingdom	Human	1352.1796
*E.faecium*_strain_VREN1902	Human	2015	Europe	United Kingdom	Human	1352.1801
*E.faecium*_strain_VREN1900	Human	2015	Europe	United Kingdom	Human	1352.1802
*E.faecium*_strain_VREN3652	Human	2015	Europe	United Kingdom	Human	1352.1807
*E.faecium*_strain_VREN2173	Human	2015	Europe	United Kingdom	Human	1352.1808
*E.faecium*_strain_VREN1903	Human	2015	Europe	United Kingdom	Human	1352.1809
*E.faecium*_strain_VREN1840	Human	2015	Europe	United Kingdom	Human	1352.1811

Strain	Source	Year	Geographic group	Country	Host	BRC ID or GenBank accession no.
*E.faecium*_strain_VREN3283	Human	2015	Europe	United Kingdom	Human	1352.1813
*E.faecium*_strain_VREN3280	Human	2015	Europe	United Kingdom	Human	1352.1814
*E.faecium*_strain_VREN3284	Human	2015	Europe	United Kingdom	Human	1352.1816
*E.faecium*_strain_VREN3285	Human	2015	Europe	United Kingdom	Human	1352.1817
*E.faecium*_strain_VREN1532	Human	2015	Europe	United Kingdom	Human	1352.1819
*E.faecium*_strain_VREN1374	Human	2015	Europe	United Kingdom	Human	1352.1821
*E.faecium*_strain_VREN1597	Human	2015	Europe	United Kingdom	Human	1352.1822
*E.faecium*_strain_VREN1600	Human	2015	Europe	United Kingdom	Human	1352.1823
*E.faecium*_strain_VREN3306	Human	2015	Europe	United Kingdom	Human	1352.1824
*E.faecium*_strain_VREN1628	Human	2015	Europe	United Kingdom	Human	1352.1825
*E.faecium*_strain_VREN1624	Human	2015	Europe	United Kingdom	Human	1352.1826
*E.faecium*_strain_VREN3639	Human	2015	Europe	United Kingdom	Human	1352.1827
*E.faecium*_strain_VREN3287	Human	2015	Europe	United Kingdom	Human	1352.1828
*E.faecium*_strain_VREN1534	Human	2015	Europe	United Kingdom	Human	1352.1831
*E.faecium*_strain_VREN1457	Human	2015	Europe	United Kingdom	Human	1352.1832
*E.faecium*_strain_VREN1371	Human	2015	Europe	United Kingdom	Human	1352.1833
*E.faecium*_strain_VREN3644	Human	2015	Europe	United Kingdom	Human	1352.1834
*E.faecium*_strain_VREN2171	Human	2015	Europe	United Kingdom	Human	1352.1837

Strain	Source	Year	Geographic group	Country	Host	BRC ID or GenBank accession no.
*E.faecium*_strain_VREN1458	Human	2015	Europe	United Kingdom	Human	1352.1838
*E.faecium*_strain_VREN1622	Human	2015	Europe	United Kingdom	Human	1352.1839
*E.faecium*_strain_VREN3642	Human	2015	Europe	United Kingdom	Human	1352.1842
*E.faecium*_strain_VREN1627	Human	2015	Europe	United Kingdom	Human	1352.1843
*E.faecium*_strain_VREN1375	Human	2015	Europe	United Kingdom	Human	1352.1844
*E.faecium*_strain_VREN3290	Human	2015	Europe	United Kingdom	Human	1352.1845
*E.faecium*_strain_VREN1630	Human	2015	Europe	United Kingdom	Human	1352.1846
*E.faecium*_strain_VREN1373	Human	2015	Europe	United Kingdom	Human	1352.1847
*E.faecium*_strain_VREN1625	Human	2015	Europe	United Kingdom	Human	1352.1848
*E.faecium*_strain_VREN3645	Human	2015	Europe	United Kingdom	Human	1352.1849
*E.faecium*_strain_VREN3293	Human	2015	Europe	United Kingdom	Human	1352.1851
*E.faecium*_strain_VREN1629	Human	2015	Europe	United Kingdom	Human	1352.1852
*E.faecium*_strain_VREN1533	Human	2015	Europe	United Kingdom	Human	1352.1853
*E.faecium*_strain_VREN3347	Human	2015	Europe	United Kingdom	Human	1352.1854
*E.faecium*_strain_VREN1623	Human	2015	Europe	United Kingdom	Human	1352.1855
*E.faecium*_strain_VREN1626	Human	2015	Europe	United Kingdom	Human	1352.1856
*E.faecium*_strain_VREN1631	Human	2015	Europe	United Kingdom	Human	1352.1857
*E.faecium*_strain_VREN1743	Human	2015	Europe	United Kingdom	Human	1352.1859

Strain	Source	Year	Geographic group	Country	Host	BRC ID or GenBank accession no.
*E.faecium*_strain_VREN1454	Human	2015	Europe	United Kingdom	Human	1352.1861
*E.faecium*_strain_VREN1744	Human	2015	Europe	United Kingdom	Human	1352.1862
*E.faecium*_strain_VREN1772	Human	2015	Europe	United Kingdom	Human	1352.1863
*E.faecium*_strain_VREN1774	Human	2015	Europe	United Kingdom	Human	1352.1864
*E.faecium*_strain_VREN1773	Human	2015	Europe	United Kingdom	Human	1352.1865
*E.faecium*_strain_VREN1775	Human	2015	Europe	United Kingdom	Human	1352.1866
*E.faecium*_strain_VREN1776	Human	2015	Europe	United Kingdom	Human	1352.1867
*E.faecium*_strain_VREN1779	Human	2015	Europe	United Kingdom	Human	1352.1868
*E.faecium*_strain_VREN1781	Human	2015	Europe	United Kingdom	Human	1352.1869
*E.faecium*_strain_VREN1802	Human	2015	Europe	United Kingdom	Human	1352.1871
*E.faecium*_strain_VREN1799	Human	2015	Europe	United Kingdom	Human	1352.1872
*E.faecium*_strain_VREN1798	Human	2015	Europe	United Kingdom	Human	1352.1873
*E.faecium*_strain_VREN1782	Human	2015	Europe	United Kingdom	Human	1352.1874
*E.faecium*_strain_VREN1805	Human	2015	Europe	United Kingdom	Human	1352.1875
*E.faecium*_strain_VREN1803	Human	2015	Europe	United Kingdom	Human	1352.1876
*E.faecium*_strain_VREN1804	Human	2015	Europe	United Kingdom	Human	1352.1877
*E.faecium*_strain_VREN1801	Human	2015	Europe	United Kingdom	Human	1352.1878
*E.faecium*_strain_VREN1844	Human	2015	Europe	United Kingdom	Human	1352.1879

Strain	Source	Year	Geographic group	Country	Host	BRC ID or GenBank accession no.
*E.faecium*_strain_VREN1812	Human	2015	Europe	United Kingdom	Human	1352.1881
*E.faecium*_strain_VREN1839	Human	2015	Europe	United Kingdom	Human	1352.1882
*E.faecium*_strain_VREN3646	Human	2015	Europe	United Kingdom	Human	1352.1883
*E.faecium*_strain_VREN1845	Human	2015	Europe	United Kingdom	Human	1352.1885
*E.faecium*_strain_VREN1843	Human	2015	Europe	United Kingdom	Human	1352.1886
*E.faecium*_strain_VREN3647	Human	2015	Europe	United Kingdom	Human	1352.1887
*E.faecium*_strain_VREN1814	Human	2015	Europe	United Kingdom	Human	1352.1888
*E.faecium*_strain_VREN1834	Human	2015	Europe	United Kingdom	Human	1352.1889
*E.faecium*_strain_VREN1841	Human	2015	Europe	United Kingdom	Human	1352.1891
*E.faecium*_strain_VREN1810	Human	2015	Europe	United Kingdom	Human	1352.1892
*E.faecium*_strain_VREN1811	Human	2015	Europe	United Kingdom	Human	1352.1893
*E.faecium*_strain_VREN3648	Human	2015	Europe	United Kingdom	Human	1352.1894
*E.faecium*_strain_VREN3654	Human	2015	Europe	United Kingdom	Human	1352.1895
*E.faecium*_strain_VREN1809	Human	2015	Europe	United Kingdom	Human	1352.1896
*E.faecium*_strain_VREN1807	Human	2015	Europe	United Kingdom	Human	1352.1897
*E.faecium*_strain_VREN1871	Human	2015	Europe	United Kingdom	Human	1352.1899
*E.faecium*_strain_VREN1815	Human	2015	Europe	United Kingdom	Human	1352.1901
*E.faecium*_strain_VREN1836	Human	2015	Europe	United Kingdom	Human	1352.1902

Strain	Source	Year	Geographic group	Country	Host	BRC ID or GenBank accession no.
*E.faecium*_strain_VREN1813	Human	2015	Europe	United Kingdom	Human	1352.1903
*E.faecium*_strain_VREN3649	Human	2015	Europe	United Kingdom	Human	1352.1904
*E.faecium*_strain_VREN1842	Human	2015	Europe	United Kingdom	Human	1352.1905
*E.faecium*_strain_VREN1873	Human	2015	Europe	United Kingdom	Human	1352.1906
*E.faecium*_strain_VREN3686	Human	2015	Europe	United Kingdom	Human	1352.1907
*E.faecium*_strain_VREN1846	Human	2015	Europe	United Kingdom	Human	1352.1908
*E.faecium*_strain_VREN1808	Human	2015	Europe	United Kingdom	Human	1352.1909
*E.faecium*_strain_VREN1872	Human	2015	Europe	United Kingdom	Human	1352.1911
*E.faecium*_strain_VREN3655	Human	2015	Europe	United Kingdom	Human	1352.1912
*E.faecium*_strain_VREN3656	Human	2015	Europe	United Kingdom	Human	1352.1913
*E.faecium*_strain_VREN3657	Human	2015	Europe	United Kingdom	Human	1352.1914
*E.faecium*_strain_VREN3682	Human	2015	Europe	United Kingdom	Human	1352.1915
*E.faecium*_strain_VREN3684	Human	2015	Europe	United Kingdom	Human	1352.1916
*E.faecium*_strain_VREN3685	Human	2015	Europe	United Kingdom	Human	1352.1917
*E.faecium*_strain_VREN3292	Human	2015	Europe	United Kingdom	NA	1352.1918
*E.faecium*_strain_VREN1745	Human	2015	Europe	United Kingdom	Human	1352.1919
*E.faecium*_strain_VREN1800	Human	2015	Europe	United Kingdom	Human	1352.1921
*E.faecium*_strain_VREN1870	Human	2015	Europe	United Kingdom	Human	1352.1923

Strain	Source	Year	Geographic group	Country	Host	BRC ID or GenBank accession no.
*E.faecium*_strain_VREN3288	Human	2015	Europe	United Kingdom	Human	1352.1925
*E.faecium*_strain_VREN3282	Human	2015	Europe	United Kingdom	Human	1352.1926
*E.faecium*_strain_VREN3294	Human	2015	Europe	United Kingdom	Human	1352.1927
*E.faecium*_strain_VREN3650	Human	2015	Europe	United Kingdom	Human	1352.1928
*E.faecium*_strain_VREN3683	Human	2015	Europe	United Kingdom	Human	1352.1929
*E.faecium*_strain_VREN3206	Human	2015	Europe	United Kingdom	NA	1352.1931
*E.faecium*_strain_VREN3653	Human	2015	Europe	United Kingdom	Human	1352.1932
*E.faecium*_strain_VREN3641	Human	2015	Europe	United Kingdom	NA	1352.1933
*E.faecium*_strain_VREN3651	Human	2015	Europe	United Kingdom	NA	1352.1935
*E.faecium*_strain_VREN3638	Human	2015	Europe	United Kingdom	NA	1352.1936
*E.faecium*_strain_VREN3735	Human	2015	Europe	United Kingdom	NA	1352.1937
*E.faecium*_strain_VREN3643	Human	2015	Europe	United Kingdom	NA	1352.1938
*E.faecium*_strain_VREN3289	Human	2015	Europe	United Kingdom	NA	1352.1939
*E.faecium*_strain_VREN3286	Human	2015	Europe	United Kingdom	Human	1352.1941
*E.faecium*_strain_76	Skin	2014	Europe	Russia	Human	1352.2716
*E.faecium*_strain_SAU27	Human	2009	Asia	Saudi Arabia	Human	1352.3417
*E.faecium*_strain_14-312-1	Rectal swab	2014	Europe	France	Human	1352.3522
*E.faecium*_strain_15-162	Rectal swab	2015	Europe	France	Human	1352.3523
*E.faecium*_strain_AUSMDU00004142	Blood	2015	Oceania	Australia	Human	1352.3594
*E.faecium*_strain_13-009	Rectal swab	2013	Europe	France	Human	1352.3599
*E.faecium*_strain_20	Drainage	2012	Europe	Russia	Human	1352.3624

Strain	Source	Year	Geographic group	Country	Host	BRC ID or GenBank accession no.
*E.faecium*_strain_A16	Rectal swab	2014	Europe	Netherlands	Human	1352.3628
*E.faecium*_strain_KRESVSE0002	Blood	2016	Europe	Norway	Human	1352.4047
*E.faecium*_strain_KRESVRE0005	Blood	2017	Europe	Norway	Human	1352.4049
*E.faecium*_strain_EF_155	Nursing call button in hospital intensive care unit	2016	Asia	Pakistan	NA	1352.4209
*E.faecium*_strain_EF_092	Bedside rail in hospital intensive care unit	2016	Asia	Pakistan	NA	1352.4214
*E.faecium*_strain_EF_264	Bedside rail in hospital intensive care unit	2016	Asia	Pakistan	NA	1352.4217
*E.faecium*_strain_EF_101	Bedside rail in hospital intensive care unit	2016	Asia	Pakistan	NA	1352.4233
*E.faecium*_strain_E8172	Human	NA	NA	NA	NA	1352.4281
*E.faecium*_strain_E7948	Human	NA	NA	NA	NA	1352.4284
*E.faecium*_strain_E8423	Human	NA	NA	NA	NA	1352.4293
*E.faecium*_strain_VRE-1300900	Feces	2013	Europe	Sweden	Human	1352.702
*E.faecium*_strain_VRE-1408197	Feces	2014	Europe	Sweden	Human	1352.744
*E.faecium*_strain_VRE-1407988	Feces	2014	Europe	Sweden	Human	1352.746
*E.faecium*_strain_VRE-1408535	Feces	2014	Europe	Sweden	Human	1352.751
*E.faecium*_strain_EF_545	Infection	2014	Asia	Pakistan	Human	1352.7843
*E.faecium*_strain_EF_542	Infection	2014	Asia	Pakistan	Human	1352.7844
*E.faecium*_strain_EF_543	Infection	2014	Asia	Pakistan	Human	1352.7845
*E.faecium*_strain_EF_548	Infection	2014	Asia	Pakistan	Human	1352.7846
*E.faecium*_strain_EF_533	Hospital surface	2018	North America	USA	NA	1352.7853
*E.faecium*_strain_EF_530	Hospital surface	2018	North America	USA	NA	1352.7855
*E.faecium*_strain_EF_547	Infection	2014	Asia	Pakistan	Human	1352.7877
*E.faecium*_strain_EF_544	Infection	2014	Asia	Pakistan	Human	1352.7879

Strain	Source	Year	Geographic group	Country	Host	BRC ID or GenBank accession no.
*E.faecium*_strain_UAMSEF_50	Blood	2018	North America	USA	Human	1352.7901
*E.faecium*_strain_UAMSEF_14	Blood	2018	North America	USA	Human	1352.7903
*E.faecium*_strain_UAMSEF_51	Blood	2018	North America	USA	Human	1352.7904
*E.faecium*_strain_UAMSEF_16	Blood	2018	North America	USA	Human	1352.7906
*E.faecium*_strain_UAMSEF_34	Blood	2018	North America	USA	Human	1352.7907
*E.faecium*_strain_UAMSEF_13	Blood	2018	North America	USA	Human	1352.7908
*E.faecium*_strain_UAMSEF_36	Blood	2018	North America	USA	Human	1352.7909
*E.faecium*_strain_UAMSEF_22	Blood	2018	North America	USA	Human	1352.7912
*E.faecium*_strain_UAMSEF_19	Blood	2018	North America	USA	Human	1352.7914
*E.faecium*_strain_UAMSEF_48	Blood	2018	North America	USA	Human	1352.7916
*E.faecium*_strain_UAMSEF_37	Blood	2018	North America	USA	Human	1352.7921
*E.faecium*_strain_UAMSEF_15	Blood	2018	North America	USA	Human	1352.7923
*E.faecium*_strain_UAMSEF_43	Blood	2018	North America	USA	Human	1352.7924
*E.faecium*_strain_UAMSEF_17	Blood	2018	North America	USA	Human	1352.7928
*E.faecium*_strain_UAMSEF_18	Blood	2018	North America	USA	Human	1352.7929
*E.faecium*_strain_UAMSEF_11	Blood	2018	North America	USA	Human	1352.7931
*E.faecium*_strain_UAMSEF_05	Blood	2018	North America	USA	Human	1352.7932
*E.faecium*_strain_UAMSEF_07	Blood	2018	North America	USA	Human	1352.7933
*E.faecium*_strain_UAMSEF_04	Blood	2018	North America	USA	Human	1352.7934
*E.faecium*_strain_UAMSEF_10	Blood	2018	North America	USA	Human	1352.7935
*E.faecium*_strain_UAMSEF_03	Blood	2018	North America	USA	Human	1352.7936
*E.faecium*_strain_UAMSEF_02	Blood	2018	North America	USA	Human	1352.7937
*E.faecium*_strain_UAMSEF_12	Blood	2018	North America	USA	Human	1352.7938
*E.faecium*_strain_UAMSEF_06	Blood	2018	North America	USA	Human	1352.7939
*E.faecium*_strain_13-014	Rectal swab	2013	Europe	France	Human	1352.7949
*E.faecium*_strain_Fm4	Feces	2018	Asia	China	Human	1352.8069
*E.faecium*_strain_UAMSEF_01	Human	2018	North America	USA	Human	1352.8073
*E.faecium*_strain_UAMSEF_08	Human	2018	North America	USA	Human	1352.8074
*E.faecium*_strain_UAMSEF_09	Human	2018	North America	USA	Human	1352.8075
*E.faecium*_strain_UAMSEF_20	Human	2018	North America	USA	Human	1352.8076
*E.faecium*_strain_349T_strain_not_applicable	Blood	2016	Africa	Tunisia	Human	1352.8192
*E.faecium*_strain_4928STDY7071352	Faecal	2018	Europe	United Kingdom	Human	1352.8299
*E.faecium*_strain_10702_8#78	Human invasive infection	NA	NA	NA	NA	1352.9452

Strain	Source	Year	Geographic group	Country	Host	BRC ID or GenBank accession no.
*E.faecium*_strain_10702_8#73	Human invasive infection	NA	NA	NA	NA	1352.9456
*E.faecium*_strain_10702_8#93	Human invasive infection	NA	NA	NA	NA	1352.9471
*E.faecium*_strain_10733_7#61	Human invasive infection	NA	NA	NA	NA	1352.9513
*E.faecium*_strain_10733_7#80	Human invasive infection	NA	NA	NA	NA	1352.9525
*E.faecium*_strain_10733_8#5	Human invasive infection	NA	NA	NA	NA	1352.9559
*E.faecium*_strain_10770_1#27	Human invasive infection	NA	NA	NA	NA	1352.9602
*E.faecium*_strain_10770_1#46	Human invasive infection	NA	NA	NA	NA	1352.9614
*E.faecium*_strain_10770_1#89	Human invasive infection	NA	NA	NA	NA	1352.9642
*E.faecium*_strain_10900_1#5	Human invasive infection	NA	NA	NA	NA	1352.9649
*E.faecium*_strain_10733_5#92	Human invasive infection	NA	NA	NA	NA	1352.9755
*E.faecium*_strain_10733_4#9	Human invasive infection	NA	NA	NA	NA	1352.9789
*E.faecium*_strain_10733_3#18	Human invasive infection	NA	NA	NA	NA	1352.9791
*E.faecium*_strain_10733_3#10	Human invasive infection	NA	NA	NA	NA	1352.9792
*E.faecium*_strain_10733_3#9	Human invasive infection	NA	NA	NA	NA	1352.9793
*E.faecium*_strain_10733_5#57	Human invasive infection	NA	NA	NA	NA	1352.9795
*E.faecium*_strain_10733_6#12	Human invasive infection	NA	NA	NA	NA	1352.9832
*E.faecium*_strain_10733_4#33	Human invasive infection	NA	NA	NA	NA	1352.9926
*E.faecium*_strain_10733_5#69	Human invasive infection	NA	NA	NA	NA	1352.9927

Strain	Source	Year	Geographic group	Country	Host	BRC ID or GenBank accession no.
*E.faecium*_strain_10702_8#57	Human invasive infection	NA	NA	NA	NA	1352.9934
*E.faecium*_strain_10900_4#70	Human invasive infection	NA	NA	NA	NA	1352.9981
SZYSC_21VRE003	Urine	202110	Asia	Guangzhou	Human	GCA_037477685.1
SZYSC_21VRE007	Urine	202110	Asia	Guangzhou	Human	GCA_037477645.1
SZYSC_22VRE30	Urine	202209	Asia	Guangzhou	Human	GCA_037477665.1
SZYSC_22VRE31	Urine	202210	Asia	Guangzhou	Human	GCA_037477625.1
SZYSC_22VRE32	Urine	202210	Asia	Guangzhou	Human	GCA_037477605.1
SZYSC_23VRE020	Urine	202305	Asia	Guangzhou	Human	GCA_037477585.1
SZYSC_23VRE002	Ascitic fluid	202301	Asia	Guangzhou	Human	GCA_037477565.1
SZYSC_23VRE003	Urine	202301	Asia	Guangzhou	Human	GCA_037477525.1
SZYSC_23VRE004	Urine	202302	Asia	Guangzhou	Human	GCA_037477545.1
SZYSC_23VRE005	Csf	202301	Asia	Guangzhou	Human	GCA_037477505.1
SZYSC_23VRE007	Urine	202302	Asia	Guangzhou	Human	GCA_037477485.1
SZYSC_23VRE009	Urine	202303	Asia	Guangzhou	Human	GCA_037477425.1
SZYSC_23VRE010	Ascitic fluid	202303	Asia	Guangzhou	Human	GCA_037477445.1
SZYSC_23VRE011	Surgical site	202303	Asia	Guangzhou	Human	GCA_037477405.1
SZYSC_23VRE012	Urine	202303	Asia	Guangzhou	Human	GCA_037477385.1
SZYSC_23VRE013	Urine	202303	Asia	Guangzhou	Human	GCA_037477365.1
SZYSC_23VRE014	Urine	202303	Asia	Guangzhou	Human	GCA_037477345.1
SZYSC_23VRE015	Other	202303	Asia	Guangzhou	Human	GCA_037477325.1
SZYSC_23VRE016	Urine	202303	Asia	Guangzhou	Human	GCA_037477305.1
SZYSC_23VRE017	Urine	202304	Asia	Guangzhou	Human	GCA_037477265.1
SZYSC_23VRE018	Urine	202304	Asia	Guangzhou	Human	GCA_037477285.1
SZYSC_23VRE019	Blood	202304	Asia	Guangzhou	Human	GCA_037477245.1
SZYSC_BHVRE006	Surgical site	202207	Asia	Others	Human	GCA_037477165.1
SZYSC_DHVRE001	Ascitic fluid	202204	Asia	Guangdong	Human	GCA_037477065.1
SZYSC_DYVRE002	Urine	202106	Asia	Guangzhou	Human	GCA_037477005.1
SZYSC_DYVRE003	Urine	202107	Asia	Guangzhou	Human	GCA_037476985.1
SZYSC_DYVRE004	Urine	202109	Asia	Guangzhou	Human	GCA_037476965.1
SZYSC_DYVRE005	Urine	202110	Asia	Guangzhou	Human	GCA_037476945.1
SZYSC_DYVRE006	Urine	202110	Asia	Guangzhou	Human	GCA_037476925.1
SZYSC_DYVRE007	Urine	202110	Asia	Guangzhou	Human	GCA_037476905.1
SZYSC_DYVRE008	Urine	202111	Asia	Guangzhou	Human	GCA_037476885.1
SZYSC_DYVRE009	Urine	202201	Asia	Guangzhou	Human	GCA_037476865.1

Strain	Source	Year	Geographic group	Country	Host	BRC ID or GenBank accession no.
SZYSC_DYVRE010	Urine	202201	Asia	Guangzhou	Human	GCA_037476845.1
SZYSC_DYVRE011	Urine	202201	Asia	Guangzhou	Human	GCA_037476825.1
SZYSC_DYVRE012	Urine	202201	Asia	Guangzhou	Human	GCA_037476805.1
SZYSC_DYVRE013	Urine	202201	Asia	Guangzhou	Human	GCA_037476785.1
SZYSC_DYVRE014	Urine	202202	Asia	Guangzhou	Human	GCA_037476745.1
SZYSC_DYVRE015	Urine	202202	Asia	Guangzhou	Human	GCA_037476725.1
SZYSC_DYVRE016	Urine	202202	Asia	Guangzhou	Human	GCA_037476765.1
SZYSC_DYVRE017	Blood	202202	Asia	Guangzhou	Human	GCA_037476705.1
SZYSC_DYVRE018	Blood	202202	Asia	Guangzhou	Human	GCA_037476665.1
SZYSC_DYVRE019	Blood	202202	Asia	Guangzhou	Human	GCA_037476685.1
SZYSC_DYVRE020	Urine	202204	Asia	Guangzhou	Human	GCA_037476625.1
SZYSC_DYVRE021	Other	202204	Asia	Guangzhou	Human	GCA_037476645.1
SZYSC_DYVRE022	Urine	202305	Asia	Guangzhou	Human	GCA_037476605.1
SZYSC_DYVRE023	Urine	202305	Asia	Guangzhou	Human	GCA_037476565.1
SZYSC_DYVRE024	Urine	202305	Asia	Guangzhou	Human	GCA_037476585.1
SZYSC_DYVRE025	Urine	202305	Asia	Guangzhou	Human	GCA_037476525.1
SZYSC_DYVRE026	Urine	202305	Asia	Guangzhou	Human	GCA_037476545.1
SZYSC_DYVRE027	Urine	202209	Asia	Guangzhou	Human	GCA_037476505.1
SZYSC_DYVRE028	Urine	202303	Asia	Guangzhou	Human	GCA_037476485.1
SZYSC_DYVRE029	Urine	202304	Asia	Guangzhou	Human	GCA_037476465.1
SZYSC_DYVRE030	Urine	202304	Asia	Guangzhou	Human	GCA_037476425.1
SZYSC_E010	Urine	202203	Asia	Guangzhou	Human	GCA_037476385.1
SZYSC_E012	Bile	202203	Asia	Guangzhou	Human	GCA_037476325.1
SZYSC_E013	Surgical site	202203	Asia	Guangzhou	Human	GCA_037476305.1
SZYSC_E018	Urine	202205	Asia	Guangzhou	Human	GCA_037476245.1
SZYSC_E019	Urine	202205	Asia	Guangzhou	Human	GCA_037476205.1
SZYSC_E020	Urine	202205	Asia	Guangzhou	Human	GCA_037476225.1
SZYSC_E022	Urine	202205	Asia	Guangzhou	Human	GCA_037476185.1
SZYSC_E023	Urine	202206	Asia	Guangzhou	Human	GCA_037476145.1
SZYSC_E024	Urine	202206	Asia	Guangzhou	Human	GCA_037476125.1
SZYSC_E025	Urine	202206	Asia	Guangzhou	Human	GCA_037476105.1
SZYSC_FSVRE001	Urine	202210	Asia	Guangdong	Human	GCA_037476065.1
SZYSC_FSVRE002	Urine	202210	Asia	Guangdong	Human	GCA_037476045.1
SZYSC_FSVRE004	Urine	202106	Asia	Guangdong	Human	GCA_037476025.1
SZYSC_FSVRE005	Surgical site	202103	Asia	Guangdong	Human	GCA_037475965.1
SZYSC_FSVRE006	Urine	202104	Asia	Guangdong	Human	GCA_037475985.1

Strain	Source	Year	Geographic group	Country	Host	BRC ID or GenBank accession no.
SZYSC_FSVRE007	Urine	202108	Asia	Guangdong	Human	GCA_037476005.1
SZYSC_FSVRE008	Urine	202107	Asia	Guangdong	Human	GCA_037475945.1
SZYSC_FSVRE009	Urine	202103	Asia	Guangdong	Human	GCA_037475925.1
SZYSC_FSVRE010	Urine	202106	Asia	Guangdong	Human	GCA_037475865.1
SZYSC_FSYVRE001	Ascitic fluid	202101	Asia	Guangdong	Human	GCA_037475905.1
SZYSC_FSYVRE002	Urine	202105	Asia	Guangdong	Human	GCA_037475845.1
SZYSC_FSYVRE003	Urine	202108	Asia	Guangdong	Human	GCA_037475885.1
SZYSC_FSYVRE004	Surgical site	202105	Asia	Guangdong	Human	GCA_037475825.1
SZYSC_FSYVRE005	Bile	202102	Asia	Guangdong	Human	GCA_037475805.1
SZYSC_FSYVRE006	Urine	202204	Asia	Guangdong	Human	GCA_037475745.1
SZYSC_FSYVRE007	Urine	202110	Asia	Guangdong	Human	GCA_037475765.1
SZYSC_FSYVRE008	Urine	202106	Asia	Guangdong	Human	GCA_037475785.1
SZYSC_FSYVRE009	Blood	202106	Asia	Guangdong	Human	GCA_037475725.1
SZYSC_FSYVRE010	Urine	202105	Asia	Guangdong	Human	GCA_037475705.1
SZYSC_FSYVRE011	Urine	202112	Asia	Guangdong	Human	GCA_037475685.1
SZYSC_FSYVRE012	Urine	202201	Asia	Guangdong	Human	GCA_037475625.1
SZYSC_FSYVRE013	Urine	202103	Asia	Guangdong	Human	GCA_037475645.1
SZYSC_FSYVRE014	Ascitic fluid	202205	Asia	Guangdong	Human	GCA_037475665.1
SZYSC_FSYVRE015	Urine	202102	Asia	Guangdong	Human	GCA_037475605.1
SZYSC_FSYVRE016	Urine	202203	Asia	Guangdong	Human	GCA_037475585.1
SZYSC_FSYVRE017	Urine	202204	Asia	Guangdong	Human	GCA_037475565.1
SZYSC_FSYVRE018	Urine	202201	Asia	Guangdong	Human	GCA_037475545.1
SZYSC_FSYVRE019	Urine	202203	Asia	Guangdong	Human	GCA_037475525.1
SZYSC_FSYVRE020	Urine	202210	Asia	Guangdong	Human	GCA_037475505.1
SZYSC_FSYVRE021	Ascitic fluid	202211	Asia	Guangdong	Human	GCA_037475485.1
SZYSC_FSYVRE022	Urine	202211	Asia	Guangdong	Human	GCA_037475465.1
SZYSC_FSYVRE023	Urine	202211	Asia	Guangdong	Human	GCA_037475445.1
SZYSC_FSYVRE024	Urine	202211	Asia	Guangdong	Human	GCA_037475405.1
SZYSC_FSYVRE025	Ascitic fluid	202110	Asia	Guangdong	Human	GCA_037475425.1
SZYSC_GYSVRE001	Urine	202110	Asia	Guangzhou	Human	GCA_037475045.1
SZYSC_GYSVRE003	Urine	202111	Asia	Guangzhou	Human	GCA_037475005.1
SZYSC_GYSVRE004	Urine	202206	Asia	Guangzhou	Human	GCA_037474925.1
SZYSC_LNVRE001	Urine	202201	Asia	Others	Human	GCA_037474465.1
SZYSC_LZVRE001	Ascitic fluid	202202	Asia	Guangdong	Human	GCA_037474505.1
SZYSC_LZVRE002	Urine	202207	Asia	Guangdong	Human	GCA_037474445.1
SZYSC_LZVRE003	Urine	202206	Asia	Guangdong	Human	GCA_037474485.1

Strain	Source	Year	Geographic group	Country	Host	BRC ID or GenBank accession no.
SZYSC_LZVRE004	Urine	202207	Asia	Guangdong	Human	GCA_037474425.1
SZYSC_LZVRE008	Blood	202205	Asia	Guangdong	Human	GCA_037474385.1
SZYSC_MZVRE001	Surgical site	202203	Asia	Guangdong	Human	GCA_037474405.1
SZYSC_MZVRE002	Surgical site	202208	Asia	Guangdong	Human	GCA_037474365.1
SZYSC_MZVRE003	Bile	202208	Asia	Guangdong	Human	GCA_037474345.1
SZYSC_MZVRE004	Urine	202207	Asia	Guangdong	Human	GCA_037474325.1
SZYSC_MZVRE005	Urine	202207	Asia	Guangdong	Human	GCA_037474305.1
SZYSC_MZVRE006	Urine	202205	Asia	Guangdong	Human	GCA_037474285.1
SZYSC_MZVRE007	Urine	202205	Asia	Guangdong	Human	GCA_037474265.1
SZYSC_MZVRE008	Ascitic fluid	202206	Asia	Guangdong	Human	GCA_037474225.1
SZYSC_MZVRE009	Bile	202206	Asia	Guangdong	Human	GCA_037474245.1
SZYSC_MZVRE010	Blood	202112	Asia	Guangdong	Human	GCA_037474165.1
SZYSC_MZVRE011	Urine	202112	Asia	Guangdong	Human	GCA_037474205.1
SZYSC_MZVRE012	Blood	202111	Asia	Guangdong	Human	GCA_037474145.1
SZYSC_MZVRE013	Urine	202207	Asia	Guangdong	Human	GCA_037474125.1
SZYSC_MZVRE014	Surgical site	202207	Asia	Guangdong	Human	GCA_037474185.1
SZYSC_NXVRE002	Blood	201704	Asia	Others	Human	GCA_037474085.1
SZYSC_PYVRE001	Blood	202210	Asia	Guangzhou	Human	GCA_037473975.1
SZYSC_QRYVRE001	Urine	202210	Asia	Guangdong	Human	GCA_037473955.1
SZYSC_QRYVRE002	Urine	202211	Asia	Guangdong	Human	GCA_037473935.1
SZYSC_QYVRE001	Urine	202201	Asia	Guangdong	Human	GCA_037473915.1
SZYSC_QYVRE002	Urine	202204	Asia	Guangdong	Human	GCA_037473895.1
SZYSC_QYVRE003	Urine	202205	Asia	Guangdong	Human	GCA_037473875.1
SZYSC_SCVRE001	Surgical site	201909	Asia	Others	Human	GCA_037473855.1
SZYSC_SCVRE009	Urine	202109	Asia	Others	Human	GCA_037473715.1
SZYSC_SCVRE011	Ascitic fluid	202206	Asia	Others	Human	GCA_037473675.1
SZYSC_SCVRE012	Ascitic fluid	202207	Asia	Others	Human	GCA_037473595.1
SZYSC_SCVRE013	Urine	202201	Asia	Others	Human	GCA_037473575.1
SZYSC_SCVRE014	Surgical site	202112	Asia	Others	Human	GCA_037473635.1
SZYSC_SYVRE001	Ascitic fluid	202112	Asia	Guangzhou	Human	GCA_037473615.1
SZYSC_SYVRE003	Urine	202101	Asia	Guangzhou	Human	GCA_037473565.1
SZYSC_SYVRE006	Urine	202109	Asia	Guangzhou	Human	GCA_037473505.1
SZYSC_SYVRE011	Urine	202101	Asia	Guangzhou	Human	GCA_037473515.1
SZYSC_SYVRE012	Wound	202107	Asia	Guangzhou	Human	GCA_037473525.1
SZYSC_SYVRE013	Urine	202102	Asia	Guangzhou	Human	GCA_037473485.1
SZYSC_SYVRE015	Urine	202107	Asia	Guangzhou	Human	GCA_037473465.1

Strain	Source	Year	Geographic group	Country	Host	BRC ID or GenBank accession no.
SZYSC_VRE001	Urine	202201	Asia	Guangzhou	Human	GCA_037473215.1
SZYSC_VRE005	Urine	202202	Asia	Guangzhou	Human	GCA_037473125.1
SZYSC_VRE006	Urine	202202	Asia	Guangzhou	Human	GCA_037473045.1
SZYSC_ZDYVRE003	Bile	202202	Asia	Guangzhou	Human	GCA_037472975.1
SZYSC_ZDYVRE004	Bile	202202	Asia	Guangzhou	Human	GCA_037472905.1
SZYSC_ZDYVRE005	Urine	202202	Asia	Guangzhou	Human	GCA_037472865.1
SZYSC_ZDYVRE006	Surgical site	202202	Asia	Guangzhou	Human	GCA_037472805.1
SZYSC_ZDYVRE007	Surgical site	202204	Asia	Guangzhou	Human	GCA_037472765.1
SZYSC_ZDYVRE008	Urine	202204	Asia	Guangzhou	Human	GCA_037472705.1
SZYSC_ZDYVRE009	Surgical site	202204	Asia	Guangzhou	Human	GCA_037472715.1
SZYSC_ZDYVRE010	Surgical site	202205	Asia	Guangzhou	Human	GCA_037472685.1
SZYSC_ZDYVRE011	Urine	202205	Asia	Guangzhou	Human	GCA_037472545.1
SZYSC_ZDYVRE012	Urine	202205	Asia	Guangzhou	Human	GCA_037472505.1
SZYSC_ZDYVRE013	Urine	202205	Asia	Guangzhou	Human	GCA_037472555.1
SZYSC_ZDYVRE014	Surgical site	202205	Asia	Guangzhou	Human	GCA_037472515.1
SZYSC_ZDYVRE015	Urine	202205	Asia	Guangzhou	Human	GCA_037472445.1
SZYSC_ZDYVRE017	Urine	202206	Asia	Guangzhou	Human	GCA_037472365.1
SZYSC_ZDYVRE018	Surgical site	202207	Asia	Guangzhou	Human	GCA_037472385.1
SZYSC_ZDYVRE019	Surgical site	202207	Asia	Guangzhou	Human	GCA_037472375.1
SZYSC_ZDYVRE021	Urine	202208	Asia	Guangzhou	Human	GCA_037472235.1
SZYSC_ZDYVRE023	Blood	202209	Asia	Guangzhou	Human	GCA_037472175.1
SZYSC_ZDYVRE027	Urine	202210	Asia	Guangzhou	Human	GCA_037472165.1
SZYSC_ZDYVRE028	Wound	202211	Asia	Guangzhou	Human	GCA_037472145.1
SZYSC_ZDYVRE029	Blood	202209	Asia	Guangzhou	Human	GCA_037472125.1
SZYSC_ZDYVRE030	Blood	202211	Asia	Guangzhou	Human	GCA_037472095.1
SZYSC_ZDYVRE031	Sputum	202211	Asia	Guangzhou	Human	GCA_037472065.1
SZYSC_ZDYVRE032	Urine	202211	Asia	Guangzhou	Human	GCA_037472085.1
SZYSC_ZDYVRE033	Urine	202211	Asia	Guangzhou	Human	GCA_037472045.1
SZYSC_ZDYVRE035	Urine	202212	Asia	Guangzhou	Human	GCA_037472005.1
SZYSC_ZDYVRE037	Urine	202301	Asia	Guangzhou	Human	GCA_037472025.1
SZYSC_ZDYVRE038	Urine	202201	Asia	Guangzhou	Human	GCA_037471945.1
SZYSC_ZDYVRE039	Urine	202301	Asia	Guangzhou	Human	GCA_037471975.1
SZYSC_ZDYVRE045	Sputum	202302	Asia	Guangzhou	Human	GCA_037471845.1
SZYSC_ZDYVRE046	Wound	202303	Asia	Guangzhou	Human	GCA_037471875.1
SZYSC_ZDYVRE047	Urine	202303	Asia	Guangzhou	Human	GCA_037471905.1
SZYSC_ZDYVRE050	Urine	202304	Asia	Guangzhou	Human	GCA_037471825.1

Strain	Source	Year	Geographic group	Country	Host	BRC ID or GenBank accession no.
SZYSC_ZDYVRE051	Blood	202304	Asia	Guangzhou	Human	GCA_037471805.1
SZYSC_ZDYVRE052	Urine	202304	Asia	Guangzhou	Human	GCA_037471755.1
SZYSC_ZDYVRE053	Blood	202304	Asia	Guangzhou	Human	GCA_037471765.1
SZYSC_ZDYVRE055	Urine	202304	Asia	Guangzhou	Human	GCA_037471745.1
SZYSC_ZDYVRE057	Urine	202305	Asia	Guangzhou	Human	GCA_037471675.1
SZYSC_ZDYVRE058	Surgical site	202305	Asia	Guangzhou	Human	GCA_037471705.1
SZYSC_ZDYVRE060	Surgical site	202305	Asia	Guangzhou	Human	GCA_037471635.1
SZYSC_ZJVRE001	Bile	202206	Asia	Guangzhou	Human	GCA_037471625.1
SZYSC_ZJVRE002	Ascitic fluid	202206	Asia	Guangzhou	Human	GCA_037471545.1
SZYSC_ZJVRE003	Wound	202206	Asia	Guangzhou	Human	GCA_037471505.1
SZYSC_ZJVRE004	Urine	202207	Asia	Guangzhou	Human	GCA_037471375.1
SZYSC_ZJVRE005	Bile	202208	Asia	Guangzhou	Human	GCA_037471445.1
SZYSC_ZJVRE006	Bile	202208	Asia	Guangzhou	Human	GCA_037471365.1
SZYSC_ZJVRE007	Ascitic fluid	202209	Asia	Guangzhou	Human	GCA_037471325.1
SZYSC_ZJVRE008	Urine	202209	Asia	Guangzhou	Human	GCA_037471175.1
SZYSC_ZJVRE009	Ascitic fluid	202209	Asia	Guangzhou	Human	GCA_037471255.1
SZYSC_ZJVRE010	Urine	202209	Asia	Guangzhou	Human	GCA_037471165.1
SZYSC_ZJVRE013	Urine	202210	Asia	Guangzhou	Human	GCA_037470995.1
SZYSC_ZSRVRE001	Surgical site	202208	Asia	Guangdong	Human	GCA_037470965.1
SZYSC_ZSRVRE002	Urine	202208	Asia	Guangdong	Human	GCA_037470955.1
SZYSC_ZSRVRE003	Urine	202205	Asia	Guangdong	Human	GCA_037470985.1
SZYSC_ZSRVRE004	Urine	202207	Asia	Guangdong	Human	GCA_037470845.1
SZYSC_ZSRVRE005	Urine	202210	Asia	Guangdong	Human	GCA_037470795.1
SZYSC_ZSVRE001	Urine	202106	Asia	Guangzhou	Human	GCA_037470735.1
SZYSC_ZSVRE002	Urine	202107	Asia	Guangzhou	Human	GCA_037470705.1
SZYSC_ZSVRE003	Bile	202209	Asia	Guangzhou	Human	GCA_037470695.1
SZYSC_ZSVRE004	Urine	202108	Asia	Guangzhou	Human	GCA_037470665.1
SZYSC_ZSVRE005	Urine	202109	Asia	Guangzhou	Human	GCA_037470495.1
SZYSC_BHVRE007	Urine	202212	Asia	Others	Human	GCA_037470505.1
SZYSC_BHVRE010	Csf	202303	Asia	Others	Human	GCA_037470455.1
SZYSC_BHVRE013	Urine	202305	Asia	Others	Human	GCA_037470225.1
SZYSC_BHVRE016	Urine	202306	Asia	Others	Human	GCA_037470165.1

*Csf, cerebrospinal fluid; NA, not available.

Appendix 1 Table 3. Distribution of virulence factor genes in SC11 isolates

Sample	*scm*	*PilA*	*Esp*	*sgrAc*	*PilB*	*acm*	*psaA*	*efaA*	FBP	*ecbA/fss3*	*plr/gapA*	*bopD*	*clpP*	*cpsA/uppS*	*cpsB/cdsA*	*eno*	*rfbB*	Total
RE_TPH_010	1	1	1	1	1	1	2	1	1	1	1	1	1	1	1	1	1	18
VRE_TPH_011	1	1	1	1	1	1	1	1	1	1	1	1	1	1	1	1	1	17
VRE_TPH_012	1	1	1	1	1	1	2	1	1	1	1	1	1	1	1	1	1	18
VRE_TPH_013	0	1	1	1	1	1	2	1	1	1	1	1	1	1	1	1	1	17
VRE_TPH_014	1	1	1	1	1	1	2	1	1	1	1	1	1	1	1	1	1	18
VRE_TPH_015	1	1	1	1	1	1	2	1	1	1	1	1	1	1	1	1	1	18
VRE_TPH_016	0	1	1	1	1	1	2	1	1	1	1	1	1	1	1	1	1	17
VRE_TPH_017	1	1	1	1	1	1	2	1	1	1	1	1	1	1	1	1	1	18
VRE_TPH_018	1	1	1	1	1	1	2	1	1	1	1	1	1	1	1	1	1	18
VRE_TPH_019	1	1	1	1	1	1	2	1	1	1	1	1	1	1	1	1	1	18
VRE_TPH_001	0	1	1	1	1	1	2	1	1	1	1	1	1	1	1	1	1	17
VRE_SPH_002	1	2	1	1	1	1	2	1	1	1	1	1	1	1	1	1	1	19
VRE_NSPH_002	1	0	1	1	1	1	2	1	1	1	1	1	1	1	1	1	1	17
VRE_PSPH_003	1	1	1	1	1	1	1	1	1	1	1	1	1	1	1	1	1	17
VRE_NSPH_001	1	1	1	1	1	1	2	1	1	1	1	1	1	1	1	1	1	18
VRE_PSPH_001	1	1	1	1	1	1	1	1	1	1	1	1	1	1	1	1	1	17
VRE_PSPH_004	1	1	1	1	1	1	1	1	1	1	1	1	1	1	1	1	1	17
VRE_PSPH_002	1	1	1	1	1	1	2	1	1	1	1	1	1	1	1	1	1	18
VRE_NSPH_003	0	1	1	1	0	1	2	1	1	1	1	1	1	1	1	1	1	16
VRE_TPH_002	0	1	1	1	1	1	2	1	1	1	1	1	1	1	1	1	1	17
VRE_SPH_003	1	1	1	1	1	1	2	1	1	1	1	1	1	1	1	1	1	18
VRE_TPH_003	1	1	1	1	1	1	2	1	1	1	1	1	1	1	1	1	1	18
VRE_SPH_004	1	1	1	1	1	1	2	1	1	1	1	1	1	1	1	1	1	18
VRE_TPH_004	0	1	1	1	1	1	2	1	1	1	1	1	1	1	1	1	1	17
VRE_SPH_005	0	1	2	1	1	1	2	1	1	1	1	1	1	1	1	1	1	18

Sample	*scm*	*PilA*	*Esp*	*sgrAc*	*PilB*	*acm*	*psaA*	*efaA*	FBP	*ecbA/fss3*	*plr/gapA*	*bopD*	*clpP*	*cpsA/uppS*	*cpsB/cdsA*	*eno*	*rfbB*	Total
VRE_SPH_006	1	1	1	1	1	1	2	1	1	1	1	1	1	1	1	1	1	18
VRE_TPH_006	1	1	1	1	1	1	2	1	1	1	1	1	1	1	1	1	1	18
VRE_TPH_007	1	1	1	1	1	1	1	1	1	1	1	1	1	1	1	1	1	17
VRE_TPH_008	1	1	1	1	1	1	2	1	1	1	1	1	1	1	1	1	1	18
VRE_TPH_009	0	1	1	1	1	1	2	1	1	1	1	1	1	1	1	1	1	17
VRE_SZCMH_001	1	1	1	1	1	1	2	1	1	1	1	1	1	1	1	1	1	18
VRE_SZCMH_002	1	1	1	1	1	1	2	1	1	1	1	1	1	1	1	1	1	18
VRE_SZCMH_003	1	1	1	1	1	1	2	1	1	1	1	1	1	1	1	1	1	18
VRE_SZCMH_004	1	1	1	1	1	1	2	1	1	1	1	1	1	1	1	1	1	18
VRE_SZCMH_005	1	1	1	1	1	1	2	1	1	1	1	1	1	1	1	1	1	18
VRE_SZCMH_006	1	0	1	1	1	1	2	1	1	1	1	1	1	1	1	1	1	17
SZYSC_BHVRE016	0	0	1	1	1	1	2	1	1	1	1	1	1	1	1	1	1	16
SZYSC_ZSVRE005	0	1	1	1	1	1	2	1	1	1	1	1	1	1	1	1	1	17
SZYSC_ZSVRE004	0	1	1	1	1	1	2	1	1	1	1	1	1	1	1	1	1	17
SZYSC_ZSVRE003	0	2	1	1	1	1	2	1	1	1	1	1	1	1	1	1	1	18
SZYSC_ZSVRE002	0	0	1	1	1	1	2	1	1	1	1	1	1	1	1	1	1	16
SZYSC_ZSVRE001	0	1	1	1	1	1	2	1	1	1	1	1	1	1	1	1	1	17
SZYSC_ZSRVRE005	0	1	1	1	1	1	2	1	1	1	1	1	1	1	1	1	1	17
SZYSC_ZSRVRE004	0	1	1	1	1	1	1	1	1	1	1	1	1	1	1	1	1	16
SZYSC_ZSRVRE002	0	1	1	1	1	1	1	1	1	1	1	1	1	1	1	1	1	16
SZYSC_ZSRVRE001	0	1	1	1	1	1	2	1	1	1	1	1	1	1	1	1	1	17
SZYSC_ZSRVRE003	0	1	1	1	1	1	2	1	1	1	1	1	1	1	1	1	1	17
SZYSC_ZJVRE013	0	1	1	1	1	1	2	1	1	1	1	1	1	1	1	1	1	17
SZYSC_ZJVRE010	0	1	1	1	1	1	2	1	1	1	1	1	1	1	1	1	1	17
SZYSC_ZJVRE008	0	1	1	1	1	1	2	1	1	1	1	1	1	1	1	1	1	17
SZYSC_ZJVRE009	0	2	1	1	1	0	2	1	1	1	1	1	1	1	1	1	1	17

Sample	*scm*	*PilA*	*Esp*	*sgrAc*	*PilB*	*acm*	*psaA*	*efaA*	FBP	*ecbA/fss3*	*plr/gapA*	*bopD*	*clpP*	*cpsA/uppS*	*cpsB/cdsA*	*eno*	*rfbB*	Total
SZYSC_ZJVRE007	0	1	1	1	1	1	2	1	1	1	1	1	1	1	1	1	1	17
SZYSC_ZJVRE006	0	1	1	1	1	1	2	1	1	1	1	1	1	1	1	1	1	17
SZYSC_ZJVRE004	0	1	1	1	1	1	2	1	1	1	1	1	1	1	1	1	1	17
SZYSC_ZJVRE005	0	1	1	1	1	1	2	1	1	1	1	1	1	1	1	1	1	17
SZYSC_ZJVRE003	0	1	1	1	1	1	2	1	1	1	1	1	1	1	1	1	1	17
SZYSC_ZJVRE002	0	1	1	1	1	1	2	1	1	1	1	1	1	1	1	1	1	17
SZYSC_ZJVRE001	0	1	1	1	1	1	2	1	1	1	1	1	1	1	1	1	1	17
SZYSC_ZDYVRE060	0	1	1	1	1	1	2	1	1	1	1	1	1	1	1	1	1	17
SZYSC_ZDYVRE057	0	1	1	1	1	1	2	1	1	1	1	1	1	1	1	1	1	17
SZYSC_ZDYVRE058	0	2	1	1	1	1	2	1	1	1	1	1	1	1	1	1	1	18
SZYSC_ZDYVRE055	1	1	1	1	1	1	2	1	1	1	1	1	1	1	1	1	1	18
SZYSC_ZDYVRE052	1	1	1	1	1	1	2	1	1	1	1	1	1	1	1	1	1	18
SZYSC_ZDYVRE053	0	1	1	1	1	1	2	1	1	1	1	1	1	1	1	1	1	17
SZYSC_ZDYVRE051	0	0	1	1	1	1	2	1	1	1	1	1	1	1	1	1	1	16
SZYSC_ZDYVRE050	0	1	1	1	1	1	2	1	1	1	1	1	1	1	1	1	1	17
SZYSC_ZDYVRE045	0	1	1	1	1	1	2	1	1	1	1	1	1	1	1	1	1	17
SZYSC_ZDYVRE046	0	1	1	1	1	1	2	1	1	1	1	1	1	1	1	1	1	17
SZYSC_ZDYVRE047	0	0	1	1	1	1	2	1	1	1	1	1	1	1	1	1	1	16
SZYSC_ZDYVRE038	0	0	1	1	1	1	2	1	1	1	1	1	1	1	1	1	1	16
SZYSC_ZDYVRE039	0	1	1	1	1	1	2	1	1	1	1	1	1	1	1	1	1	17
SZYSC_ZDYVRE035	0	0	1	1	1	1	2	1	1	1	1	1	1	1	1	1	1	16
SZYSC_ZDYVRE037	0	1	1	1	1	1	2	1	1	1	1	1	1	1	1	1	1	17
SZYSC_ZDYVRE033	0	1	1	1	1	1	2	1	1	1	1	1	1	1	1	1	1	17
SZYSC_ZDYVRE031	0	1	1	1	1	1	2	1	1	1	1	1	1	1	1	1	1	17
SZYSC_ZDYVRE032	0	1	1	1	1	1	1	1	1	1	1	1	1	1	1	1	1	16
SZYSC_ZDYVRE030	0	0	1	1	1	1	2	1	1	1	1	1	1	1	1	1	1	16

Sample	*scm*	*PilA*	*Esp*	*sgrAc*	*PilB*	*acm*	*psaA*	*efaA*	FBP	*ecbA/fss3*	*plr/gapA*	*bopD*	*clpP*	*cpsA/uppS*	*cpsB/cdsA*	*eno*	*rfbB*	Total
SZYSC_ZDYVRE029	0	1	1	1	1	1	2	1	1	1	1	1	1	1	1	1	1	17
SZYSC_ZDYVRE028	0	1	1	1	1	1	2	1	1	1	1	1	1	1	1	1	1	17
SZYSC_ZDYVRE027	0	1	1	1	1	1	2	1	1	1	1	1	1	1	1	1	1	17
SZYSC_ZDYVRE023	0	1	1	1	1	1	2	1	1	1	1	1	1	1	1	1	1	17
SZYSC_ZDYVRE021	0	0	1	1	1	1	2	1	1	1	1	1	1	1	1	1	1	16
SZYSC_ZDYVRE017	0	1	1	1	1	1	2	1	1	1	1	1	1	1	1	1	1	17
SZYSC_ZDYVRE019	0	2	1	1	1	1	2	1	1	1	1	1	1	1	1	1	1	18
SZYSC_ZDYVRE018	0	2	1	1	1	1	2	1	1	1	1	1	1	1	1	1	1	18
SZYSC_ZDYVRE015	0	1	1	1	1	1	2	1	1	1	1	1	1	1	1	1	1	17
SZYSC_ZDYVRE012	0	1	1	1	1	1	2	1	1	1	1	1	1	1	1	1	1	17
SZYSC_ZDYVRE014	0	0	1	1	1	1	2	1	1	1	1	1	1	1	1	1	1	16
SZYSC_ZDYVRE011	0	1	1	1	1	1	2	1	1	1	1	1	1	1	1	1	1	17
SZYSC_ZDYVRE013	0	1	1	1	1	1	2	1	1	1	1	1	1	1	1	1	1	17
SZYSC_ZDYVRE010	0	1	1	1	1	1	2	1	1	1	1	1	1	1	1	1	1	17
SZYSC_ZDYVRE008	1	1	1	1	1	1	2	1	1	1	1	1	1	1	1	1	1	18
SZYSC_ZDYVRE009	0	1	1	1	1	1	2	1	1	1	1	1	1	1	1	1	1	17
SZYSC_ZDYVRE007	0	1	1	1	1	1	2	1	1	1	1	1	1	1	1	1	1	17
SZYSC_ZDYVRE006	0	1	1	1	1	1	2	1	1	1	1	1	1	1	1	1	1	17
SZYSC_ZDYVRE005	0	0	1	1	1	1	2	1	1	1	1	1	1	1	1	1	1	16
SZYSC_ZDYVRE004	0	0	1	1	1	1	2	1	1	1	1	1	1	1	1	1	1	16
SZYSC_ZDYVRE003	0	1	1	1	1	1	2	1	1	1	1	1	1	1	1	1	1	17
SZYSC_VRE006	0	1	1	1	1	1	2	1	1	1	1	1	1	1	1	1	1	17
SZYSC_VRE005	0	1	1	1	1	1	2	1	1	1	1	1	1	1	1	1	1	17
SZYSC_VRE001	1	1	1	1	1	1	2	1	1	1	1	1	1	1	1	1	1	18
SZYSC_SYVRE015	0	1	1	1	1	1	2	1	1	1	1	1	1	1	1	1	1	17
SZYSC_SYVRE006	0	1	1	1	1	1	2	1	1	1	1	1	1	1	1	1	1	17

Sample	*scm*	*PilA*	*Esp*	*sgrAc*	*PilB*	*acm*	*psaA*	*efaA*	FBP	*ecbA/fss3*	*plr/gapA*	*bopD*	*clpP*	*cpsA/uppS*	*cpsB/cdsA*	*eno*	*rfbB*	Total
SZYSC_SYVRE012	0	1	1	1	1	1	2	1	1	1	1	1	1	1	1	1	1	17
SZYSC_SCVRE013	0	1	1	1	1	1	2	1	1	1	1	1	1	1	1	1	1	17
SZYSC_SCVRE012	0	1	1	1	1	1	2	1	1	1	1	1	1	1	1	1	1	17
SZYSC_SYVRE001	0	1	1	1	1	1	2	1	1	1	1	1	1	1	1	1	1	17
SZYSC_SCVRE014	0	1	1	1	1	1	2	1	1	1	1	1	1	1	1	1	1	17
SZYSC_SCVRE011	0	1	1	1	1	1	2	1	1	1	1	1	1	1	1	1	1	17
SZYSC_SCVRE009	0	1	1	1	1	1	2	1	1	1	1	1	1	1	1	1	1	17
SZYSC_QYVRE003	0	1	1	1	1	1	1	1	1	1	1	1	1	1	1	1	1	16
SZYSC_QYVRE002	0	2	1	1	1	1	2	1	1	1	1	1	1	1	1	1	1	18
SZYSC_QYVRE001	0	1	1	1	1	1	2	1	1	1	1	1	1	1	1	1	1	17
SZYSC_QRYVRE002	0	1	1	1	1	1	2	1	1	1	1	1	1	1	1	1	1	17
SZYSC_QRYVRE001	0	1	1	1	1	1	2	1	1	1	1	1	1	1	1	1	1	17
SZYSC_PYVRE001	0	0	1	1	1	1	2	1	1	1	1	1	1	1	1	1	1	16
SZYSC_MZVRE013	0	1	1	1	1	1	1	1	1	1	1	1	1	1	1	1	1	16
SZYSC_MZVRE012	0	1	1	1	1	1	2	1	1	1	1	1	1	1	1	1	1	17
SZYSC_MZVRE010	0	1	1	1	1	1	2	1	1	1	1	1	1	1	1	1	1	17
SZYSC_MZVRE014	0	1	1	1	1	1	2	1	1	1	1	1	1	1	1	1	1	17
SZYSC_MZVRE011	0	1	1	1	1	1	2	1	1	1	1	1	1	1	1	1	1	17
SZYSC_MZVRE008	0	1	1	1	1	1	2	1	1	1	1	1	1	1	1	1	1	17
SZYSC_MZVRE009	0	1	1	1	1	1	2	1	1	1	1	1	1	1	1	1	1	17
SZYSC_MZVRE007	0	1	1	1	1	1	2	1	1	1	1	1	1	1	1	1	1	17
SZYSC_MZVRE006	0	1	1	1	1	1	2	1	1	1	1	1	1	1	1	1	1	17
SZYSC_MZVRE005	0	1	1	1	1	1	2	1	1	1	1	1	1	1	1	1	1	17
SZYSC_MZVRE004	0	1	1	1	1	1	2	1	1	1	1	1	1	1	1	1	1	17
SZYSC_MZVRE003	0	1	1	1	1	1	2	1	1	1	1	1	1	1	1	1	1	17
SZYSC_MZVRE002	0	1	1	1	1	1	1	1	1	1	1	1	1	1	1	1	1	16

Sample	*scm*	*PilA*	*Esp*	*sgrAc*	*PilB*	*acm*	*psaA*	*efaA*	FBP	*ecbA/fss3*	*plr/gapA*	*bopD*	*clpP*	*cpsA/uppS*	*cpsB/cdsA*	*eno*	*rfbB*	Total
SZYSC_LZVRE008	0	1	1	1	1	1	2	1	1	1	1	1	1	1	1	1	1	17
SZYSC_MZVRE001	0	1	1	1	1	1	2	1	1	1	1	1	1	1	1	1	1	17
SZYSC_LZVRE004	0	1	1	1	1	1	2	1	1	1	1	1	1	1	1	1	1	17
SZYSC_LZVRE002	0	1	1	1	1	1	2	1	1	1	1	1	1	1	1	1	1	17
SZYSC_LZVRE003	0	1	1	1	1	1	2	1	1	1	1	1	1	1	1	1	1	17
SZYSC_LZVRE001	0	1	1	1	1	1	2	1	1	1	1	1	1	1	1	1	1	17
SZYSC_GYSVRE004	0	1	1	1	1	1	2	1	1	1	1	1	1	1	1	1	1	17
SZYSC_GYSVRE003	1	1	1	1	1	1	2	1	1	1	1	1	1	1	1	1	1	18
SZYSC_GYSVRE001	0	1	1	1	1	1	2	1	1	1	1	1	1	1	1	1	1	17
SZYSC_FSYVRE024	0	1	1	1	1	1	2	2	1	1	1	1	1	1	1	1	1	18
SZYSC_FSYVRE025	0	1	1	1	1	1	2	1	1	1	1	1	1	1	1	1	1	17
SZYSC_FSYVRE023	0	1	1	1	1	1	2	1	1	1	1	1	1	1	1	1	1	17
SZYSC_FSYVRE022	0	1	1	1	1	1	2	1	1	1	1	1	1	1	1	1	1	17
SZYSC_FSYVRE021	0	1	1	1	1	1	2	1	1	1	1	1	1	1	1	1	1	17
SZYSC_FSYVRE020	0	1	1	1	1	1	2	1	1	1	1	1	1	1	1	1	1	17
SZYSC_FSYVRE019	0	1	1	1	1	1	2	1	1	1	1	1	1	1	1	1	1	17
SZYSC_FSYVRE018	0	1	1	1	1	1	2	1	1	1	1	1	1	1	1	1	1	17
SZYSC_FSYVRE017	0	1	1	1	1	1	2	1	1	1	1	1	1	1	1	1	1	17
SZYSC_FSYVRE016	0	1	1	1	1	1	2	1	1	1	1	1	1	1	1	1	1	17
SZYSC_FSYVRE015	0	1	1	1	1	1	2	1	1	1	1	1	1	1	1	1	1	17
SZYSC_FSYVRE012	0	1	1	1	1	1	2	1	1	1	1	1	1	1	1	1	1	17
SZYSC_FSYVRE013	0	1	1	1	1	1	2	1	1	1	1	1	1	1	1	1	1	17
SZYSC_FSYVRE014	0	1	1	1	1	1	2	1	1	1	1	1	1	1	1	1	1	17
SZYSC_FSYVRE011	0	1	1	1	1	1	2	1	1	1	1	1	1	1	1	1	1	17
SZYSC_FSYVRE010	0	1	1	1	1	1	2	1	1	1	1	1	1	1	1	1	1	17
SZYSC_FSYVRE009	0	1	1	1	1	1	2	1	1	1	1	1	1	1	1	1	1	17

Sample	*scm*	*PilA*	*Esp*	*sgrAc*	*PilB*	*acm*	*psaA*	*efaA*	FBP	*ecbA/fss3*	*plr/gapA*	*bopD*	*clpP*	*cpsA/uppS*	*cpsB/cdsA*	*eno*	*rfbB*	Total
SZYSC_FSYVRE006	0	1	1	1	1	1	2	1	1	1	1	1	1	1	1	1	1	17
SZYSC_FSYVRE007	0	1	1	1	1	1	2	1	1	1	1	1	1	1	1	1	1	17
SZYSC_FSYVRE008	0	2	1	1	1	1	2	1	1	1	1	1	1	1	1	1	1	18
SZYSC_FSYVRE005	0	1	1	1	1	1	2	1	1	1	1	1	1	1	1	1	1	17
SZYSC_FSYVRE004	0	1	1	1	1	1	2	1	1	1	1	1	1	1	1	1	1	17
SZYSC_FSYVRE002	0	1	1	1	1	1	2	1	1	1	1	1	1	1	1	1	1	17
SZYSC_FSVRE010	0	1	1	1	1	1	2	1	1	1	1	1	1	1	1	1	1	17
SZYSC_FSYVRE003	0	0	1	1	1	1	2	1	1	1	1	1	1	1	1	1	1	16
SZYSC_FSYVRE001	0	1	1	1	1	1	2	1	1	1	1	1	1	1	1	1	1	17
SZYSC_FSVRE009	0	1	1	1	1	1	2	1	1	1	1	1	1	1	1	1	1	17
SZYSC_FSVRE008	0	1	0	1	1	1	2	1	1	1	1	1	1	1	1	1	1	16
SZYSC_FSVRE005	0	1	1	1	1	1	2	1	1	1	1	1	1	1	1	1	1	17
SZYSC_FSVRE006	0	1	1	1	1	1	2	1	1	1	1	1	1	1	1	1	1	17
SZYSC_FSVRE007	0	1	1	1	1	1	2	1	1	1	1	1	1	1	1	1	1	17
SZYSC_FSVRE004	0	1	1	1	1	1	2	1	1	1	1	1	1	1	1	1	1	17
SZYSC_FSVRE002	0	1	1	1	1	1	2	1	1	1	1	1	1	1	1	1	1	17
SZYSC_FSVRE001	0	0	1	1	1	1	2	1	1	1	1	1	1	1	1	1	1	16
SZYSC_E025	0	1	1	1	1	1	2	1	1	1	1	1	1	1	1	1	1	17
SZYSC_E024	0	2	1	1	1	1	2	1	1	1	1	1	1	1	1	1	1	18
SZYSC_E023	0	1	1	1	1	1	2	1	1	1	1	1	1	1	1	1	1	17
SZYSC_E022	0	2	1	1	1	1	2	1	1	1	1	1	1	1	1	1	1	18
SZYSC_E019	0	1	1	1	1	1	2	1	1	1	1	1	1	1	1	1	1	17
SZYSC_E020	0	1	1	1	1	1	2	1	1	1	1	1	1	1	1	1	1	17
SZYSC_E018	0	1	1	1	1	1	2	1	1	1	1	1	1	1	1	1	1	17
SZYSC_E013	0	2	1	1	1	1	2	1	1	1	1	1	1	1	1	1	1	18
SZYSC_E012	0	2	1	1	1	1	2	1	1	1	1	1	1	1	1	1	1	18

Sample	*scm*	*PilA*	*Esp*	*sgrAc*	*PilB*	*acm*	*psaA*	*efaA*	FBP	*ecbA/fss3*	*plr/gapA*	*bopD*	*clpP*	*cpsA/uppS*	*cpsB/cdsA*	*eno*	*rfbB*	Total
SZYSC_E010	0	0	1	1	1	1	2	1	1	1	1	1	1	1	1	1	1	16
SZYSC_DYVRE030	1	1	1	1	1	1	2	1	1	1	1	1	1	1	1	1	1	18
SZYSC_DYVRE029	1	1	1	1	1	1	2	1	1	1	1	1	1	1	1	1	1	18
SZYSC_DYVRE028	0	1	1	1	1	1	2	1	1	1	1	1	1	1	1	1	1	17
SZYSC_DYVRE027	0	0	1	1	1	1	2	1	1	1	1	1	1	1	1	1	1	16
SZYSC_DYVRE025	0	1	1	1	1	1	2	1	1	1	1	1	1	1	1	1	1	17
SZYSC_DYVRE026	0	0	1	1	1	1	2	1	1	1	1	1	1	1	1	1	1	16
SZYSC_DYVRE023	0	0	1	1	1	1	2	1	1	1	1	1	1	1	1	1	1	16
SZYSC_DYVRE024	0	1	1	1	1	1	2	1	1	1	1	1	1	1	1	1	1	17
SZYSC_DYVRE022	0	1	1	1	1	1	2	1	1	1	1	1	1	1	1	1	1	17
SZYSC_DYVRE020	0	1	1	1	1	1	2	1	1	1	1	1	1	1	1	1	1	17
SZYSC_DYVRE018	0	0	1	1	1	1	2	1	1	1	1	1	1	1	1	1	1	16
SZYSC_DYVRE019	0	0	1	1	1	1	2	1	1	1	1	1	1	1	1	1	1	16
SZYSC_DYVRE017	0	0	1	1	1	1	2	1	1	1	1	1	1	1	1	1	1	16
SZYSC_DYVRE015	0	0	1	1	1	1	2	1	1	1	1	1	1	1	1	1	1	16
SZYSC_DYVRE014	0	1	1	1	1	1	2	1	1	1	1	1	1	1	1	1	1	17
SZYSC_DYVRE016	0	0	1	1	1	1	2	1	1	1	1	1	1	1	1	1	1	16
SZYSC_DYVRE013	0	1	1	1	1	1	2	1	1	1	1	1	1	1	1	1	1	17
SZYSC_DYVRE012	0	1	1	1	1	1	2	1	1	1	1	1	1	1	1	1	1	17
SZYSC_DYVRE011	0	1	1	1	1	1	2	1	1	1	1	1	1	1	1	1	1	17
SZYSC_DYVRE010	0	1	1	1	1	1	2	1	1	1	1	1	1	1	1	1	1	17
SZYSC_DYVRE009	0	0	1	1	1	1	2	1	1	1	1	1	1	1	1	1	1	16
SZYSC_DYVRE008	0	0	1	1	1	1	2	1	1	1	1	1	1	1	1	1	1	16
SZYSC_DYVRE007	0	1	1	1	1	1	2	1	1	1	1	1	1	1	1	1	1	17
SZYSC_DYVRE006	0	0	1	1	1	1	2	1	1	1	1	1	1	1	1	1	1	16
SZYSC_DYVRE005	0	1	1	1	1	1	2	1	1	1	1	1	1	1	1	1	1	17

Sample	*scm*	*PilA*	*Esp*	*sgrAc*	*PilB*	*acm*	*psaA*	*efaA*	FBP	*ecbA/fss3*	*plr/gapA*	*bopD*	*clpP*	*cpsA/uppS*	*cpsB/cdsA*	*eno*	*rfbB*	Total
SZYSC_DYVRE004	0	1	0	1	1	1	2	1	1	1	1	1	1	1	1	1	1	16
SZYSC_DYVRE003	0	1	1	1	1	1	2	1	1	1	1	1	1	1	1	1	1	17
SZYSC_DYVRE002	0	1	1	1	1	1	2	1	1	1	1	1	1	1	1	1	1	17
SZYSC_DHVRE001	0	1	1	1	1	1	2	1	1	1	1	1	1	1	1	1	1	17
SZYSC_23VRE019	1	0	1	1	1	1	1	1	1	1	1	1	1	1	1	1	1	16
SZYSC_23VRE017	0	1	1	1	1	1	2	1	1	1	1	1	1	1	1	1	1	17
SZYSC_23VRE018	0	0	1	1	1	1	2	1	1	1	1	1	1	1	1	1	1	16
SZYSC_23VRE016	0	1	1	1	1	1	2	1	1	1	1	1	1	1	1	1	1	17
SZYSC_23VRE015	0	0	1	1	1	1	2	1	1	1	1	1	1	1	1	1	1	16
SZYSC_23VRE014	0	1	1	1	1	1	2	1	1	1	1	1	1	1	1	1	1	17
SZYSC_23VRE013	0	1	1	1	1	1	2	1	1	1	1	1	1	1	1	1	1	17
SZYSC_23VRE012	0	1	1	1	1	1	2	1	1	1	1	1	1	1	1	1	1	17
SZYSC_23VRE011	0	0	1	1	1	1	2	1	1	1	1	1	1	1	1	1	1	16
SZYSC_23VRE009	0	1	1	1	1	1	2	1	1	1	1	1	1	1	1	1	1	17
SZYSC_23VRE010	1	1	1	1	1	1	2	1	1	1	1	1	1	1	1	1	1	18
SZYSC_23VRE007	0	1	1	1	1	1	2	1	1	1	1	1	1	1	1	1	1	17
SZYSC_23VRE004	0	1	1	1	1	1	1	1	1	1	1	1	1	1	1	1	1	16
SZYSC_23VRE002	0	1	1	1	1	1	2	1	1	1	1	1	1	1	1	1	1	17
SZYSC_23VRE020	0	0	1	1	1	1	2	1	1	1	1	1	1	1	1	1	1	16
SZYSC_22VRE32	0	1	1	1	1	1	2	1	1	1	1	1	1	1	1	1	1	17
SZYSC_22VRE31	0	1	1	1	1	1	2	1	1	1	1	1	1	1	1	1	1	17
SZYSC_21VRE007	0	1	1	1	1	1	2	1	1	1	1	1	1	1	1	1	1	17
SZYSC_22VRE30	0	1	0	0	1	1	2	1	1	1	1	1	1	1	1	1	1	15
SZYSC_21VRE003	0	1	1	1	1	1	2	1	1	1	1	1	1	1	1	1	1	17
VRE_LHCMP_001	1	1	1	1	1	1	2	1	1	1	1	1	1	1	1	1	1	18
VRE_LHCMP_002	1	1	1	1	1	1	2	1	1	1	1	1	1	1	1	1	1	18

Sample	*scm*	*PilA*	*Esp*	*sgrAc*	*PilB*	*acm*	*psaA*	*efaA*	FBP	*ecbA/fss3*	*plr/gapA*	*bopD*	*clpP*	*cpsA/uppS*	*cpsB/cdsA*	*eno*	*rfbB*	Total
VRE_LHCMP_003	1	1	1	1	1	1	2	1	1	1	1	1	1	1	1	1	1	18
VRE_LHCMP_004	1	1	1	1	1	1	2	1	1	1	1	1	1	1	1	1	1	18

*FBP, fibronectin-binding protein.

Appendix 1 Table 4. Gene contents and proportion of Recomb1–3 carrying SC11 isolates

	Proportion			
Gene	SC11-pop I	SC11-pop II	Recomb modular	Gene annotation
group_437	0.6176	0.0348	Recomb1	hypothetical_protein
group_2058	0.5588	0.0000	Recomb1	hypothetical_protein
group_2059	0.6176	0.0000	Recomb1	hypothetical_protein
group_2060	0.6176	0.0000	Recomb1	hypothetical_protein
group_2061	0.6176	0.0000	Recomb1	hypothetical_protein
group_2062	0.6176	0.0000	Recomb1	hypothetical_protein
group_2064	0.6176	0.0000	Recomb1	hypothetical_protein
group_2063	0.6176	0.0000	Recomb1	hypothetical_protein
group_1184	0.7353	0.0000	Recomb1	hypothetical_protein
group_143	0.7647	0.0000	Recomb1	hypothetical_protein
group_849	0.7647	0.0000	Recomb1	hypothetical_protein
group_961	0.7647	0.0000	Recomb1	hypothetical_protein
group_992	0.7647	0.0000	Recomb1	hypothetical_protein
group_1052	0.7647	0.0000	Recomb1	hypothetical_protein
group_1135	0.7647	0.0000	Recomb1	hypothetical_protein
group_1195	0.7647	0.0000	Recomb1	hypothetical_protein
group_1222	0.7647	0.0000	Recomb1	hypothetical_protein
group_1245	0.7647	0.0000	Recomb1	hypothetical_protein
group_1292	0.7647	0.0000	Recomb1	hypothetical_protein
group_1278	0.7647	0.0000	Recomb1	hypothetical_protein
group_620	0.6765	0.0000	Recomb1	ISL3_family_transposase_ISEfa11
levE	0.7059	0.0050	Recomb1	PTS_system_fructose-specific_EIIB_component
group_213	0.7059	0.0050	Recomb1	hypothetical_protein
bga	0.7059	0.0000	Recomb1	Beta-galactosidase
pepC_1	0.7059	0.0000	Recomb1	Aminopeptidase_C
agaS	0.7059	0.0000	Recomb1	D-galactosamine-6-phosphate_deaminase_AgaS
dap	0.7059	0.0000	Recomb1	D-aminopeptidase
group_151	0.7059	0.0000	Recomb1	hypothetical_protein
lacD2_1	0.7059	0.0000	Recomb1	Tagatose_1%2C6-diphosphate_aldolase_2
ansB	0.7059	0.0000	Recomb1	L-asparaginase
group_173	0.7059	0.0000	Recomb1	hypothetical_protein
lacC_1	0.7059	0.0000	Recomb1	Tagatose-6-phosphate_kinase
ycsE	0.7059	0.0000	Recomb1	5-amino-6-(5-phospho-D-ribitylamino)uracil_phosphatase_YcsE
group_255	0.7059	0.0000	Recomb1	putative_ketoamine_kinase
group_258	0.7059	0.0000	Recomb1	hypothetical_protein
group_259	0.7059	0.0000	Recomb1	hypothetical_protein
group_275	0.7059	0.0000	Recomb1	PTS_system_mannose-specific_EIID_component
iphP	0.7059	0.0000	Recomb1	Tyrosine-protein_phosphatase
group_320	0.7059	0.0000	Recomb1	hypothetical_protein
nagR_2	0.7059	0.0000	Recomb1	HTH-type_transcriptional_repressor_NagR

Gene	Proportion SC11-pop I	SC11-pop II	Recomb modular	Gene annotation
frlR	0.7059	0.0000	Recomb1	putative_fructoselysine_utilization_operon_transcriptional_repressor
group_435	0.7059	0.0000	Recomb1	hypothetical_protein
bcrR	0.7059	0.0000	Recomb1	HTH-type_transcriptional_activator_BcrR
group_676	0.7059	0.0000	Recomb1	hypothetical_protein
group_948	0.7059	0.0000	Recomb1	hypothetical_protein
group_1043	0.7059	0.0000	Recomb1	hypothetical_protein
group_1073	0.7059	0.0000	Recomb1	hypothetical_protein
group_1121	0.7059	0.0000	Recomb1	hypothetical_protein
group_1122	0.7059	0.0000	Recomb1	hypothetical_protein
group_1206	0.7059	0.0000	Recomb1	hypothetical_protein
group_1286	0.7059	0.0000	Recomb1	hypothetical_protein
group_1264	0.7059	0.0000	Recomb1	hypothetical_protein
group_123	0.8529	0.0000	Recomb1	hypothetical_protein
group_439	0.9118	0.0000	Recomb1	hypothetical_protein
group_282	0.9412	0.0000	Recomb1	hypothetical_protein
group_860	0.9412	0.0000	Recomb1	hypothetical_protein
group_829	0.9412	0.0000	Recomb1	hypothetical_protein
group_904	0.9118	0.0000	Recomb1	hypothetical_protein
group_971	0.9412	0.0050	Recomb1	hypothetical_protein
dinB_3_dinB_4	0.9412	0.0000	Recomb1	hypothetical_protein
group_1341	0.9412	0.0000	Recomb1	hypothetical_protein
group_845	0.9412	0.0000	Recomb1	hypothetical_protein
group_916	0.9706	0.0000	Recomb1	hypothetical_protein
group_913	0.9706	0.0000	Recomb1	hypothetical_protein
group_37	0.9706	0.0000	Recomb1	hypothetical_protein
group_930	0.9706	0.0000	Recomb1	hypothetical_protein
group_863	0.9706	0.0000	Recomb1	hypothetical_protein
group_630	1.0000	0.0050	Recomb1	hypothetical_protein
group_1010	1.0000	0.0050	Recomb1	hypothetical_protein
group_894	1.0000	0.0050	Recomb1	hypothetical_protein
group_106	1.0000	0.0000	Recomb1	hypothetical_protein
group_110	1.0000	0.0000	Recomb1	hypothetical_protein
group_138	1.0000	0.0000	Recomb1	hypothetical_protein
group_140	1.0000	0.0000	Recomb1	hypothetical_protein
group_153	1.0000	0.0000	Recomb1	hypothetical_protein
group_209	1.0000	0.0000	Recomb1	hypothetical_protein
group_240	1.0000	0.0000	Recomb1	hypothetical_protein
group_241	1.0000	0.0000	Recomb1	hypothetical_protein
group_270	1.0000	0.0000	Recomb1	hypothetical_protein
group_279	1.0000	0.0000	Recomb1	hypothetical_protein
group_284	1.0000	0.0000	Recomb1	hypothetical_protein

Gene	Proportion		Recomb modular	Gene annotation
	SC11-pop I	SC11-pop II		
group_287	1.0000	0.0000	Recomb1	hypothetical_protein
group_492	1.0000	0.0000	Recomb1	hypothetical_protein
group_517	1.0000	0.0000	Recomb1	hypothetical_protein
group_538	1.0000	0.0000	Recomb1	hypothetical_protein
group_544	1.0000	0.0000	Recomb1	hypothetical_protein
group_580	1.0000	0.0000	Recomb1	hypothetical_protein
group_589	1.0000	0.0000	Recomb1	hypothetical_protein
group_619	1.0000	0.0000	Recomb1	hypothetical_protein
group_621	1.0000	0.0000	Recomb1	hypothetical_protein
group_633	1.0000	0.0000	Recomb1	hypothetical_protein
group_643	1.0000	0.0000	Recomb1	hypothetical_protein
group_650	1.0000	0.0000	Recomb1	hypothetical_protein
group_662	1.0000	0.0000	Recomb1	hypothetical_protein
group_665	1.0000	0.0000	Recomb1	hypothetical_protein
group_735	1.0000	0.0000	Recomb1	hypothetical_protein
group_750	1.0000	0.0000	Recomb1	hypothetical_protein
group_752	1.0000	0.0000	Recomb1	hypothetical_protein
group_757	1.0000	0.0000	Recomb1	hypothetical_protein
group_758	1.0000	0.0000	Recomb1	hypothetical_protein
group_776	1.0000	0.0000	Recomb1	hypothetical_protein
group_779	1.0000	0.0000	Recomb1	hypothetical_protein
group_780	1.0000	0.0000	Recomb1	hypothetical_protein
group_786	1.0000	0.0000	Recomb1	hypothetical_protein
group_790	1.0000	0.0000	Recomb1	hypothetical_protein
group_798	1.0000	0.0000	Recomb1	hypothetical_protein
group_809	1.0000	0.0000	Recomb1	hypothetical_protein
group_815	1.0000	0.0000	Recomb1	hypothetical_protein
group_816	1.0000	0.0000	Recomb1	hypothetical_protein
group_837	1.0000	0.0000	Recomb1	hypothetical_protein
group_839	1.0000	0.0000	Recomb1	hypothetical_protein
group_840	1.0000	0.0000	Recomb1	hypothetical_protein
group_881	1.0000	0.0000	Recomb1	hypothetical_protein
group_882	1.0000	0.0000	Recomb1	hypothetical_protein
group_883	1.0000	0.0000	Recomb1	hypothetical_protein
group_897	1.0000	0.0000	Recomb1	hypothetical_protein
group_900	1.0000	0.0000	Recomb1	hypothetical_protein
group_901	1.0000	0.0000	Recomb1	hypothetical_protein
group_903	1.0000	0.0000	Recomb1	hypothetical_protein
group_910	1.0000	0.0000	Recomb1	hypothetical_protein
group_912	1.0000	0.0000	Recomb1	hypothetical_protein
group_914	1.0000	0.0000	Recomb1	hypothetical_protein

	Proportion			
	SC11-pop I	SC11-pop II		
Gene			Recomb modular	Gene annotation
group_917	1.0000	0.0000	Recomb1	hypothetical_protein
group_918	1.0000	0.0000	Recomb1	hypothetical_protein
group_919	1.0000	0.0000	Recomb1	hypothetical_protein
group_920	1.0000	0.0000	Recomb1	hypothetical_protein
group_921	1.0000	0.0000	Recomb1	hypothetical_protein
group_922	1.0000	0.0000	Recomb1	hypothetical_protein
group_925	1.0000	0.0000	Recomb1	hypothetical_protein
group_931	1.0000	0.0000	Recomb1	hypothetical_protein
group_938	1.0000	0.0000	Recomb1	hypothetical_protein
group_953	1.0000	0.0000	Recomb1	hypothetical_protein
group_954	1.0000	0.0000	Recomb1	hypothetical_protein
group_956	1.0000	0.0000	Recomb1	hypothetical_protein
group_1015	1.0000	0.0000	Recomb1	hypothetical_protein
group_1017	1.0000	0.0000	Recomb1	hypothetical_protein
group_1123	1.0000	0.0000	Recomb1	hypothetical_protein
group_1201	1.0000	0.0000	Recomb1	hypothetical_protein
group_1223	1.0000	0.0000	Recomb1	hypothetical_protein
group_1229	1.0000	0.0000	Recomb1	hypothetical_protein
group_2065	1.0000	0.0000	Recomb1	hypothetical_protein
group_1283	1.0000	0.0000	Recomb1	hypothetical_protein
group_445	0.3529	0.0000	Recomb1	hypothetical_protein
group_440	0.4118	0.0000	Recomb1	hypothetical_protein
group_441	0.4118	0.0000	Recomb1	hypothetical_protein
group_693	0.4118	0.0000	Recomb1	hypothetical_protein
group_707	0.4118	0.0000	Recomb1	hypothetical_protein
group_879	0.4118	0.0000	Recomb1	hypothetical_protein
group_773	0.4118	0.0000	Recomb1	hypothetical_protein
group_1986	0.3235	0.0000	Recomb1	IS256_family_transposase_IS1542
group_1170	0.2353	0.0000	Recomb1	hypothetical_protein
group_1217	0.2059	0.0000	Recomb1	hypothetical_protein
group_1804	0.2941	0.3333	Recomb2	P-loop_guanosine_triphosphatase_YjiA
group_594	0.2059	0.1592	Recomb2	hypothetical_protein
group_854	0.2353	0.2090	Recomb2	hypothetical_protein
group_327	0.2353	0.1990	Recomb2	hypothetical_protein
group_887	0.2647	0.3134	Recomb2	hypothetical_protein
group_795	0.2353	0.2886	Recomb2	hypothetical_protein
group_762	0.2059	0.2935	Recomb2	hypothetical_protein
group_1244	0.2059	0.2935	Recomb2	hypothetical_protein
group_1240	0.2059	0.2935	Recomb2	hypothetical_protein
group_1243	0.2059	0.2935	Recomb2	hypothetical_protein
group_57	0.2353	0.2836	Recomb2	hypothetical_protein

	Proportion			
Gene	SC11-pop I	SC11-pop II	Recomb modular	Gene annotation
group_1131	0.2353	0.2935	Recomb2	hypothetical_protein
group_346	0.2353	0.2886	Recomb2	ATP-dependent_Clp_protease_proteolytic_subunit
group_592	0.2353	0.2886	Recomb2	hypothetical_protein
group_616	0.2353	0.2886	Recomb2	hypothetical_protein
group_767	0.2353	0.2886	Recomb2	hypothetical_protein
group_793	0.2353	0.2886	Recomb2	hypothetical_protein
group_998	0.2353	0.2886	Recomb2	hypothetical_protein
group_1168	0.2353	0.2886	Recomb2	hypothetical_protein
group_1139	0.2353	0.2886	Recomb2	hypothetical_protein
group_926	0.2647	0.2786	Recomb2	hypothetical_protein
group_1295	0.1765	0.2388	Recomb2	hypothetical_protein
group_421	0.1765	0.2388	Recomb2	hypothetical_protein
group_768	0.2059	0.2786	Recomb2	hypothetical_protein
group_792	0.2941	0.2587	Recomb2	hypothetical_protein
group_428	0.2059	0.3582	Recomb2	IS3_family_transposase_ISEfa8
group_415	0.0588	0.1940	Recomb2	hypothetical_protein
group_690	0.1176	0.2786	Recomb2	hypothetical_protein
group_392	0.1176	0.2786	Recomb2	hypothetical_protein
group_843	0.2059	0.3383	Recomb2	hypothetical_protein
group_864	0.2941	0.3731	Recomb2	hypothetical_protein
group_853	0.2647	0.4030	Recomb2	hypothetical_protein
group_1262	0.2941	0.4129	Recomb2	hypothetical_protein
group_842	0.2941	0.4129	Recomb2	hypothetical_protein
group_907	0.2941	0.4179	Recomb2	hypothetical_protein
group_772	0.2941	0.4229	Recomb2	hypothetical_protein
group_855	0.2941	0.4229	Recomb2	hypothetical_protein
group_810	0.2941	0.4229	Recomb2	hypothetical_protein
group_1210	0.2353	0.3930	Recomb2	hypothetical_protein
group_632	0.2941	0.3930	Recomb2	hypothetical_protein
group_702	0.2647	0.3881	Recomb2	hypothetical_protein
group_978	0.2353	0.3930	Recomb2	hypothetical_protein
xerD_2_xerC_7	0.2647	0.3930	Recomb2	Tyrosine_recombinase_XerC
group_812	0.2647	0.3980	Recomb2	hypothetical_protein
group_905	0.2647	0.3980	Recomb2	hypothetical_protein
group_1282	0.2647	0.3980	Recomb2	hypothetical_protein
group_1181	0.2647	0.3980	Recomb2	hypothetical_protein
group_886	0.2941	0.3831	Recomb2	hypothetical_protein
group_852	0.2941	0.3831	Recomb2	hypothetical_protein
group_734	0.2647	0.4080	Recomb2	hypothetical_protein
group_269	0.2941	0.4129	Recomb2	hypothetical_protein
group_760	0.2941	0.4129	Recomb2	hypothetical_protein

	Proportion			
	SC11-pop I	SC11-pop II		
Gene			Recomb modular	Gene annotation
group_826	0.2941	0.4129	Recomb2	hypothetical_protein
group_911	0.2941	0.4129	Recomb2	hypothetical_protein
group_892	0.2941	0.4129	Recomb2	hypothetical_protein
group_649	0.2941	0.4080	Recomb2	hypothetical_protein
group_828	0.2941	0.4080	Recomb2	hypothetical_protein
group_865	0.2941	0.4080	Recomb2	hypothetical_protein
group_895	0.2941	0.4080	Recomb2	hypothetical_protein
group_1089	0.2941	0.4080	Recomb2	hypothetical_protein
group_927	0.2941	0.4080	Recomb2	hypothetical_protein
group_867	0.2647	0.3930	Recomb2	hypothetical_protein
group_663	0.2647	0.4030	Recomb2	hypothetical_protein
group_1337	0.2647	0.4080	Recomb2	hypothetical_protein
group_272	0.2647	0.4080	Recomb2	hypothetical_protein
group_1952	0.0588	0.0896	Recomb3	hypothetical_protein
group_1642	0.1471	0.0945	Recomb3	hypothetical_protein
group_1656	0.1471	0.0945	Recomb3	hypothetical_protein
group_1666	0.1471	0.0945	Recomb3	hypothetical_protein
group_1669	0.1471	0.0945	Recomb3	hypothetical_protein
group_1671	0.1471	0.0945	Recomb3	hypothetical_protein
group_1688	0.1471	0.0945	Recomb3	hypothetical_protein
group_1697	0.1471	0.0945	Recomb3	hypothetical_protein
group_1739	0.1471	0.0945	Recomb3	hypothetical_protein
group_1720	0.1471	0.0945	Recomb3	hypothetical_protein
group_1659	0.1471	0.0995	Recomb3	hypothetical_protein
group_1613	0.1471	0.0995	Recomb3	hypothetical_protein
group_1748	0.1471	0.1194	Recomb3	hypothetical_protein
toxN	0.1471	0.1144	Recomb3	Endoribonuclease_ToxN
group_1617	0.1471	0.1144	Recomb3	hypothetical_protein
group_1618	0.1471	0.1144	Recomb3	hypothetical_protein
group_1619	0.1471	0.1144	Recomb3	hypothetical_protein
group_1620	0.1471	0.1144	Recomb3	hypothetical_protein
group_1623	0.1471	0.1144	Recomb3	hypothetical_protein
group_1628	0.1471	0.1144	Recomb3	hypothetical_protein
group_1631	0.1471	0.1144	Recomb3	hypothetical_protein
group_1633	0.1471	0.1144	Recomb3	hypothetical_protein
group_1634	0.1471	0.1144	Recomb3	hypothetical_protein
group_1637	0.1471	0.1144	Recomb3	hypothetical_protein
group_1639	0.1471	0.1144	Recomb3	hypothetical_protein
group_1640	0.1471	0.1144	Recomb3	hypothetical_protein
group_1641	0.1471	0.1144	Recomb3	hypothetical_protein
group_1643	0.1471	0.1144	Recomb3	hypothetical_protein

Gene	Proportion		Recomb modular	Gene annotation
	SC11-pop I	SC11-pop II		
group_1644	0.1471	0.1144	Recomb3	hypothetical_protein
group_1647	0.1471	0.1144	Recomb3	hypothetical_protein
group_1648	0.1471	0.1144	Recomb3	hypothetical_protein
group_1649	0.1471	0.1144	Recomb3	hypothetical_protein
group_1650	0.1471	0.1144	Recomb3	hypothetical_protein
group_1655	0.1471	0.1144	Recomb3	hypothetical_protein
group_1657	0.1471	0.1144	Recomb3	hypothetical_protein
group_1660	0.1471	0.1144	Recomb3	hypothetical_protein
group_1663	0.1471	0.1144	Recomb3	hypothetical_protein
group_1670	0.1471	0.1144	Recomb3	hypothetical_protein
topB_2	0.1471	0.1144	Recomb3	DNA_topoisomerase_3
group_1674	0.1471	0.1144	Recomb3	hypothetical_protein
group_1676	0.1471	0.1144	Recomb3	hypothetical_protein
group_1677	0.1471	0.1144	Recomb3	hypothetical_protein
group_1681	0.1471	0.1144	Recomb3	hypothetical_protein
group_1684	0.1471	0.1144	Recomb3	hypothetical_protein
group_1690	0.1471	0.1144	Recomb3	hypothetical_protein
group_1691	0.1471	0.1144	Recomb3	hypothetical_protein
group_1703	0.1471	0.1144	Recomb3	hypothetical_protein
group_1708	0.1471	0.1144	Recomb3	hypothetical_protein
group_1709	0.1471	0.1144	Recomb3	hypothetical_protein
group_1711	0.1471	0.1144	Recomb3	hypothetical_protein
group_1721	0.1471	0.1144	Recomb3	hypothetical_protein
group_1732	0.1471	0.1144	Recomb3	hypothetical_protein
group_1733	0.1471	0.1144	Recomb3	hypothetical_protein
group_1736	0.1471	0.1144	Recomb3	hypothetical_protein
group_1737	0.1471	0.1144	Recomb3	hypothetical_protein
group_1742	0.1471	0.1144	Recomb3	hypothetical_protein
group_1743	0.1471	0.1144	Recomb3	hypothetical_protein
group_1744	0.1471	0.1144	Recomb3	hypothetical_protein
group_1746	0.1471	0.1144	Recomb3	Sporulation_initiation_inhibitor_protein_Soj

Appendix 1 Table 5. Clinical characteristics differences among SC11-pop I and SC11-pop II

Category	SC11-pop I, n = 30	SC11-pop II, n = 7	Significance
Average days of hospitalization	58.8	98	Not significant
Age, y			
0–30	2 (6.67%)	1 (14.29%)	Not significant
30–60	13 (43.33%)	1 (14.29%)	Not significant
>60	15 (50.00%)	5 (71.43%)	Not significant
Sex			
F	13 (43.33%)	4 (57.14%)	Not significant
M	17 (56.67%)	3 (42.86%)	Not significant
Length of hospitalization, d			
0–50	18 (60.00%)	4 (57.14%)	Not significant
50–100	8 (26.67%)	1 (14.29%)	Not significant
>100	2 (6.67%)	2 (28.57%)	Not significant
Diseases			
Cardiovascular diseases	2 (6.67%)	2 (28.57%)	Not significant
Respiratory diseases	12 (40.00%)	4 (57.14%)	Not significant
Diabetes	10 (33.33%)	1 (14.29%)	Not significant
Hypertension	10 (33.33%)	3 (42.86%)	Not significant
Liver diseases	9 (30.00%)	1 (14.29%)	Not significant
Kidney diseases	4 (13.00%)	3 (42.86%)	Not significant
Others	24 (80.00%)	7 (100.00%)	Not significant
Prognosis			
Improvement	9 (30.00%)	3 (42.86%)	Not significant
Cured	8 (26.67%)	1 (14.29%)	Not significant
Not cured	0 (0.00%)	1 (14.29%)	*
Death	3 (10.00%)	0 (0.00%)	Not significant
Unknown	10 (33.33%)	2 (28.57%)	Not significant

*$p<0.05$, χ^2 test.

Appendix 1 Table 6. Accessory genes distribute significantly different in frequency between SC11-pop I and SC11-pop II*

Gene	Frequency SC11-pop I	Frequency SC11-pop II	Frequency difference of SC11 pop II – SC11-pop I	p adjusted	Recomb modular	Gene annotation
group_1015	0.000	0.971	0.971	5.3038E-39	Recomb1	Hypothetical protein
group_1017	0.000	0.971	0.971	5.3038E-39	Recomb1	Hypothetical protein
group_106	0.000	0.971	0.971	5.3038E-39	Recomb1	Hypothetical protein
group_110	0.000	0.971	0.971	5.3038E-39	Recomb1	Hypothetical protein
group_1123	0.000	0.971	0.971	5.3038E-39	Recomb1	Hypothetical protein
group_1201	0.000	0.971	0.971	5.3038E-39	Recomb1	Hypothetical protein
group_1223	0.000	0.971	0.971	5.3038E-39	Recomb1	Hypothetical protein
group_1229	0.000	0.971	0.971	5.3038E-39	Recomb1	Hypothetical protein
group_1283	0.000	0.971	0.971	5.3038E-39	Recomb1	Hypothetical protein

Gene	Frequency SC11-pop I	Frequency SC11-pop II	Frequency difference of SC11 pop II – SC11-pop I	p adjusted	Recomb modular	Gene annotation
group_138	0.000	0.971	0.971	5.3038E-39	Recomb1	Hypothetical protein
group_140	0.000	0.971	0.971	5.3038E-39	Recomb1	Hypothetical protein
group_153	0.000	0.971	0.971	5.3038E-39	Recomb1	Hypothetical protein
group_2065	0.000	0.971	0.971	5.3038E-39	Recomb1	Hypothetical protein
group_209	0.000	0.971	0.971	5.3038E-39	Recomb1	Hypothetical protein
group_240	0.000	0.971	0.971	5.3038E-39	Recomb1	Hypothetical protein
group_241	0.000	0.971	0.971	5.3038E-39	Recomb1	Hypothetical protein
group_270	0.000	0.971	0.971	5.3038E-39	Recomb1	Hypothetical protein
group_279	0.000	0.971	0.971	5.3038E-39	Recomb1	Hypothetical protein
group_284	0.000	0.971	0.971	5.3038E-39	Recomb1	Hypothetical protein
group_287	0.000	0.971	0.971	5.3038E-39	Recomb1	Hypothetical protein
group_492	0.000	0.971	0.971	5.3038E-39	Recomb1	Hypothetical protein
group_517	0.000	0.971	0.971	5.3038E-39	Recomb1	Hypothetical protein
group_538	0.000	0.971	0.971	5.3038E-39	Recomb1	Hypothetical protein
group_544	0.000	0.971	0.971	5.3038E-39	Recomb1	Hypothetical protein
group_580	0.000	0.971	0.971	5.3038E-39	Recomb1	Hypothetical protein
group_589	0.000	0.971	0.971	5.3038E-39	Recomb1	Hypothetical protein
group_619	0.000	0.971	0.971	5.3038E-39	Recomb1	Hypothetical protein
group_621	0.000	0.971	0.971	5.3038E-39	Recomb1	Hypothetical protein
group_633	0.000	0.971	0.971	5.3038E-39	Recomb1	Hypothetical protein
group_643	0.000	0.971	0.971	5.3038E-39	Recomb1	Hypothetical protein
group_650	0.000	0.971	0.971	5.3038E-39	Recomb1	Hypothetical protein
group_662	0.000	0.971	0.971	5.3038E-39	Recomb1	Hypothetical protein
group_665	0.000	0.971	0.971	5.3038E-39	Recomb1	Hypothetical protein
group_735	0.000	0.971	0.971	5.3038E-39	Recomb1	Hypothetical protein
group_750	0.000	0.971	0.971	5.3038E-39	Recomb1	Hypothetical protein
group_752	0.000	0.971	0.971	5.3038E-39	Recomb1	Hypothetical protein
group_757	0.000	0.971	0.971	5.3038E-39	Recomb1	Hypothetical protein
group_758	0.000	0.971	0.971	5.3038E-39	Recomb1	Hypothetical protein
group_776	0.000	0.971	0.971	5.3038E-39	Recomb1	Hypothetical protein
group_779	0.000	0.971	0.971	5.3038E-39	Recomb1	Hypothetical protein
group_780	0.000	0.971	0.971	5.3038E-39	Recomb1	Hypothetical protein
group_786	0.000	0.971	0.971	5.3038E-39	Recomb1	Hypothetical protein
group_790	0.000	0.971	0.971	5.3038E-39	Recomb1	Hypothetical protein
group_798	0.000	0.971	0.971	5.3038E-39	Recomb1	Hypothetical protein
group_809	0.000	0.971	0.971	5.3038E-39	Recomb1	Hypothetical protein
group_815	0.000	0.971	0.971	5.3038E-39	Recomb1	Hypothetical protein
group_816	0.000	0.971	0.971	5.3038E-39	Recomb1	Hypothetical protein
group_837	0.000	0.971	0.971	5.3038E-39	Recomb1	Hypothetical protein
group_839	0.000	0.971	0.971	5.3038E-39	Recomb1	Hypothetical protein
group_840	0.000	0.971	0.971	5.3038E-39	Recomb1	Hypothetical protein

Gene	Frequency SC11-pop I	Frequency SC11-pop II	Frequency difference of SC11 pop II – SC11-pop I	p adjusted	Recomb modular	Gene annotation
group_881	0.000	0.971	0.971	5.3038E-39	Recomb1	Hypothetical protein
group_882	0.000	0.971	0.971	5.3038E-39	Recomb1	Hypothetical protein
group_883	0.000	0.971	0.971	5.3038E-39	Recomb1	Hypothetical protein
group_897	0.000	0.971	0.971	5.3038E-39	Recomb1	Hypothetical protein
group_900	0.000	0.971	0.971	5.3038E-39	Recomb1	Hypothetical protein
group_901	0.000	0.971	0.971	5.3038E-39	Recomb1	Hypothetical protein
group_903	0.000	0.971	0.971	5.3038E-39	Recomb1	Hypothetical protein
group_910	0.000	0.971	0.971	5.3038E-39	Recomb1	Hypothetical protein
group_912	0.000	0.971	0.971	5.3038E-39	Recomb1	Hypothetical protein
group_914	0.000	0.971	0.971	5.3038E-39	Recomb1	Hypothetical protein
group_917	0.000	0.971	0.971	5.3038E-39	Recomb1	Hypothetical protein
group_918	0.000	0.971	0.971	5.3038E-39	Recomb1	Hypothetical protein
group_919	0.000	0.971	0.971	5.3038E-39	Recomb1	Hypothetical protein
group_920	0.000	0.971	0.971	5.3038E-39	Recomb1	Hypothetical protein
group_921	0.000	0.971	0.971	5.3038E-39	Recomb1	Hypothetical protein
group_922	0.000	0.971	0.971	5.3038E-39	Recomb1	Hypothetical protein
group_925	0.000	0.971	0.971	5.3038E-39	Recomb1	Hypothetical protein
group_931	0.000	0.971	0.971	5.3038E-39	Recomb1	Hypothetical protein
group_938	0.000	0.971	0.971	5.3038E-39	Recomb1	Hypothetical protein
group_953	0.000	0.971	0.971	5.3038E-39	Recomb1	Hypothetical protein
group_954	0.000	0.971	0.971	5.3038E-39	Recomb1	Hypothetical protein
group_956	0.000	0.971	0.971	5.3038E-39	Recomb1	Hypothetical protein
group_1010	0.005	0.971	0.966	1.7735E-37	Recomb1	Hypothetical protein
group_630	0.005	0.971	0.966	1.7735E-37	Recomb1	Hypothetical protein
group_894	0.005	0.971	0.966	1.7735E-37	Recomb1	Hypothetical protein
group_37	0.000	0.943	0.943	4.8211E-37	Recomb1	Hypothetical protein
group_863	0.000	0.943	0.943	4.8211E-37	Recomb1	Hypothetical protein
group_913	0.000	0.943	0.943	4.8211E-37	Recomb1	Hypothetical protein
group_916	0.000	0.943	0.943	4.8211E-37	Recomb1	Hypothetical protein
group_930	0.000	0.943	0.943	4.8211E-37	Recomb1	Hypothetical protein
dinB_3---dinB_4	0.000	0.914	0.914	3.0347E-35	None	DNA polymerase IV
group_1341	0.000	0.914	0.914	3.0347E-35	Recomb1	Hypothetical protein
group_282	0.000	0.914	0.914	3.0347E-35	Recomb1	Hypothetical protein
group_829	0.000	0.914	0.914	3.0347E-35	Recomb1	Hypothetical protein
group_845	0.000	0.914	0.914	3.0347E-35	Recomb1	Hypothetical protein
group_860	0.000	0.914	0.914	3.0347E-35	Recomb1	Hypothetical protein
group_971	0.005	0.914	0.909	9.7575E-34	Recomb1	Hypothetical protein
group_439	0.000	0.886	0.886	1.4955E-33	Recomb1	Hypothetical protein
group_904	0.000	0.886	0.886	1.4955E-33	Recomb1	Hypothetical protein
group_123	0.000	0.829	0.829	2.0818E-30	Recomb1	Hypothetical protein
group_1052	0.000	0.743	0.743	3.3122E-26	Recomb1	Hypothetical protein

Gene	Frequency SC11-pop I	Frequency SC11-pop II	Frequency difference of SC11 pop II – SC11-pop I	p adjusted	Recomb modular	Gene annotation
group_1135	0.000	0.743	0.743	3.3122E-26	Recomb1	Hypothetical protein
group_1195	0.000	0.743	0.743	3.3122E-26	Recomb1	Hypothetical protein
group_1222	0.000	0.743	0.743	3.3122E-26	Recomb1	Hypothetical protein
group_1245	0.000	0.743	0.743	3.3122E-26	Recomb1	Hypothetical protein
group_1278	0.000	0.743	0.743	3.3122E-26	Recomb1	Hypothetical protein
group_1292	0.000	0.743	0.743	3.3122E-26	Recomb1	Hypothetical protein
group_143	0.000	0.743	0.743	3.3122E-26	Recomb1	Hypothetical protein
group_849	0.000	0.743	0.743	3.3122E-26	Recomb1	Hypothetical protein
group_961	0.000	0.743	0.743	3.3122E-26	Recomb1	Hypothetical protein
group_992	0.000	0.743	0.743	3.3122E-26	Recomb1	Hypothetical protein
group_1184	0.000	0.714	0.714	6.8873E-25	Recomb1	Hypothetical protein
ycsE	0.000	0.686	0.686	1.0287E-23	Recomb1	5-amino-6-(5-phospho-D-ribitylamino)uracil phosphatase YcsE
pepC_1	0.000	0.686	0.686	1.0287E-23	Recomb1	Aminopeptidase C
bga	0.000	0.686	0.686	1.0287E-23	Recomb1	Beta-galactosidase
dap	0.000	0.686	0.686	1.0287E-23	Recomb1	D-aminopeptidase
agaS	0.000	0.686	0.686	1.0287E-23	Recomb1	D-galactosamine-6-phosphate deaminase AgaS
bcrR	0.000	0.686	0.686	1.0287E-23	Recomb1	HTH-type transcriptional activator BcrR
nagR_2	0.000	0.686	0.686	1.0287E-23	Recomb1	HTH-type transcriptional repressor NagR
group_1043	0.000	0.686	0.686	1.0287E-23	Recomb1	Hypothetical protein
group_1073	0.000	0.686	0.686	1.0287E-23	Recomb1	Hypothetical protein
group_1121	0.000	0.686	0.686	1.0287E-23	Recomb1	Hypothetical protein
group_1122	0.000	0.686	0.686	1.0287E-23	Recomb1	Hypothetical protein
group_1206	0.000	0.686	0.686	1.0287E-23	Recomb1	Hypothetical protein
group_1264	0.000	0.686	0.686	1.0287E-23	Recomb1	Hypothetical protein
group_1286	0.000	0.686	0.686	1.0287E-23	Recomb1	Hypothetical protein
group_151	0.000	0.686	0.686	1.0287E-23	Recomb1	Hypothetical protein
group_173	0.000	0.686	0.686	1.0287E-23	Recomb1	Hypothetical protein
group_258	0.000	0.686	0.686	1.0287E-23	Recomb1	Hypothetical protein
group_259	0.000	0.686	0.686	1.0287E-23	Recomb1	Hypothetical protein
group_320	0.000	0.686	0.686	1.0287E-23	Recomb1	Hypothetical protein
group_435	0.000	0.686	0.686	1.0287E-23	Recomb1	Hypothetical protein
group_676	0.000	0.686	0.686	1.0287E-23	Recomb1	Hypothetical protein
group_948	0.000	0.686	0.686	1.0287E-23	Recomb1	Hypothetical protein
ansB	0.000	0.686	0.686	1.0287E-23	Recomb1	L-asparaginase
group_275	0.000	0.686	0.686	1.0287E-23	Recomb1	PTS system mannose-specific EIID component

Gene	Frequency SC11-pop I	Frequency SC11-pop II	Frequency difference of SC11 pop II – SC11-pop I	p adjusted	Recomb modular	Gene annotation
frlR	0.000	0.686	0.686	1.0287E-23	Recomb1	putative fructoselysine utilization operon transcriptional repressor
group_255	0.000	0.686	0.686	1.0287E-23	Recomb1	putative ketoamine kinase
lacD2_1	0.000	0.686	0.686	1.0287E-23	Recomb1	Tagatose 16-diphosphate aldolase 2
lacC_1	0.000	0.686	0.686	1.0287E-23	Recomb1	Tagatose-6-phosphate kinase
iphP	0.000	0.686	0.686	1.0287E-23	Recomb1	Tyrosine-protein phosphatase
group_213	0.005	0.686	0.681	2.3882E-22	Recomb1	Hypothetical protein
levE	0.005	0.686	0.681	2.3882E-22	Recomb1	PTS system fructose-specific EIIB component
group_620	0.000	0.657	0.657	1.8035E-22	Recomb1	ISL3 family transposase ISEfa11
group_2059	0.000	0.600	0.600	4.2588E-20	Recomb1	Hypothetical protein
group_2060	0.000	0.600	0.600	4.2588E-20	Recomb1	Hypothetical protein
group_2061	0.000	0.600	0.600	4.2588E-20	Recomb1	Hypothetical protein
group_2062	0.000	0.600	0.600	4.2588E-20	Recomb1	Hypothetical protein
group_2063	0.000	0.600	0.600	4.2588E-20	Recomb1	Hypothetical protein
group_2064	0.000	0.600	0.600	4.2588E-20	Recomb1	Hypothetical protein
group_437	0.035	0.600	0.565	3.1463E-14	Recomb1	Hypothetical protein
group_2058	0.000	0.543	0.543	8.1824E-18	Recomb1	Hypothetical protein
group_440	0.000	0.400	0.400	1.608E-12	Recomb1	Hypothetical protein
group_441	0.000	0.400	0.400	1.608E-12	Recomb1	Hypothetical protein
group_693	0.000	0.400	0.400	1.608E-12	Recomb1	Hypothetical protein
group_707	0.000	0.400	0.400	1.608E-12	Recomb1	Hypothetical protein
group_773	0.000	0.400	0.400	1.608E-12	Recomb1	Hypothetical protein
group_879	0.000	0.400	0.400	1.608E-12	Recomb1	Hypothetical protein
group_445	0.000	0.343	0.343	1.5627E-10	Recomb1	Hypothetical protein
group_1986	0.000	0.314	0.314	1.4488E-09	Recomb1	IS256 family transposase IS1542
group_1170	0.000	0.229	0.229	9.4658E-07	Recomb1	Hypothetical protein
group_1217	0.000	0.200	0.200	7.6571E-06	Recomb1	Hypothetical protein
group_2068	0.220	0.514	0.294	0.0052	None	Hypothetical protein
group_2076	0.205	0.486	0.281	0.0080	None	Hypothetical protein
group_2075	0.220	0.486	0.266	0.0205	None	Hypothetical protein
group_2066	0.205	0.457	0.252	0.0185	None	Hypothetical protein
group_2070	0.235	0.486	0.251	0.0264	None	Hypothetical protein
group_2069	0.225	0.457	0.232	0.0441	None	Hypothetical protein
group_1911	0.055	0.257	0.202	0.0054	None	Hypothetical protein
group_1896	0.070	0.257	0.187	0.0178	None	Hypothetical protein
group_1905	0.075	0.257	0.182	0.0248	None	Hypothetical protein
group_1897	0.080	0.257	0.177	0.0340	None	Hypothetical protein

Gene	Frequency SC11-pop I	Frequency SC11-pop II	Frequency difference of SC11 pop II – SC11-pop I	p adjusted	Recomb modular	Gene annotation
group_1898	0.080	0.257	0.177	0.0340	None	Hypothetical protein
pemK	0.050	0.200	0.150	0.0416	None	Endoribonuclease PemK
group_1715	0.050	0.200	0.150	0.0416	None	Hypothetical protein
ssb_3	0.980	0.829	−0.151	0.0077	None	Single-stranded DNA-binding protein
ssbA	0.980	0.829	−0.151	0.0077	None	Single-stranded DNA-binding protein A
aadK---aadK_1	0.960	0.800	−0.160	0.0182	None	Aminoglycoside 6-adenylyltransferase
group_1596	0.965	0.800	−0.165	0.0110	None	Hypothetical protein
group_1606	0.965	0.800	−0.165	0.0110	None	Hypothetical protein
COQ5_3	0.965	0.800	−0.165	0.0110	None	2-methoxy-6-polyprenyl-14-benzoquinol methylase mitochondrial
group_1594	0.970	0.800	−0.170	0.0064	None	Hypothetical protein
group_1602	0.975	0.800	−0.175	0.0034	None	Hypothetical protein
group_1604	0.975	0.800	−0.175	0.0034	None	Hypothetical protein

*Significance of frequency difference were test using Fisher's precision probability method. *p* adjusted with false discovery rate (calculated using the Benjamini-Hochberg procedure). Colors of shading corresponds to frequency values, with darker red indicating higher values and lighter green representing lower values, to emphasize comparative differences.

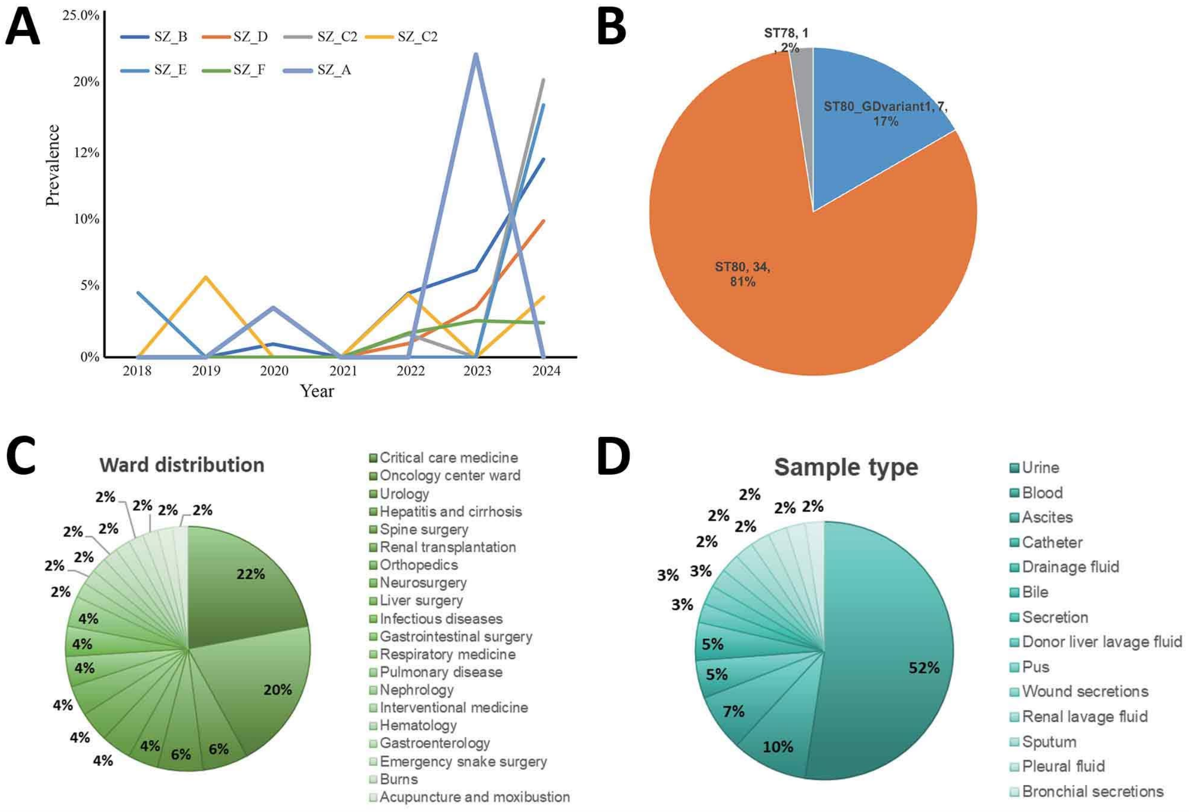

Appendix 1 Figure 1. Recently VREF circulating in Shenzhen. A) Increasing prevalence of VREF in Shenzhen, represented by 7 hospitals. The *x* axis indicates the prevalence in percentage; each line represents 1 hospital. B–D) Sequence type composition, ward distribution, and sampling sources of VREF (2018–2024).

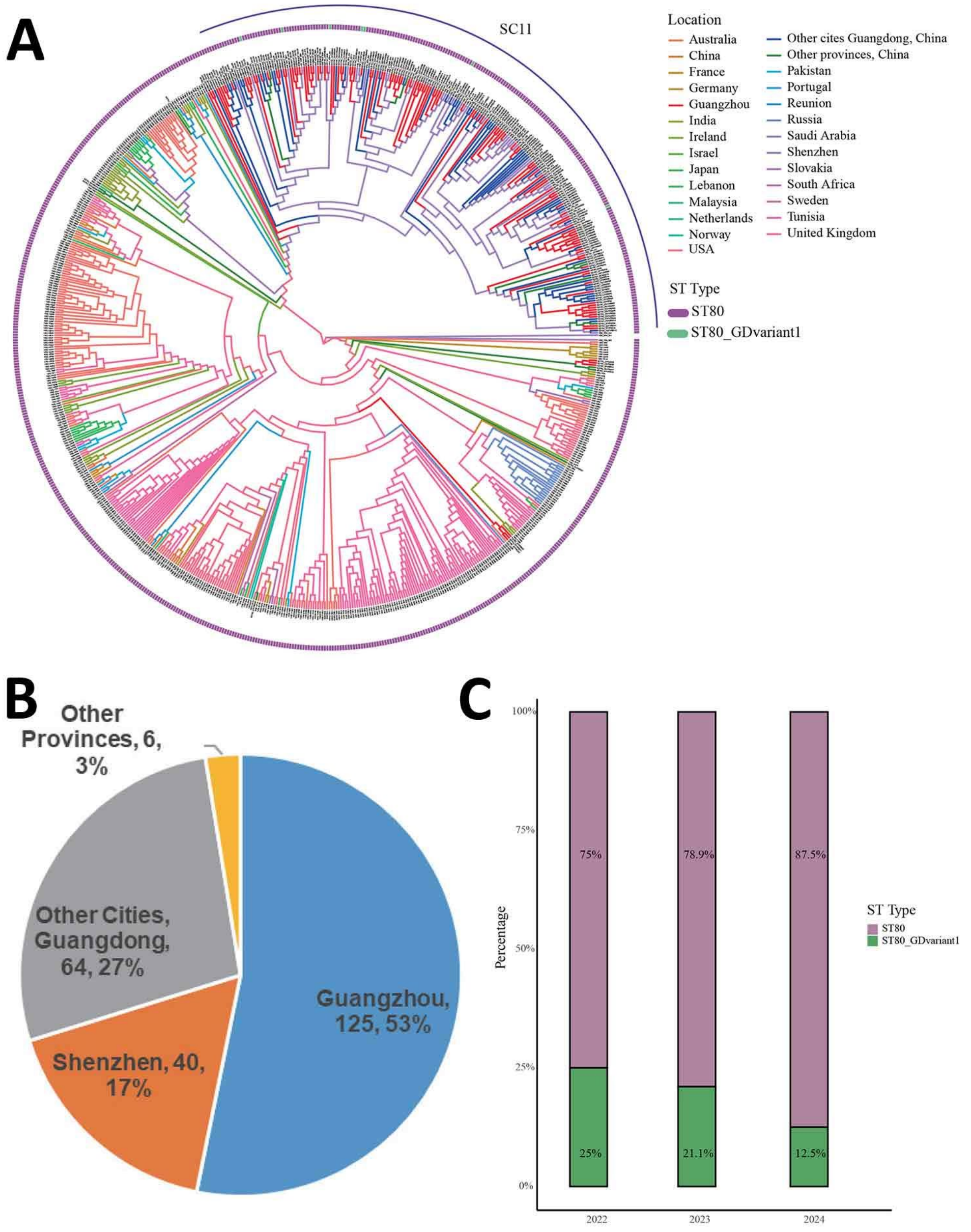

Appendix 1 Figure 2. Shenzhen circulating VREFs belongs to the ongoing SC11 epidemic in Guangdong Province. A) Maximum likelihood (ML) tree inferred from cgSNPs by incorporating publicly available data with recombination region masked. The circle around the tree indicated ST types by color, the purple arc on the upper right indicated whole SC11 lineage, and the country or regions of these isolates are colored in branch. Phylogenetic analysis was inferred from the cgSNPs, among genomes of these 41 ST80 and variants isolates, 484 public available ST80 isolates in BV-BRC (Bacterial and Viral Bioinformatics Resource Center) (until June 2023) (*15*), and ST80 isolates reported by Shen et al. (*9*). B) Geographic composition of members of the SC11 lineage in this epidemic. C) ST80_GDvariant1 occurred frequently in 3 years in Shenzhen.

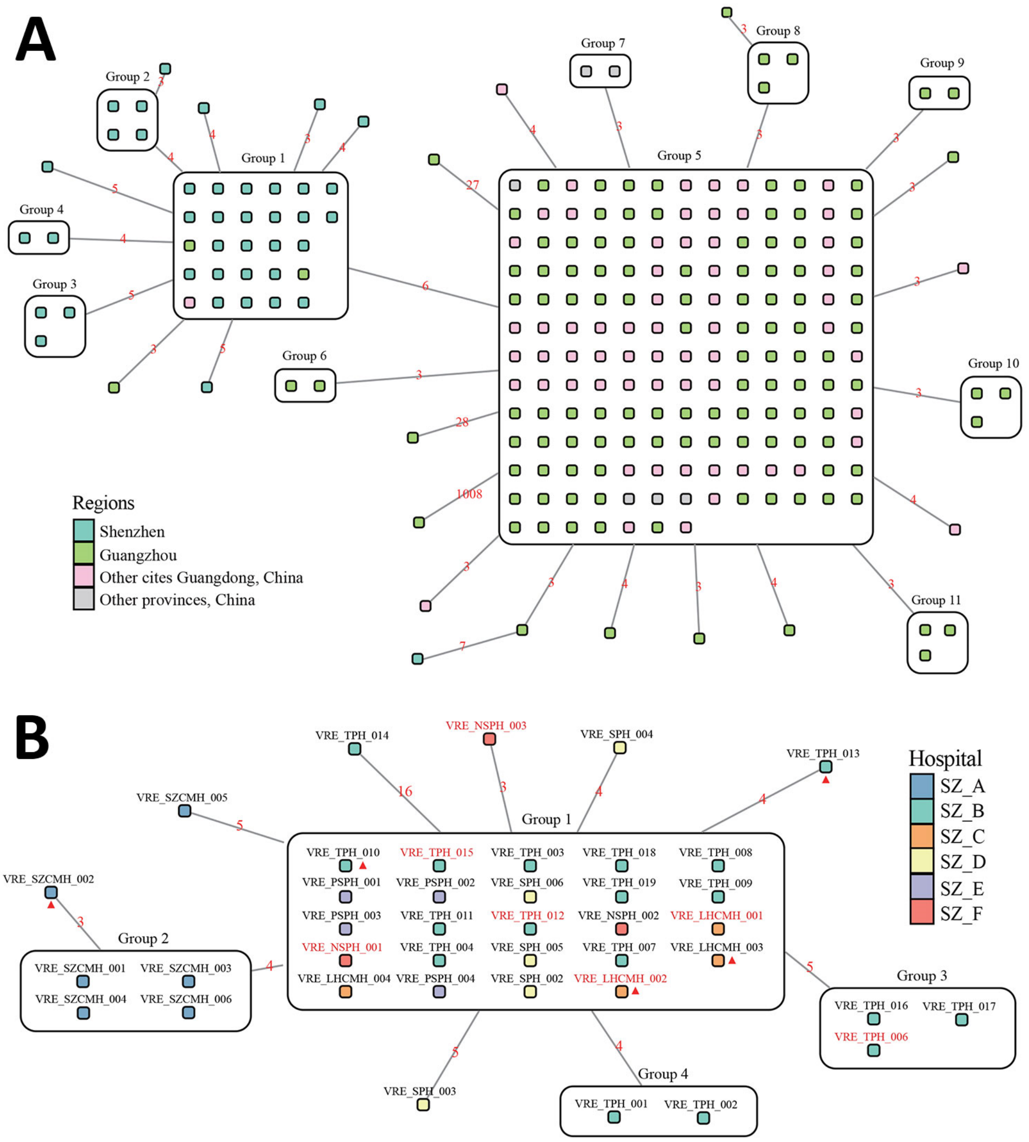

Appendix 1 Figure 3. Transmission relationship of (A) all available SC11 isolates and (B) SC11 isolates from Shenzhen inferred basing on pairwise cgSNP distances incorporating isolating time. City and hospitals are label with different colors. Strains in red font (B) are ST80_GDvariant1. Transmission was linked by lines among groups. Each group represents >1 strain that is closely related and of indistinguishable transmission relationship. Number on the line indicates the SNP distances between linked groups. Two nosocomial transmission groups in TPH, one lasting at least 30 days in the spine surgery ward (VRE_TPH_001 and VRE_TPH_002, isolated in March 12, 2024 and April 10, 2024, respectively) and the other lasting at least 265 days in the infectious disease ward (VRE_TPH_006, 2022-10-02), hematology ward (VRE_TPH_016, 2023-06-11), and ICU (VRE_TPH_017, 2023-06-23). One nosocomial transmission cluster in SZCMH lasted >60 days in the ICU (VRE_SZCMH_001, 003, 004, and 006, from May 5, 2023 to July 3, 2023).

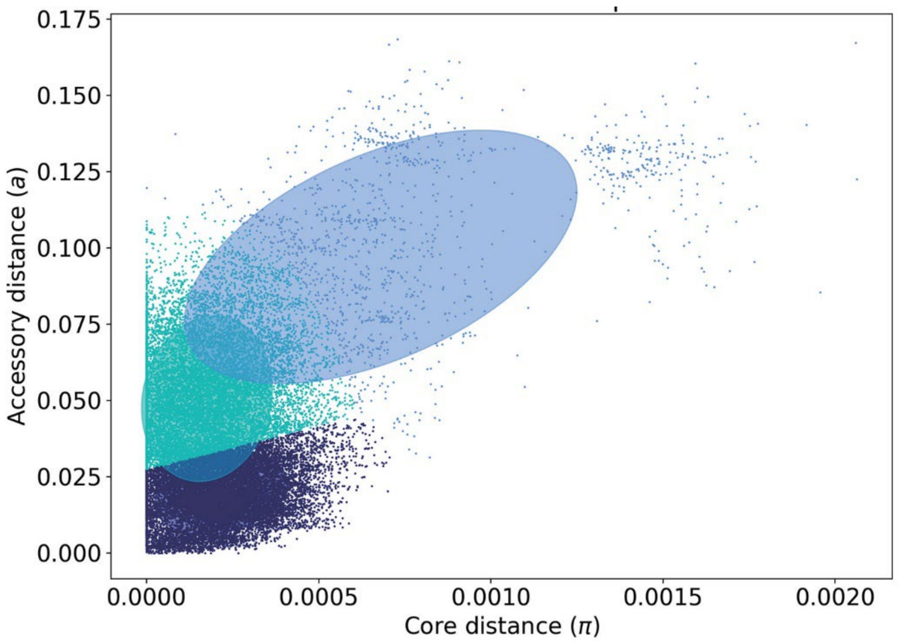

Appendix 1 Figure 4. SC11 clustered by core gene distance and accessary gene distance using the pgmm model under parameter *k* = 3 in PopPUNK. Each dot in the coordinate represents pairwise distance of 2 strains. Two major populations are delineated in blue and green.

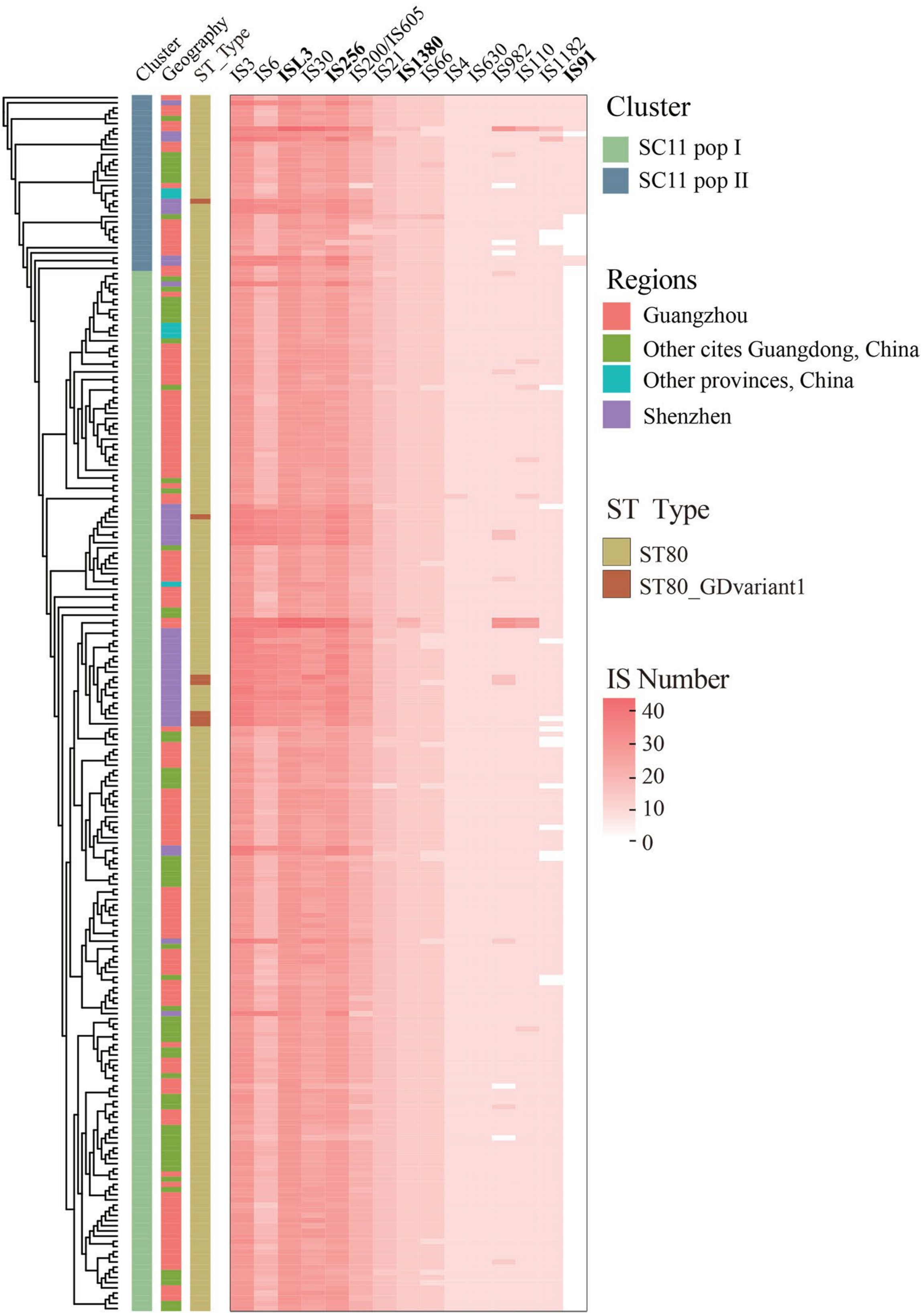

Appendix 1 Figure 5. Heatmap showing copy numbers of 10 IS elements in each isolate. The copy number of IS elements elevated in SC11-pop II compared with SC11-pop I are bold.

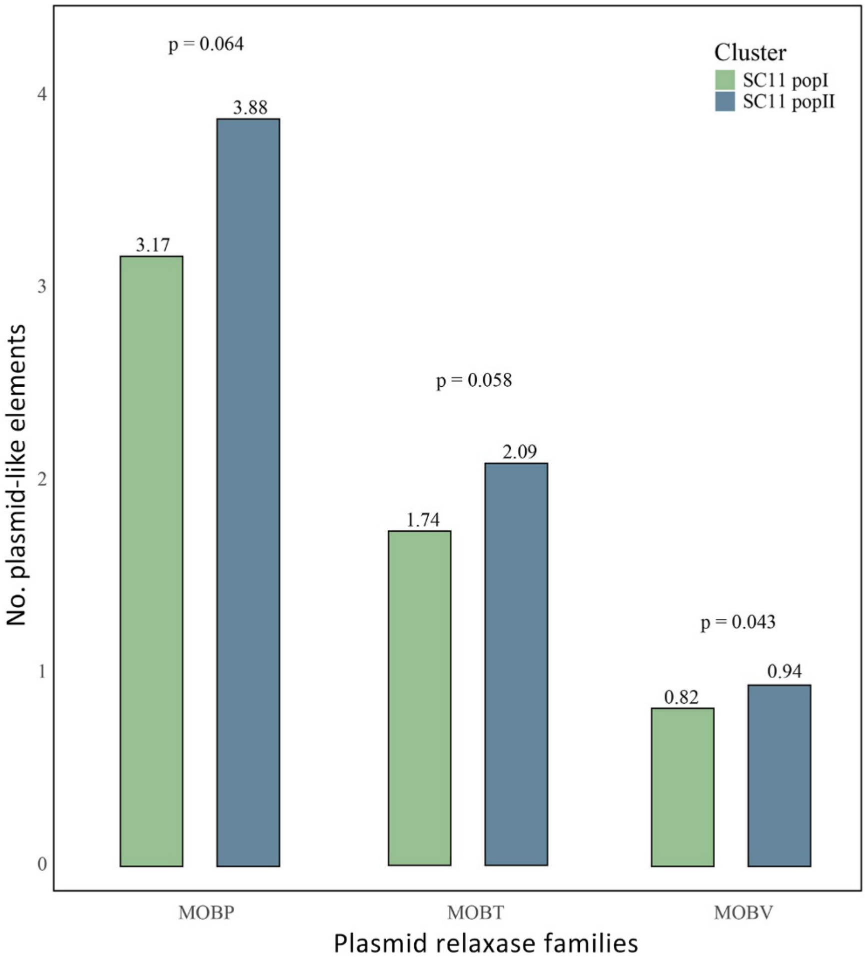

Appendix 1 Figure 6. Cluster 2 strains carried more MOB marked plasmid-like elements.
